# Laser Powder Bed Fusion Processing of Ti-6Al-4V Powders with Offsize and Wide Particle Size Distributions—Process Optimization

**DOI:** 10.3390/ma19143049

**Published:** 2026-07-15

**Authors:** Farzad Liravi, Mahyar Hasanabadi, Tatevik Minasyan, Pablo D. Enrique, Sebastian Soo, Farima Liravi, Mahdi Habibnejad-Korayem, Ehsan Toyserkani

**Affiliations:** 1 Multi-Scale Additive Manufacturing Laboratory, Department of Mechanical and Mechatronics Engineering, University of Waterloo, 200 University Ave. W., Waterloo, ON N2L 3G1, Canada; fliravi@uwaterloo.ca (F.L.); mahyar.hasanabadi@uwaterloo.ca (M.H.); pablo.enrique@uwaterloo.ca (P.D.E.); sjxsoo@uwaterloo.ca (S.S.); f2liravi@uwaterloo.ca (F.L.); 2 Department of Mechanical and Industrial Engineering, Tallinn University of Technology, 19086 Tallinn, Estonia; 3 AP&C, A Colibrium Additive Company, St-Eustache, QC J7R 0L5, Canada; mahdi.habibnejad@geaerospace.com

**Keywords:** additive manufacturing, 3D printing, laser powder bed fusion, design of experiments, offsize particle size distribution

## Abstract

The considerable expense associated with metal additive manufacturing (AM), partly attributed to the high costs of raw materials, forms a significant obstacle hindering the widespread adoption and scaling of this technology. In response to this challenge, this study endeavors to explore and optimize the laser powder bed fusion (LPBF) process parameters for Ti-6Al-4V powders with offsize (45–106 µm) and wide (15–106 µm) particle size distribution (PSD) which are more cost-effective. The outcomes will be compared to those of the same alloy with a standard 15–53 µm PSD. The primary focus of this investigation revolves around two key objectives: firstly, establishing correlations between the laser powder bed fusion process parameters and the resulting density, hardness, and roughness. This objective is achieved by investigating the impact of process parameters within the context of the contour–skin–core method. Secondly, the porosity, microstructure, elemental composition, and dimensional fidelity of several components made from the offsize and wide powders were investigated, utilizing the optimized process parameters for density. To this end, an efficient multi-step experimental design and optimization process was adopted. The findings resulted in the identification of correlations between the significant process parameters and the studied responses, enabling the achievement of 98.7% density and 40.6 HRC hardness for offsize powder and 98.7% density and 40 HRC hardness for wide powder. A separate set of optimized process parameters for larger geometries produced densities exceeding 99.9% in both as-built and HIP conditions across all three powders. Additionally, the results confirmed the higher sensitivity of the roughness to powder size, with the optimized values fluctuating between 9.5 µm and 15.7 µm. Comprehensive microstructural investigation reveals no significant differences in phase evolution or grain structure resulting from the use of offsize or wide powders. This study confirms the viability of utilizing powders containing a higher portion of large particles to mitigate the costs associated with LPBF processes.

## 1. Introduction

Despite the remarkable advancements in AM and its rapid growth over the past decade, several challenges persist, hindering widespread industrial adoption. Key among these challenges are: (1) the high costs of materials suitable for AM and the complexities of process development for new material systems; (2) limited machine reliability and real-time closed-loop quality assurance capabilities; and (3) the shortage of effective strategies to enhance productivity and scale-up [1]. This study is attempting to offer solutions for the first issue, aiming to provide an efficient solution for the process optimization of offsize and wide, and consequently less expensive, Ti-6Al-4V powders.

The properties of the powder systems employed in powder-based AM technologies have significant implications for the performance and characteristics of the resulting products [2,3]. LPBF, in particular, is highly sensitive to powder size and shape which in conjunction with powder chemical composition determine the packing density and thermal properties of each layer throughout the printing process [4], ultimately influencing the density and mechanical performance of the final product. Typically, a narrower range of powder, featuring a lower proportion of fine particles, enhances flow, while a certain fraction of fine powders is necessary to achieve a higher powder bed density, with smaller particles filling gaps between larger grains. On the other hand, the susceptibility to agglomeration increases due to the heightened influence of electrostatic forces on these finer particles. Consequently, there are numerous, and at times conflicting, factors to consider when selecting the optimal PSD [3]. The literature extensively explores the impact of powder size and morphology, including range, uni- or bi-modality, sphericity, surface texture, and more, on the rheological properties of bulk powder. For a more in-depth understanding, articles [5,6,7] serve as informative references.

Typically, LPBF involves the use of fine powders characterized by particle sizes represented by D10 and D90 values of 15 µm and 45 µm (or 53 µm), respectively [3,8,9]. Here, D10 and D90 represent the sizes at or below which 10% and 90% of all particles are located, respectively. However, a study on the printability and safety of offsize Ti-6Al-4V powders reveals that this PSD range might not be the most efficient [10]. According to this study, during powder production, powders with a broader PSD of 1–300 µm are commonly generated, with the 15–45 µm cuts of powder forming less than 30% of the total yield. The remaining portion of the batch, consisting of coarser particles, is usually discarded. On the other hand, a study by Kim et al. (2024) shows that incorporating larger particles to moderately increase the D90 to 75 µm could reduce powder costs by approximately 20%, revealing the potential cost-saving benefits of utilizing even wider PSDs containing larger particles [11].

A review of the literature highlighted in [3,12] indicates that most studies investigating the effects of PSD or powder rheology on part properties still focus on powders within the standard LPBF range, with D90 typically below 60 µm. Spierings et al. (2009) examined three PSDs of 316L stainless steel (6.3–30.8 µm, 19.9–41.3 µm, and 15.6–59.7 µm) in LPBF, showing that adjusting scan speed can achieve densities above 99.5% despite potential negative impacts on productivity [2]. Their subsequent work revealed the importance of finer powders for improved surface appearance and tensile strength, while larger particles contribute to higher elongation at break [13]. Liu et al. found that the presence of finer particles within a batch of 316L stainless steel (0–45 µm) enhances density and exhibits better performance in terms of ultimate tensile strength (UTS) and elongation at break compared to a narrow and coarser PSD of the same powder (15–45 µm) [4]. They, however, reported an inconsistent relationship between PSD, process parameters, and properties for surface roughness and hardness. Stegman et al. (2023) observed insignificant changes in density, microstructure, and mechanical behavior between standard (18.7–43.6 µm) and wider bi-modal (14.5–50.8 µm) Inconel 718 [8].

As mentioned, the existing literature provides limited insights into the effects of offsize or wide powders with a higher fraction of coarse particles on part properties. In a 2004 study, Simchi et al. tested iron powders with D10 values exceeding 120 µm, revealing a noteworthy density reduction of up to 50% [14]. Abd-Elghany et al. employed a low-cost gas-atomized 304L powder with a D90 of 106 µm, characterized by a highly irregular and non-spherical shape. Under optimized processing parameters, they found that the manufactured parts exhibited UTS at 70% of the standard AM counterpart material [15]. Therefore, these works employing powder with particles larger than 60–100 µm have usually demonstrated a detrimental effect on several properties. Habibnejad-Korayem et al., however, demonstrated that the microstructure, mechanical properties, and porosity of the parts printed using Ti-6Al-4V with offsize PSDs were largely comparable to the benchmark 15–45 µm powder [10]. In a separate study, Kim et al. demonstrated that LPBF processing Ti-6Al-4V using a PSD of 15–75 µm achieves mechanical properties slightly below those of the standard 15–53 µm PSD but still in compliance with Metallic Materials Properties Development and Standardization (MMPDS) guidelines [11]. Building on previous work in the literature, this study aims to optimize the process parameters for Ti-6Al-4V with offsize and wide PSDs for a more comprehensive evaluation of process–property relationships.

**Figure 1:**
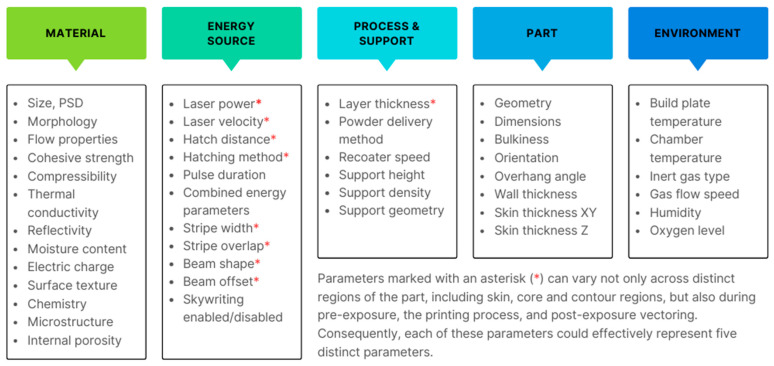
The LPBF process parameters, classified into material, energy source, process and support, part, and environment categories. The items listed under each category are derived from the information available in [3,43,44].

Upon reviewing the published literature on LPBF, it becomes evident that researchers predominantly concentrate on a limited set of two to four process parameters in their process optimization studies with two-, three-, and mixed-level factorial methods constituting the main statistical designs of choice [16,17,18,19,20,21,22,23,24,25,26,27,28,29,30,31,32,33], followed by CCD (central composite design) [34,35,36,37,38] and Taguchi [39,40,41]. These parameters usually include laser power, velocity of the laser beam, layer thickness, and hatching distance. These parameters, sometimes supplemented by energy density, are hereafter referred to as the primary parameters. Although the wealth of research in this area confirms these primary parameters as the most influential factors on the melt pool stability and geometry, there are drawbacks associated with a narrow scope of parameters, particularly in the study of mechanical properties [42]. Figure 1 displays a non-exhaustive list of parameters found in an LPBF process, categorized as material, energy source, process and support structure, part, and environment.

As pointed out in Figure 1, various parameters, such as laser power, may vary depending on the print location (skin and core) and the order of exposure (pre-, core-, and post-vectoring). While some articles have demonstrated the application of distinct print parameters for contour, skin, and core regions [45,46,47], commonly referred to as the skin–core method, we have not identified any studies that delve into the impact of these region-based parameters on the properties of the manufactured parts.

**Figure 2:**
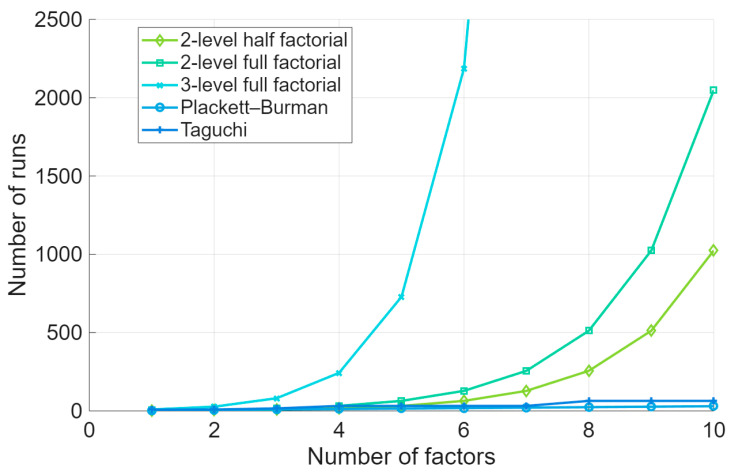
Comparison of the required number of experimental runs for different numbers of factors in commonly used experimental design techniques. For the Taguchi method, the most commonly used orthogonal array for each number of factors is considered.

The main reason for focusing on studying only the primary parameters, however, lies in the fact that considering a large number of parameters in traditional factorial designs (FDs) can lead to prohibitively high experimental costs and time-consuming efforts. For instance, with a two-level FD, the required number of runs for just one replication of the design increases exponentially as the number of parameters grows. This study employs a sequential experimental design and optimization approach, integrating conventional FD methods with the Plackett–Burman (P-B) method. The P-B method enables the evaluation of numerous input factors while minimizing the required number of experimental runs. To gain a clearer perspective, the graph in Figure 2 illustrates the number of runs required for some commonly used DoE techniques for different numbers of factors.

The results presented in this study demonstrate that utilizing non-standard, more cost-effective, and safer powder size distributions (PSDs) can yield material properties comparable to standard PSDs, thereby lowering the economic barriers to AM adoption. To systematically present these findings, this article is divided into two primary components: a theoretical focus on statistical modeling and a sensitivity analysis, followed by a comprehensive experimental validation. Within this framework, Section 3.1 presents the optimal process parameter sets for three distinct PSDs (15–53 µm, 15–106 µm, and 45–106 µm) across three target responses (density, hardness, and roughness). Finally, Section 3.2 provides an in-depth examination of the porosity, microstructure, elemental composition, and dimensional accuracy of parts fabricated using these optimized density parameters.

## 2. Materials and Methods

### 2.1. Materials and Powder Characterization

**Figure 3:**
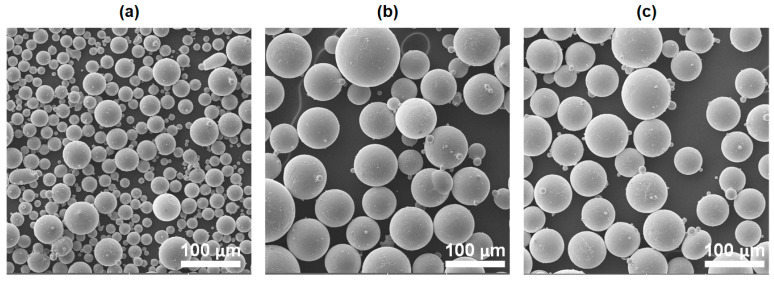
SEM micrograph for: (**a**) Powder A [15–53 µm], (**b**) Powder B [15–106 µm], and (**c**) Powder C [45–106 µm].

Three distinct PSD cuts of Grade 23 plasma-atomized Ti-6Al-4V, supplied by AP&C (Montreal, QC, Canada), were used as received. These powders are denoted as Powder A (15–53 µm), Powder B (15–106 µm), and Powder C (45–106 µm).The powder morphology was verified using a scanning electron microscopy (SEM) system (VEGA3, TESCAN, Brno, Czechia), operating at an accelerating voltage of 15 kV, magnification of 500×, and a working distance of 31.9 µm. The SEM micrographs for all three powders, depicted in Figure 3, show highly regular and spherical particles.

**Figure 4:**
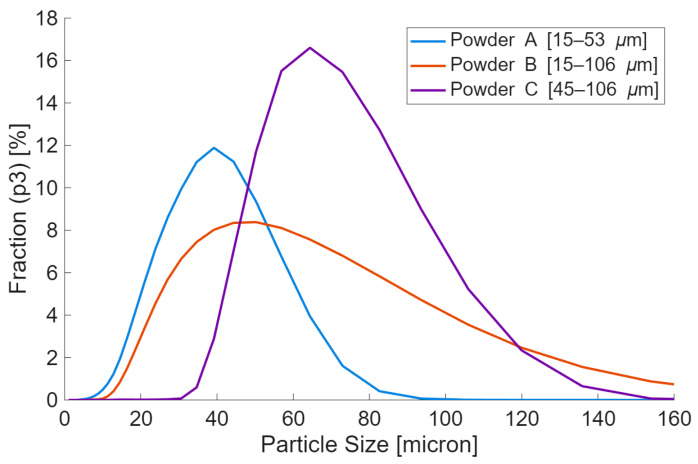
Particle size distribution curves for Powders A, B, and C.

The PSD and morphology of each batch of powder were assessed three times through the utilization of a particle size and shape analyzer (CAMSIZER X2, RETSCH GmbH, Haan, Germany). The particle diameters corresponding to the 10th percentile (D10), 50th percentile (D50), and 90th percentile (D90) of the cumulative size distributions, as well as the sphericity values, are presented in Table 1. The PSD for all three powders is shown in Figure 4. The chemical composition of these powders, as provided by the vendor, is documented in Table 2. 

### 2.2. Statistical Analysis

In this work, the effect of the 23 process parameters on the density, hardness, and surface roughness of test specimens made from Powders A, B, and C was investigated. These parameters were selected based on a combination of factors: key parameters identified in the literature, those that can be adjusted within a single build, system constraints, and the field expertise of the authors. The selected parameters are organized into six groups:

1. Pre-exposure parameters: The speed and power applied for vectoring the contour of each layer before initiating particle consolidation with high energy density.
2. Post-exposure parameters: The speed and power applied for vectoring the contour of each layer after completing the printing process for that layer.
3. Skin exposure parameters: The power, energy density, hatch distance, stripe width, stripe overlap, and thickness values applied to the outermost region of each part known as the skin.
4. Core exposure parameters: The power, energy density, hatch distance, stripe width, and stripe overlap relevant to the core of the printed part. For components produced through the chess-hatching strategy, the stripe width corresponds to the square width, while the stripe overlap aligns with the square gap overlap.
5. Support parameters: The height and density of the support structures.
6. Other parameters: Layer thickness, angle, geometry, hatching method, and location of the parts.

Notably, in this study, the skin and core speeds have been replaced with energy density to maintain the energy applied during the print within an acceptable range, preventing build failure. The areal energy density adopted in this study is defined as

(1) $$ED=\frac{power}{speed\times hatch\;distance}$$

**Figure 5:**
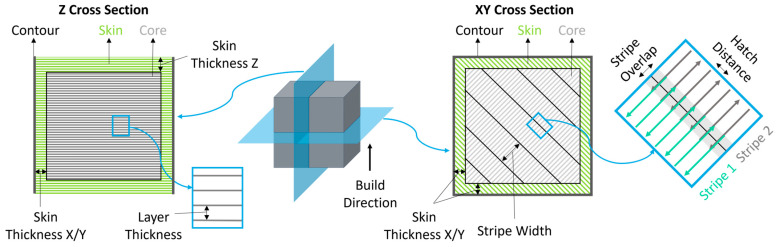
Visual representation highlighting process parameters that are less commonly explored in the stripe-hatching method. Comparable concepts of stripe overlap, stripe width, and hatch distance can be envisioned for the skin region, although they are not illustrated here. In the chess-hatching method, which is not depicted in the figure, a square width is akin to stripe width, extending in two directions to define the sides of a square. Likewise, a square overlap mirrors stripe overlap, but is applied along the periphery of a square.

Figure 5 illustrates the three regions of contour, skin, and core, as well as the concepts of stripe width, stripe overlap, and hatch distance.

Two experimental design techniques, namely P-B and fractional factorial design (FFD), within the broader framework of response surface methodology (RSM), were employed. The P-B method, introduced by R. L. Plackett and J. P. Burman in 1946 [48], was chosen as the best approach for initial vetting of the parameters. After eliminating the process parameters that are not statistically significant using the P-B design and reducing the number of parameters to a more manageable figure, a second model using two-level FFD has been designed to learn more about the process. This approach is an adaptation of the optimization procedure outlined in our previous works [49,50], with some adjustments made to suit the objectives of the current study.

The DoE step was followed by numerical optimization utilizing the desirability function method (DFM). In DFM, each response’s value is transformed into a numerical range between zero and one, referred to as desirability, where values closer to one indicate superior outcomes. The desirability functions for minimization, maximization, and target analyses are given by Equations (2)–(4):

(2) $$d\left(y_{i}\right)=\left\{\begin{array}{cc} 1 & for\;y_{i}<t_{i}\\ {\left(\frac{y_{i}-u_{i}}{t_{i}-u_{i}}\right)}^{s} & for\;t_{i}\leq y_{i}\leq u_{i}\\ 0 & for\;y_{i}>u_{i} \end{array}\right.$$

(3) $$d\left(y_{i}\right)=\left\{\begin{array}{cc} 0 & for\;y_{i}<l_{i}\\ {\left(\frac{y_{i}-l_{i}}{t_{i}-l_{i}}\right)}^{s} & for\;l_{i}\leq y_{i}\leq t_{i}\\ 1 & for\;y_{i}>t_{i} \end{array}\right.$$

(4) $$d\left(y_{i}\right)=\left\{\begin{array}{cc} 0 & for\;y_{i}<l_{i}\\ {\left(\frac{y_{i}-l_{i}}{t_{i}-l_{i}}\right)}^{s} & for\;l_{i}\leq y_{i}\leq t_{i}\\ {\left(\frac{y_{i}-u_{i}}{t_{i}-u_{i}}\right)}^{t} & for\;t_{i}\leq y_{i}\leq u_{i}\\ 0 & for\;y_{i}>u_{i} \end{array}\right.$$

where $d\left(y_{i}\right)$ is the desirability function, $y_{i}$ is an individual response, $l_{i}$ is the lower limit, $t_{i}$ is the target value, and $u_{i}$ is the upper limit. The exponents s and t determine the shape of the function: linear when equal to 1, concave for values greater than 1, and convex for values smaller than 1. The overall desirability function is calculated as follows:

(5) $$d_{total}={\left[d\left(y_{1}\right)d\left(y_{2}\right)\ldots d\left(y_{i}\right)\right]}^{1/i}$$

Three steps are completed for each powder according to the following labeling scheme:

The statistical analyses and optimization were primarily executed using Minitab 21.4.1 (Minitab LLC, State College, PA, USA). MATLAB R2021b (MathWorks, Natick, MA, USA) was used to plot the results of the analyses.

### 2.3. LPBF Process Setup

**Figure 6:**
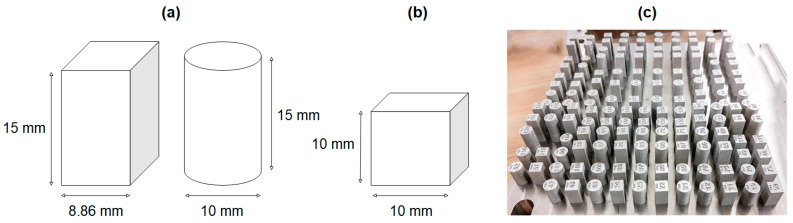
Geometries used in: (**a**) A1-2 and B1-2 builds, and (**b**) A2-3, B2-3, and C1-3 builds. (**c**) A view of the as-printed design A2 before removal of the parts from the build plate.

As shown in Table 3, multiple experiments were designed to optimize the process parameters for each powder. For Powders A and B, cylindrical and cuboid geometries of the same cross-sectional area were printed, as illustrated in Figure 6a. To simplify and expedite the characterization, the geometry of the coupons was altered to 10 × 10 × 10 mm^3^ cubes, as shown in Figure 6b, for Powder C. The process parameters for each step are provided in the Supplementary Information (SI).

After completing the optimization study, several artifacts with different geometries were printed using the process parameter sets re-optimized for density with the core power limited to 200–250 W and used for porosity and microstructure investigation. These artifacts include cylindrical bars (Ø8 mm × H80 mm) and several other moderately complex geometries including pins, brackets, etc. Specific details of these geometries cannot be shared due to confidentiality constraints. The optimized process parameters used to fabricate artifacts are presented in Section 3.1.3.

Additionally, a series of walls with thicknesses ranging from 400 µm to 1000 µm, gaps with widths ranging from 50 µm to 1000 µm, and drill holes with diameters ranging from 250 µm to 8000 µm were printed using Powders B and C to assess dimensional accuracy. The artifacts intended for dimensional accuracy evaluations were also printed using process parameters optimized for density with constrained core power, as presented in Section 3.1.3.

The geometries were designed using SolidWorks 2021 (Dassault Systèmes, Vélizy-Villacoublay, France). The build layouts and support structures were created using Magics Software v25.01 (Materialise NV, Leuven, Belgium). The build plate was pre-heated to 80 °C, and the build chamber was filled with argon at a differential pressure of 79 mBar. The beam diameter measured 80 µm. The specific LPBF machine used for printing these parts remains undisclosed in accordance with the confidentiality terms of the study. In all print runs, the original virgin powder was used, supplemented with manually sieved used powder if necessary. Parts were removed from the build plate using a wire electrical discharge machining (EDM) system (VL600QH, Sodick, Yokohama, Japan), and the support structures were manually cut.

### 2.4. Hot Isostatic Pressing (HIP)

A portion of the cylindrical bars (Ø8 mm × H80 mm) were HIP processed at 900 ± 25 °C under 100 ± 1.72 MPa pressure for 2 h. Individual parts were wrapped in stainless steel foils to protect against oxidation, although all specimens were machined after the HIP post-processing. The HIP was performed by an external provider.

### 2.5. Characterization of Components

Several properties of the printed components including density, hardness, surface roughness, microstructure, elemental composition, and dimensional accuracy were characterized at various stages of this study.

The density measurements for components printed for the DoE study were carried out based on the Archimedes’ Principle as described in ASTM B311-17 standard using a Buoyancy Density Kit (Secura, Sartorius AG, Göttingen, Germany). The density measurements for each part were repeated three times and an average value was used in statistical analyses. The density of larger artifacts was measured through optical image analysis (VHX-7000 digital microscope, Keyence, Osaka, Japan) on polished surfaces. The specimens were sectioned using a precision cutter (IsoMet 1000, Buehler, Lake Bluff, IL, USA), hot mounted with a mounting press (CitoPress 5, Struers, Copenhagen, Denmark), and ground and polished with an autopolisher (LaboPol-20, Struers, Ballerup, Denmark).

Hardness testing was conducted using a Rockwell hardness tester (Phase II+ 900-331, Phase II, Upper Saddle River, NJ, USA) following the ASTM E18-20 standard for Rockwell C testing. Five hardness indentations were performed on each sample, and the values were averaged. To ensure consistency, indentation areas were chosen on the vertical plane of the components, running parallel to the gas flow and perpendicular to the recoater’s movement. Before performing the hardness tests, the specimens were ground using an autopolisher with silica carbide (SiC) papers rated at FEPA P grade #80 to ensure a reasonably flat surface for accurate indentations. For cuboid samples, both the target surface and its opposite side underwent manual grinding, while cylindrical samples had one side manually ground.

**Figure 7:**
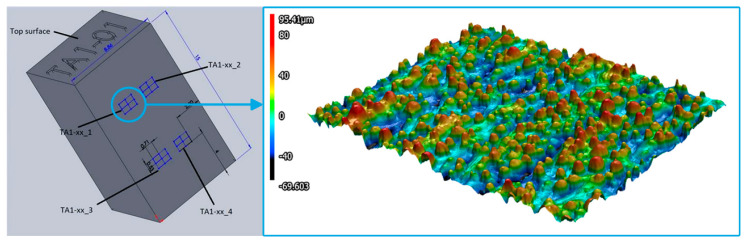
The areal surface-roughness values are averaged over several smaller scan areas located in the core region of the specimens.

The areal surface roughness (Sa) of the parts was assessed using a laser-confocal microscope (VK-X250, Keyence, Osaka, Japan) in compliance with ISO 25178 standard at 20× magnification [51,52]. For designs A1 and B1, measurements of surface roughness were conducted at 16 designated locations as shown in Figure 7. For the smaller geometries adopted in the remaining designs, scans were performed at 9 adjacent areas. Each scan area measured 709.6 µm (x-axis) by 532 µm (y-axis), corresponding to the microscope’s field of view at the chosen magnification. To ensure consistency, scan areas were chosen on the vertical plane of the components, running parallel to the recoater’s movement and perpendicular to the gas flow. These selected scan areas were centrally positioned to avoid the areas near the edges. The resulting images were stitched together using VK Image Stitching software (Version 2.1.1.0) (Keyence, Osaka, Japan), creating a larger composite surface area. Prior to evaluating roughness values using MultiFileAnalyzer software (Version 1.3.1.120) (Keyence, Osaka, Japan), a series of pre-measurement processing steps were implemented. These pre-processing steps included reference-plane correction to rectify any tilts on flat cuboidal surfaces and surface-shape correction to eliminate curvature on cylindrical samples. A second curve-surface correction method was applied across the entire scanned area to address curvature-related issues. Furthermore, a Gaussian S-filter (low-pass filter), with a specific value of 2.1 µm (three times the XY resolution), was applied to the scans before proceeding with surface roughness calculations.

Sample preparation for electron microscopy analysis included vibratory polishing (VibroMet 2, Buehler, IL, USA) with a finely dispersed aluminum oxide suspension (MasterPrep, Buehler, IL, USA) for approximately 4 h. This was followed by polishing with a non-crystallizing amorphous colloidal silica suspension (MasterMet 2, Buehler, IL, USA) for another 4 h. Energy-dispersive spectroscopy (EDS) analysis was conducted using the TESCAN VEGA3 SEM equipped with a BRUKER XFlash 6I10 detector (Bruker Nano GmbH, Berlin, Germany). For electron backscatter diffraction (EBSD) scanning and post-processing, a BRUKER e-Flash FS detector and the open-source software MTEX-11.5.2 were used, respectively [53].

X-ray diffraction (XRD) data were collected using a diffractometer (SmartLab XE, Rigaku, Tokyo, Japan) equipped with a 3 kW Cu sealed tube source (λ_avg_ = 1.54056 Å), operating at 40 kV and 50 mA, and utilizing CBO optics in Bragg–Brentano geometry with phi rotation. Scans were performed over a 2θ range of 20–75° with a step size of 0.01° and a scan speed of 0.6°/min, using SmartLab Studio II v4.6.182 software (Rigaku, Japan) for data acquisition.

To investigate the dimensional accuracy of the printed parts, the samples were cut from the build plate, manually ground and polished using FEPA 80 to 4000 grit size SiC abrasive papers. The dimensional measurements were performed using ImageJ software (Version 1.54g) based on optical micrographs [54].

## 3. Results and Discussion

### 3.1. Process Modeling

#### 3.1.1. Plackett–Burman Designs

The P-B analyses (designs A1, B1, and C1) are detailed in Sections S1–S3 of the Supplementary Information document with the most statistically significant process parameters summarized in Table 4. Each P-B design was separately analyzed three times corresponding to the three responses. Reduced-model analysis of variance (ANOVA) results for density, hardness, and roughness are summarized in Tables S3, S7 and S13; S4, S8 and S14; and S5, S9 and S15 respectively.

For density, core power and core energy density were highly influential across all designs, alongside geometry for A1 and B1, print location for A1, support height for B1, and layer thickness for C1 (where geometry and angle were excluded). For hardness, geometry, skin thickness Z, core power, and skin power were the most significant parameters for both A1 and B1 (with A1 also influenced by skin energy density). In C1, layer thickness, core power, support height, skin stripe overlap, and skin thickness Z were significant. For roughness, A1 was most affected by skin thickness XY, support height, core stripe width, skin thickness Z, and layer thickness. No parameters were significant at the 95% confidence level for Powder B (though skin thickness XY, core hatch distance, core stripe width, core stripe overlap, and layer thickness showed the highest impact in B2). For C1, skin energy density, skin power, and layer thickness were the most influential of the 14 significant parameters.

Designs A1 and B1 appear remarkably similar, sharing 14 significant parameters for density and 11 for hardness, though only 5 for roughness. This commonality does not extend to design C1, likely due to the removal of angle and geometry, altered specimen dimensions, and the absence of finer powders (see Supplementary Information Section S3).

Overall, the primary parameters (core power, core energy density, core hatch distance, and layer thickness) were statistically significant across most responses regardless of powder type, aligning with the LPBF literature. Additionally, this work highlights that under a skin–core strategy, density is predominantly influenced by primary core parameters, hardness by a combination of core and skin parameters, and roughness primarily by skin parameters.

A key interpretation that can be drawn from the P-B results is that, in each powder–response scenario studied here, there are several parameters whose impact on a specific response is not readily apparent or easily interpreted based on the governing physical principles of LPBF. Notable examples include support parameters for density in A1 and B1 or core hatch distance for roughness in A1 and C1. Although there might be physical explanations for these interactions, such as the height/density of the support structure influencing heat transfer within the build part and potentially affecting the thermal history, these explanations lack extensive study and cannot be confidently accepted. A more plausible explanation is that some of these parameters may not be genuinely significant. The reason the ANOVA highlights them as significant lies in the limitations of the P-B models. P-B is an important statistical tool for multifactorial modeling with a minimal number of experiments, but this comes at the expense of precision and design resolution. P-B is a highly confounded design (resolution III), i.e., it can only identify the statistically significant main factors and not consider the effect of interactions. For instance, it resolves whether power or velocity affects the density but does not reveal whether the interaction of power and velocity would also affect this response. On the other hand, it is common for P-B designs to identify a large number of parameters as significant, as seen in our case with a number of parameters between 6 and 17 flagged as significant. This contradicts the statistical principle of sparsity, as outlined by Montgomery et al. [55]. According to their article, the likely presence of significant two-factor interactions, which P-B is not capable of detecting, may distort the model, causing some of the insignificant parameters to be erroneously identified as significant. Therefore, a higher-resolution FFD is employed in the next step to further screen out insignificant parameters.

#### 3.1.2. Fractional Factorial Designs

As separate analyses of P-B designs were conducted for each response, following the principle of sparsity, the combination of parameters to be further examined in the FFD stage was strategically chosen to include the top four or five process parameters identified as significant for each individual response. This approach reduced the number of required FFD experiments from 9 to 3, i.e., one experiment per each powder as opposed to one per each powder–response combination. Additionally, it enables the adjustment of parameters based on the field knowledge. As can be seen in Tables S6, S10 and S16, a total of 14, 15, and 13 parameters were selected for FFD modeling in designs A2, B2, and C2, respectively. Interestingly, after the parameters are combined for FFD study, a high degree of overlap between the parameters selected for three powders can be observed. For example, core power, core energy density, skin stripe overlap, post-exposure contour power, support height, skin energy density, skin thickness Z, layer thickness, and hatching method are selected for all three powders for FFD modeling.

Reduced-model ANOVA results and regression coefficients for density, hardness, and roughness are summarized in Tables S19, S24 and S29; S20, S25 and S30; and S21, S26 and S31, respectively.

For density, the primary significant main factors for A2 were core power and core energy density, followed by layer thickness and skin energy density. Notably, the skin energy density x layer thickness interaction exerted an even higher influence, with an F-value roughly 1.5 times that of core power. Because these resolution IV designs confound second-order interactions with each other, pinpointing the exact active interaction is challenging without further de-aliasing experiments; this specific interaction is confounded with four other terms, several of which contain highly significant main factors. Design B2 shared these same key parameters but recognized more overall factors and interactions, with the confounded group represented by skin energy density x support height showing the largest effect. In C2, the top factors were layer thickness, core power, and the confounded core energy density x core power group.

For hardness, A2 and B2 showed strong similarities, both sharing geometry, skin energy density, skin thickness XY, skin hatch distance, and layer thickness as significant factors. However, their significant interactions diverged: A2 was dominated by interactions of skin energy density with skin thickness XY, skin thickness Z, and layer thickness, whereas B2 involved groups represented by post-contour power x core hatch distance, skin energy density x support height, and skin thickness Z x hatching method. Consistent with previous findings, C2 exhibited fewer significant terms, with layer thickness, core energy density, skin power, and the skin energy density x skin power interaction identified as significant.

For roughness, both the significant parameters and the relative weights of main effects versus interactions varied widely among the three powders. In A2, the effects of the significant main factors (skin thickness Z, skin thickness XY, and support height) were overshadowed by interaction groups, including post-contour power x layer thickness, skin energy density x core energy density, and skin energy density x skin thickness XY. Design B2 exhibited a more balanced weight between main factors (print location, skin energy density, post-contour power) and interactions. Conversely, in C2, the main factors (skin power, skin energy density, layer thickness) heavily dominated over second-order interactions like skin energy density x skin power.

It is noteworthy that the value of adjusted R-sq for roughness models is generally lower than that of density and hardness (refer to Table S29). Although, the values of adjusted R-sq for roughness are still within an acceptable range for AM (above 60%), given the inherent higher number of unexplained variations in these processes, the density and hardness models are expected to exhibit a higher goodness of fit. The authors suspect this is due to the limitations of areal roughness measurements in AM and the lack of standards for such, as well as the noisy nature of roughness data.

Additionally, the curvature term has been deemed significant in most designs. This observation suggests the influential presence of at least one quadratic term that needs to be added to the regression model through further experiments, such as those employing CCD.

#### 3.1.3. Optimization and Validation

After completing the ANOVA for the 9 FFD models assessing three physical properties across three powders, the number of statistically significant main parameters was reduced to fewer than 10 in all models, except the density model for Powder A, which retained 12 significant parameters. Additionally, the models also identified significant second-order interactions. The ANOVA models obtained in this stage became the basis for establishment of linear empirical regression models for each powder–response combination. The regression coefficients for significant main and second-order interaction terms for Powders A, B, and C are displayed in Tables S19, S24 and S29, respectively.

The DFM was employed to obtain the optimized combination of parameters for each response. This optimization technique is widely used when multiple responses need optimization. In the current study, the same method is applied to optimize individual responses separately. This investigation aims to achieve a target hardness of 41 HRC, a target density of 100% (corresponding to a nominal density of 4.43 g/cm^3^), and a target roughness matching the minimum roughness observed in designs A2, B2, and C2. Despite the anticipated hardness range for Ti-6Al-4V being between 30 and 36 HRC, the literature findings indicate values in the low 40s HRC for this alloy when subjected to LPBF processing [56,57,58]. Consequently, 41 HRC was chosen as the designated target hardness value. For all responses, a unified weight was assigned to all parameters with no limitations imposed on them. The optimized process parameters, along with the corresponding optimum response values and 95% prediction intervals for all studied cases, are provided in Table 5.

The predicted results show that the optimized process parameter sets should lead to almost full density for all three powders (~100% for Powders A and B, and 99.36% for Powder C) and hardnesses close to 41 HRC, regardless of the powder PSD. This is highly promising, indicating that by systematically optimizing the process, it is possible to use safer and more economical offsize powders with larger particles in LPBF to produce parts with density and hardness comparable to those made with the standard 15–53 µm PSD.

The obtained optimized density values also appear to be superior to those previously reported in the literature for offsize or wide powders [14]. However, a direct comparison is not possible due to the use of different alloys and/or machines. It also seems that the optimization process has led to a notable enhancement in the maximum achievable hardness for these powders. Previously reported in the literature at 340–343 measured by Vickers hardness (VH) (approximately 34.5 HRC) [10], the hardness is now expected to be ~41 HRC. However, it is essential to note that the comparison may lack precision, as the hardness assessment in the referenced article relies on Vickers testing, while the present study employs the Rockwell C method. In the absence of specific standard conversion tables between HRC and HV for titanium alloys, the ASTM E140 conversion intended for steels [59] was employed to provide an approximation of HRC values, as has been previously done by other researchers [60]. This correlation was further corroborated by a linear regression model correlating HRC and VH (Equation (6)) for six stable alpha–beta titanium alloys, including Ti-6Al-4V [61]:

(6) HRC=0.078VH+8.1

The optimized roughness values are different and increasing as the fraction of larger particles increases. The average optimized roughness for parts made with Powder A are predicted to be 9.5 ± 2.3 µm for 95% of the time. This will increase to 11.7 ± 2.5 µm and 15.6 ± 2.3 µm for Powders B and C, respectively. The results indicate that roughness is the most sensitive parameter among those studied to the changes in PSD, aligning with the existing literature.

To evaluate the predictive accuracy of the models in Table 5, 10 additional test specimens (10 mm × 10 mm × 10 mm cubes) were printed using each set of optimized process parameters (designs A3, B3, and C3). Their measured values were compared to the predicted values for each powder–response combination. Table 6 summarizes these observations, presenting their root mean square error (RMSE) relative to the predicted values as well as the prediction accuracy, calculated using Equations (7)–(9):

(7) $$RMSE=\sqrt{\frac{1}{n}\sum_{i=1}^{n}{\left(y_{i}-\hat{y}\right)}^{2}}$$

(8) $$Prediction\;Accuracy=1-Mean\;Absolute\;Percentage\;Error\left(MAPE\right)$$

(9) $$MAPE=\frac{1}{n}\sum_{i=1}^{n}\left|\frac{y_{i}-\hat{y}}{y_{i}}\right|\times 100$$

where n=10 denotes the number of test observations, $y_{i}$ is the value of test observations, and $\hat{y}$ is the optimum response value.

**Figure 8:**
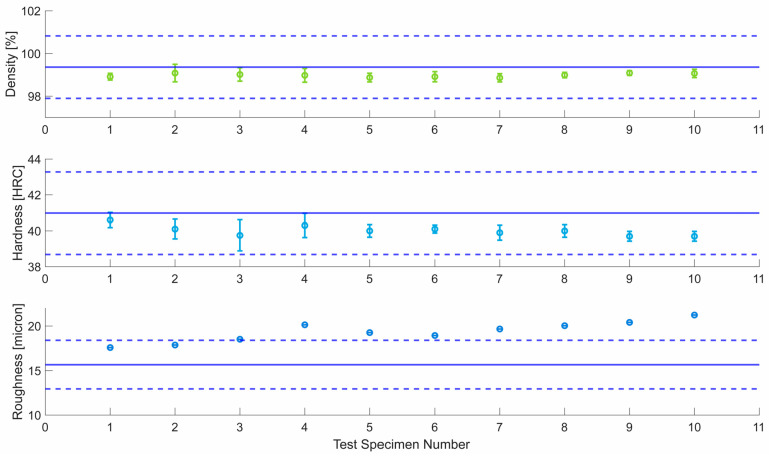
Density, hardness, and roughness of 10 specimens printed with optimized process parameters using Powder C. The solid horizontal line indicates the predicted optimal response values, while the dashed lines represent the 95% prediction intervals.

The results indicate that the density and hardness models achieve high prediction accuracies in the high 90s across all three PSDs. The roughness models exhibit lower but still acceptable accuracies, ranging from 76.6% to 83.5%. As previously noted, the roughness models have lower goodness-of-fit values, as evidenced by their reduced adjusted R-squared values. This discrepancy is likely due to noise and the lower repeatability of roughness data, which is suspected to be the primary contributing factor. Figure 8 displays the measured values for test specimens printed using Powder C as an example. The density and hardness data points are within the prediction intervals, consistently scattered around their average values, and show minimal fluctuations. In comparison, roughness values steadily increase, with several points falling outside the prediction interval. Notably, this occurs despite clustering the specimens closely in the build and taking extensive measures to eliminate potential sources of measurement error, as detailed in Section 2.5. These findings suggest that inconsistency in roughness is an inherent characteristic of the process for these PSDs, compounded by the limitations of areal roughness measurements discussed earlier.

Overall, the sequential methodology combining P-B and FFD has proven effective in efficiently optimizing the multi-variable print process for three PSDs and three responses with minimal experimental runs. Given the strong predictive accuracy of the models at this stage, further experimentation using CCD to incorporate quadratic terms into the regression appears unnecessary.

More importantly, the response values for offsize Powders B and C were experimentally confirmed to be on a par with those of the benchmark Powder A. The average density values as measured for the unseen test parts made from Powders A, B, and C are 99.5%, 98.7%, and 99%, respectively. The average hardness values are 37.9 HRC, 40.6 HRC, and 40 HRC for Powders A, B, and C, respectively. As previously mentioned, roughness is more sensitive to PSD size, as evidenced by the average roughness values of 12.4 µm, 14 µm, and 19.4 µm for Powders A, B, and C, respectively.

### 3.2. Scaling to Larger Geometries and Characterization

After completing the statistical modeling and optimization, larger functional parts were printed using optimized density parameters for further evaluation. The use of parameters presented in Table 5 in printing larger and taller parts resulted in the counter protruding beyond a certain height (~6 cm). To address this issue, the density regression models were re-optimized with the core energy limited to 200–250 W to prevent part failure caused by collusions between the upper layers and the recoater in the upper region of the parts. This highlights the sensitivity of process development in AM to sample size and geometry, as well as the challenges in transferring results between different geometries. Table 7 presents the optimal density parameters for three PSDs which happen to be identical.

#### 3.2.1. Porosity

**Figure 9:**
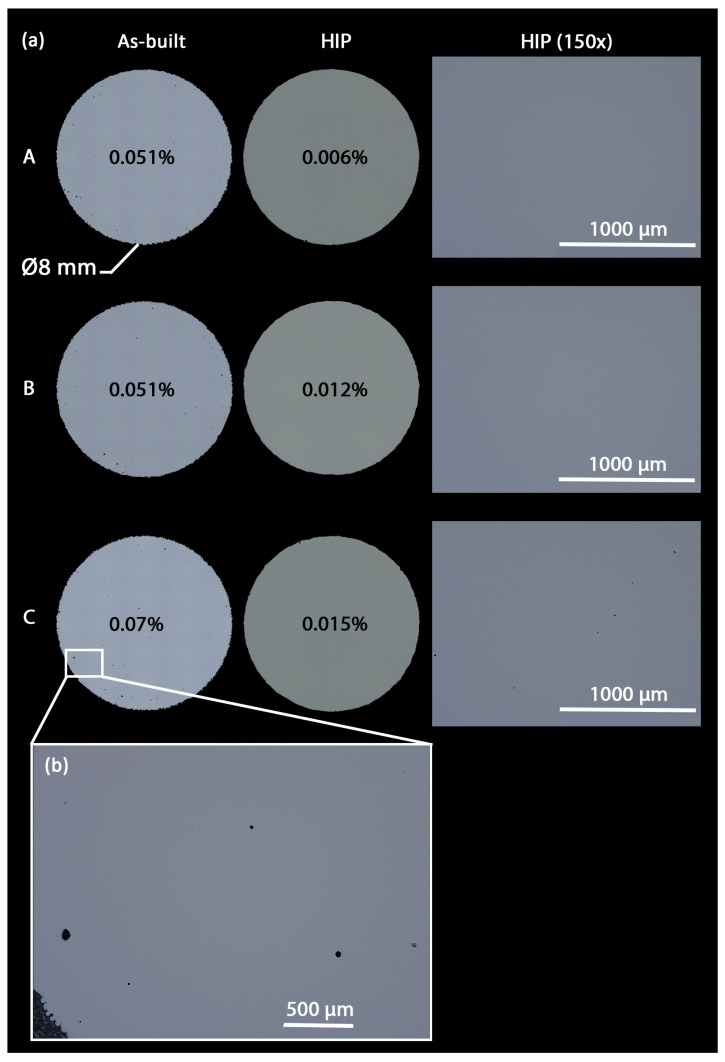
Micrograph of specimens fabricated at optimized density parameters with constrained core power, showing: (**a**) porosity of samples made using Powders A, B, and C in the as-built and HIP conditions (overall view and 150× magnified), and (**b**) a 100× magnified view of spherical pores in the sample produced with Powder C in the as-built condition. The porosity values are indicated on the respective images.

Cylindrical parts (Ø8 mm × H80 mm) were printed using each powder utilizing the process parameters presented in Table 7, with a subset subjected to HIP at 900 °C and 100 MPa for 2 h to evaluate the effect of HIP on porosity. The porosity of both the as-built and HIP parts was assessed through optical microscopy of polished surfaces, as shown in Figure 9.

The results demonstrate the effectiveness of the regression models in developing optimized parameter sets for new scenarios. The porosity levels in parts made from Powders A, B, and C are minimal and comparable (0.05–0.07%). However, parts produced using Powder C, with a larger PSD, exhibit slightly higher porosity. As shown in Figure 9b, the pores observed in the as-built condition are spherical, indicating that the optimized parameters successfully prevent lack of fusion defects by transitioning the material into a stable melting regime. The isolated spherical micro-voids show that under these processing conditions, residual porosity is governed by surface tension during rapid solidification rather than incomplete melting.

Although these pores are present throughout the parts, they appear to be more concentrated near the skin region. Since both the skin and core regions share similar energy densities (i.e., 2.75 J/mm^2^), this increased porosity near the skin could be attributed to the larger hatch distance used in the skin area. The HIP process appears to eliminate most of the pores, resulting in nearly fully dense parts with porosity levels below 0.02%.

**Figure 10:**
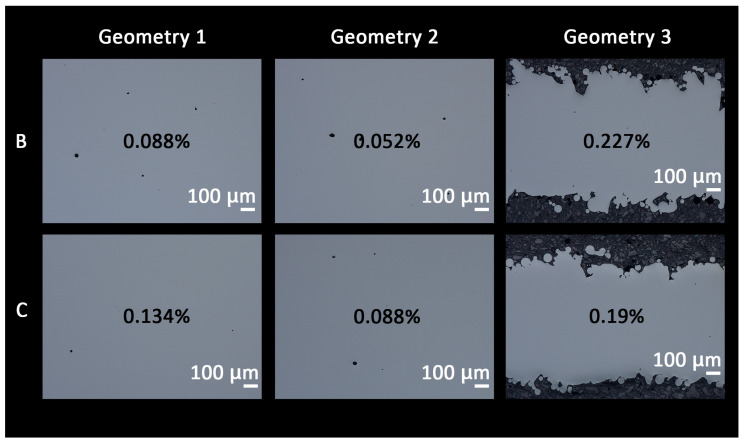
Micrographs with 150× magnification of geometries fabricated at optimized density parameters with constrained core power, using Powders B and C. The porosity values are indicated on the respective images.

Similarly, more complex geometries were printed using Powders B and C, as detailed in Section 2.3. Figure 10 presents the micrographs of sections of these parts. Due to confidentiality requirements, the complete parts cannot be shown. The measured porosity levels for these parts in the as-built condition ranged from 0.05% to 0.23%. Geometry 3, featuring a thin structure, exhibited higher porosity, likely due to the more pronounced influence of the skin area. These results support the use of offsize powders to produce approximately fully dense functional parts.

#### 3.2.2. Microstructure

**Figure 11:**
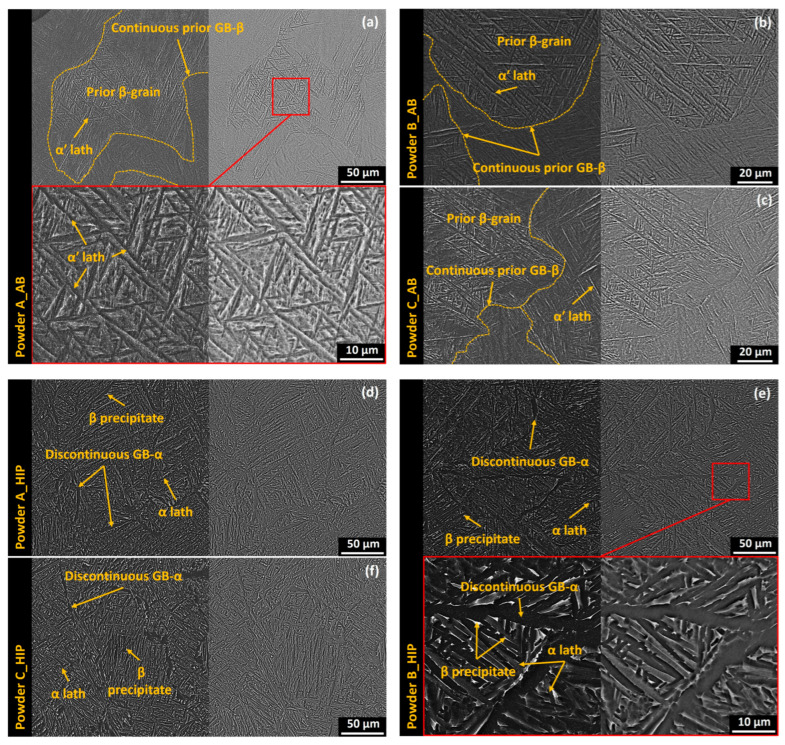
SEM images showing the microstructure of the as-built parts printed using Powders (**a**) A, (**b**) B, and (**c**) C. The microstructures after HIP treatment for the LPBF-fabricated parts using Powders (**d**) A, (**e**) B, and (**f**) C are also shown.

**Figure 12:**
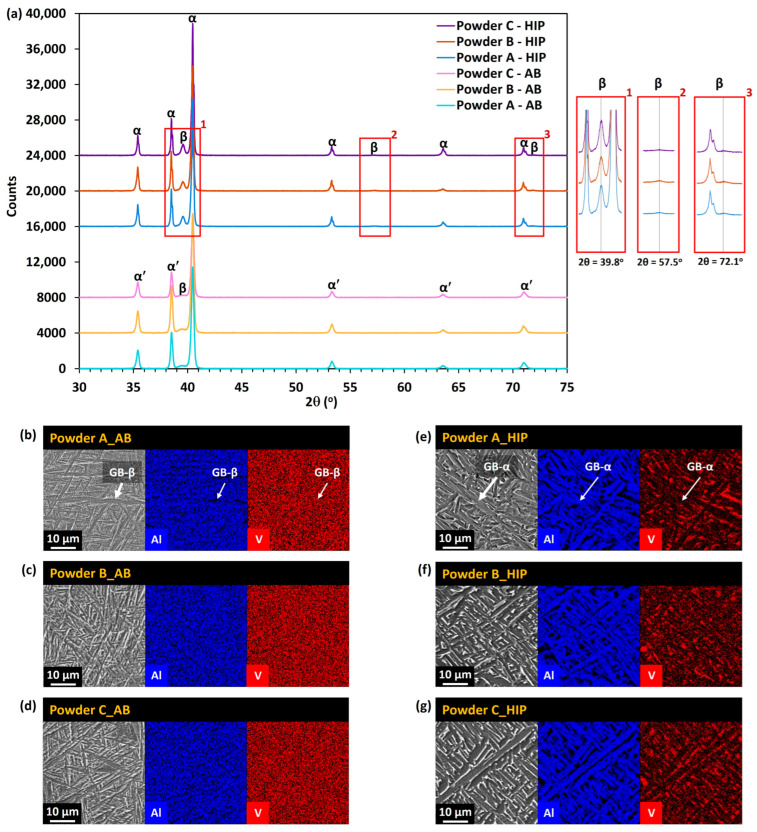
X-ray diffraction patterns of the as-built and HIP samples printed using Powders A, B, and C, with magnified XRD regions highlighted at selected 2θ positions (39.8°, 57.5° and 72.1°) (**a**). SEM-EDS maps for parts printed using Powders A, B, and C (**b**–**d**) in the as-built condition and (**e**–**g**) after post processed HIP condition, respectively.

To investigate the microstructure evolution in both the as-built condition and after HIP post-processing, cylindrical samples were prepared and analyzed using electron microscopy. Figure 11 presents the SEM images showing the formed phases and grain structures in both the as-built and HIP conditions, for specimens printed using Powders A, B, and C. Figure 12 illustrates the grain analysis of the same samples through inverse pole figure (IPF) maps and reconstructed grain maps obtained via the EBSD technique. Before discussing the results in detail, some critical facts about the microstructure of Ti-6Al-4V should be mentioned.

Ti-6Al-4V is an α+β titanium alloy, containing 6 wt.% aluminum (an α-stabilizer alloying element) and 4 wt.% vanadium (a β-stabilizer alloying element). The α and β phases are the stable phases of Ti-6Al-4V, with the α-phase having a hexagonal close-packed (HCP) crystal structure, and the β-phase a body-centered cubic (BCC) structure [62,63]. The beta transus temperature of this alloy is about 995 ± 15 °C, indicating that the β-phase is stable above this temperature, while the α-phase, enriched in aluminum, can form below it [64,65,66]. Since α-phase formation is diffusional transformation, it requires sufficient time and temperature. Therefore, rapid cooling from above the beta transus temperature or the melting point leads to the formation of a metastable α′-phase at room temperature. The martensitic α′-phase retains a significant amount of vanadium, and depletion of this alloying element requires several hours at elevated temperatures (below the beta transus) [67,68].

Figure 11a shows the SEM image of the etched cross-sectioned surface, revealing the microstructure of the part printed with Powder A, including prior β grains, continuous grain boundary β (GB-β), and α′ laths. As shown in this figure, the prior β grains are outlined by yellow dashed lines, which also indicate the grain boundary β (GB-β). The α′ laths are visible within the prior β grains, exhibiting a martensitic basketweave morphology. This martensitic microstructure is usually expected in the as-built condition of LPBF-made Ti-6Al-4V, as the solidification and cooling rates in LPBF of Ti alloys are extremely high [69,70]. Figure 11b,c presents the same microstructure for the as-built sample printed using Powders B and C, respectively.

**Figure 13:**
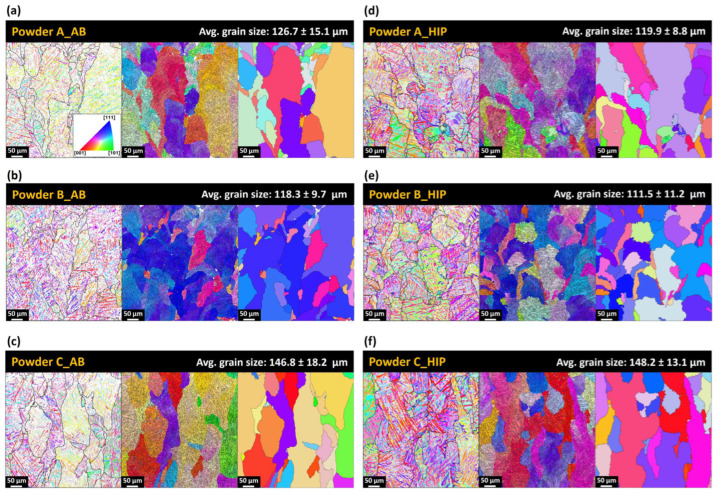
IPF-Z maps and reconstructed parent grain maps for parts printed using Powders A, B, and C in the as-built condition (**a**–**c**) and the HIP condition (**d**–**f**), respectively.

The HIP post-processing at 900 ± 25 °C and 100 ± 1.72 MPa for 2 h, used in this study to improve the density of the as-built samples, can also affect the microstructure in a manner similar to heat treatment. Heat treatment at any temperature below the beta transus (sub-transus), for a sufficient time (typically a few hours), promotes diffusional transformation and leads to the formation of more stable microstructures [70,71,72]. The microstructures of the HIP samples printed with Powders A, B, and C are shown in Figure 12d–f. These figures reveal similar microstructures across all three powders, characterized by β precipitates, discontinuous grain boundary α (GB-α), and α laths. To enable a more in-depth discussion of the identified grains and phases, the X-ray diffraction patterns (Figure 12a) and SEM-EDS maps of all conditions (Figure 13) will be used.

The XRD result plotted in Figure 12a shows that the as-built samples printed with Powders A, B, and C have similar diffraction patterns, indicating that the difference in PSD size does not notably affect the as-built microstructure. A similar result is observed for the HIP samples, with nearly identical XRD patterns obtained for parts printed with Powders A, B, and C. The remarkable difference between the as-built and HIP XRD patterns is the appearance of distinct β-phase peaks in the HIP samples, although a weak β-phase peak around 2θ ≈ 39.8° can also be seen in the as-built condition. Another difference is that the XRD patterns of the as-built samples exhibit the metastable α′-phase, whereas the HIP samples show peaks corresponding to the stable α-phase. The main cause of these two differences is attributed to the diffusional nature of the reactions responsible for the formation of the α and β phases.

Quantitative phase analysis was performed using the Rietveld refinement method to determine the retained β-phase fraction in the Ti-6Al-4V specimens. The results indicated that the retained β-phase fraction for all three powders increased from approximately 1.8 vol.% in the as-built condition to approximately 3.8 vol.% following HIP, corresponding to an increase of more than twofold.

As mentioned previously, in the microstructure of the as-built Ti-6Al-4V samples, the β-stabilizer V atoms do not have sufficient time to diffuse out of the α′-phase (laths) below β transus, due to supercooling during the solidification process and cooling, resulting in α′ laths that are supersaturated with V atoms. The sufficient time and temperature provided by the HIP post-processing facilitate the diffusional reaction α′ → α + β. As a result, V atoms are depleted from the α′ laths and diffuse into the surrounding regions, forming β precipitates, while new stable α laths, enriched with α-stabilizer Al atoms, are formed. The comparison of the SEM-EDS maps of the as-built and HIP samples in Figure 12b–d and Figure 12e–g, respectively, confirms the mentioned observations.

As the α′ laths are supersaturated with V atoms, there is no significant contrast in the Al and V EDS maps of the as-built samples in Figure 12b–d. However, in Figure 12e–g, it can be observed that the depletion of V from the α′ laths and its diffusion into the surrounding regions in the HIP samples causes a significant contrast, with Al-rich α laths and V-rich β precipitates sandwiched between the α laths. In the higher-magnification SEM image of the HIP sample (Powder B) in Figure 11e, the dark α laths and bright β precipitates are clearly visible. The formation of this β precipitate causes the indicated β peaks in the XRD pattern of HIP samples in Figure 12a.

In addition to the α′ → α + β phase transformation, the prior GB-β observed in the as-bult samples can transform to the more stable GB-α with less activation energy [73]. HIP post-processing facilitates the diffusion of V from the prior GB-β, leading to the formation of Al-rich GB-α. As shown in Figure 12b,e, the EDS maps illustrate the presence of GB-β in the as-built condition and GB-α in the HIP condition, respectively. In Figure 12b, the GB-β shows a slight lack of Al with no significant V contrast, whereas in Figure 12e, the GB-α appears Al-rich and V-lean. It should be noted that the greater thickness of GB-α and α laths, compared to GB-β and α′ laths observed in the microscopic images, and their influence on mechanical behavior will be discussed in future work.

Identification of prior β grain and grain size measurement is critical, but there is currently no simple experimental method available for revealing the prior β grains. In this study, grain statistics were obtained from microscopic EBSD scanning, using the parent grain reconstruction (PGR) method, to investigate the effects of different powder PSDs for Powders A, B, and C, as well as the effect of HIP post-processing on average grain size. Figure 13 presents the EBSD results in the form of IPF maps and reconstructed parent grain maps for samples printed with Powders A, B, and C, in both the as-built and HIP conditions. A columnar grain structure for all the powder PSDs and conditions is observed in this figure. The columnar microstructure is common in LPBF of Ti alloys due to the high thermal gradients and directional solidification along the build direction. This promotes epitaxial grain growth from layer to layer [58,74,75].

As shown in Figure 13a–c, the average grain sizes of the as-built samples printed with Powders A, B, and C are 126.7 ± 15.1 µm, 118.3 ± 9.7 µm, and 146.8 ± 18.2 µm, respectively. Considering the standard deviations, the differences in average grain size are negligible, indicating that the variation in powder PSDs does not significantly affect the as-built grain size. The average grain sizes of the HIP samples, shown in Figure 13d–f, are 119.9 ± 8.8 µm, 111.5 ± 11.2 µm, and 148.2 ± 13.1 µm, respectively, indicating that similar to the as-built condition, the grain size response to HIP post-processing is consistent across Powders A, B, and C. It is worth noting that the HIP post-processing at 900 ± 25 °C and 100 ± 1.72 MPa for 2 h did not affect the grain size. This is because the HIP temperature is less than the grain growth temperature of 950 °C, reported by Rani et al. [68].

#### 3.2.3. Dimensional Accuracy

For the dimensional accuracy analysis, samples produced using Powders B and C with target hole diameters ranging from 250 to 8000 µm, gap sizes from 50 to 1000 µm, and specifically designed wall thicknesses between 400 and 1000 µm were analyzed. Powder A with standard PSD was not included as the focus of this specific study is on the performance of offsize and wide powders in producing complex parts.

**Figure 14:**
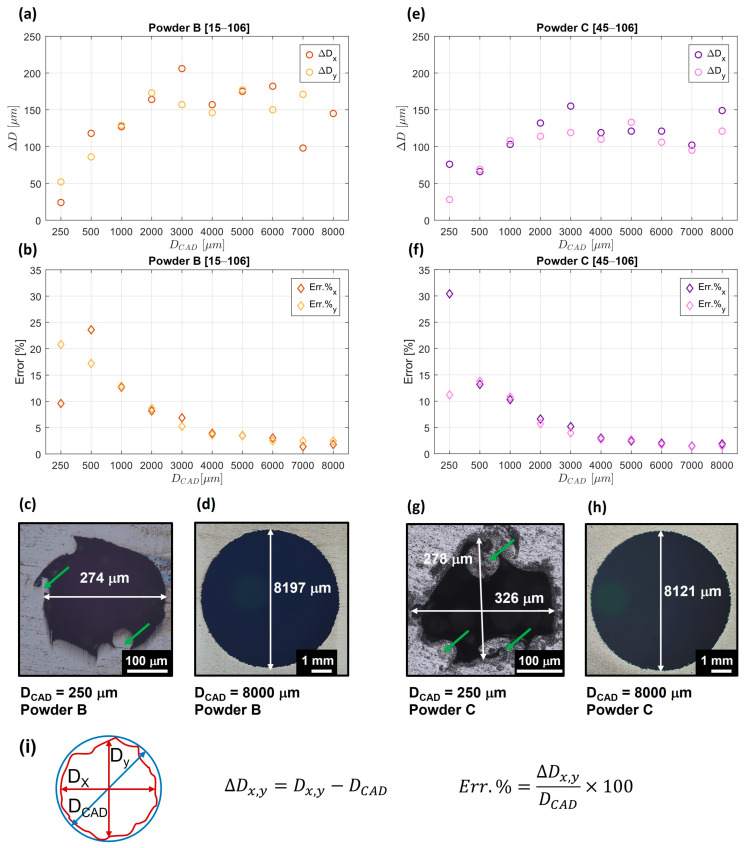
Evaluation of dimensional accuracy for drill holes: Diameter differences (ΔD_x,y_) for (**a**) Powder B and (**e**) Powder C; error percentages (Err.%) calculated for (**b**) Powder B and (**f**) Powder C; optical micrographs of the drill holes with the smallest and largest diameters for (**c**,**d**) Powder B and (**g**,**h**) Powder C; (**i**) schematic illustrating D_CAD_, D_x_, and D_y_, along with the equations for calculating ΔD_x,y_ and Err.%. Attached powders are marked with green arrows.

Figure 14a,e, which illustrate the difference (ΔD_x,y_) between the target diameter (D_CAD_) and the actual hole diameters in both the X and Y directions (D_x,y_), reveal that the deviations are more pronounced in samples produced using Powder B. There is no clear trend in the diameter differences in the X and Y directions for either powder. Instead, the variations appear random, indicating that the orientation of the sample on the build plate has no noticeable influence.

For better evaluation, the error percentage (Err.%) is plotted for each powder on Figure 14b,f, showing that for both Powders B and C, the values are in the positive range. Therefore, the measured hole diameters consistently appear to be larger than the target ones. For the samples with target hole diameters ranging from 2000 to 8000 µm, the error percentage is less than 10% in both the X and Y directions. Furthermore, for those with target diameters between 4000 and 8000 µm, the error drops to below 5%. The circles with D_CAD_ of 1000 μm show errors slightly above 10%, mainly falling within 11–12% range.

For both Powders B and C, the circles with target diameters of 250 μm and 500 μm tend to lose their roundness and become more irregular in shape contributing to the scattering of the results, while the circles with D_CAD_ of 1000–8000 μm maintain their intended roundness. The difference in shape fidelity becomes particularly evident in the smallest 250 µm diameter holes, where a few large, partially melted particles are observed attached to the edges, distorting the intended circular geometry. One possible explanation is increased heat accumulation and a larger heat-affected zone around the small circumference of these holes, which contributes to the partial melting of nearby powder particles and their subsequent attachment to the walls. Figure 14c,d,g,h demonstrate the overall shape fidelity of 250 μm and 8000 μm drill holes.

As a result, with the applied process parameters, samples with hole diameters ranging from 1000 to 8000 µm can be successfully printed using both Powders B and C, achieving high dimensional accuracy. However, to accurately produce parts with designated hole sizes smaller than 1000 µm, further optimization of the process parameters is required. This will be explored in future studies.

**Figure 15:**
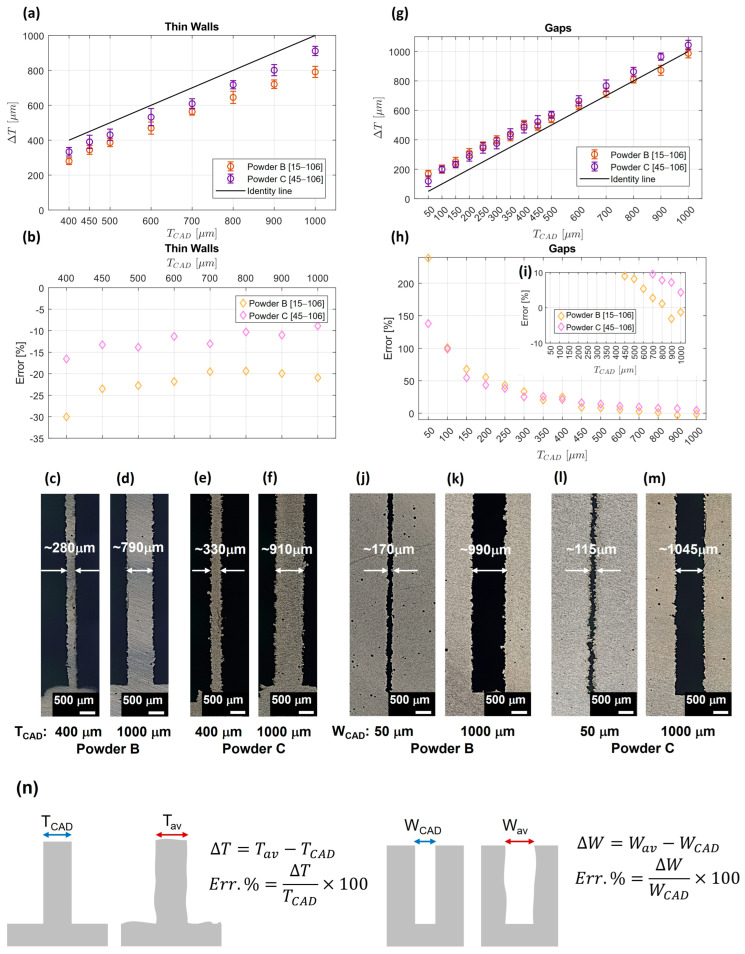
Evaluation of dimensional accuracy for thin walls and gaps: (**a**) Wall thickness differences (ΔT) for Powders B and C; (**b**) wall thickness error percentages (Err.%s) calculated for Powders B and C; optical micrographs of the walls with the thinnest and thickest widths for (**c**,**d**) Powder B and (**e**,**f**) Powder C; (**g**) gap width differences (ΔTs) for Powders B and C. (**h**,**i**) gap width error percentages (Err.%s) calculated for Powders B and C; optical micrographs of the smallest and largest gaps for (**j**,**k**) Powder B and (**l**,**m**) Powder C; (**n**) schematics illustrating T_CAD_, T_av_, W_CAD_, and W_av_, along with the equations for calculating ΔW, ΔT, and Err.%.

When printing thin walls with desired thicknesses ranging from 400 to 1000 µm, the use of both Powders B and C resulted in walls significantly thinner than the intended target (T_CAD_). As depicted in Figure 15a,b, the measured thickness for the walls produced from both powders falls below the identity line indicating consistent negative error margins. In comparison, the thin walls printed using Powder C are closer to the target values. For Powder C, the walls with target thicknesses of 800–1000 μm fall within an error range of approximately 10%. The walls printed using Powder B were significantly thinner than the original CAD design.

Analysis of the samples printed using Powders B and C with specifically designed gap widths (50–1000 μm), as shown in Figure 15g,h, revealed that gaps with the larger target widths are nearly aligned with the identity line for both powders. However, as the gap widths decrease, the measured values show greater deviation. Figure 15i, which highlights errors within the ±10% range, shows that the gaps with widths of 400–1000 µm for Powder B and 700–1000 µm for Powder C, respectively, fall within this error range. For both powders, further reduction in the target gap width results in a larger error. As shown in Figure 15j,l, the measured gaps (W_av_) are several times wider than the target of 50 μm, measuring approximately 170 μm for Powder B and 115 μm for Powder C. In contrast, Figure 15k,m show good alignment between the target and measured values for the larger gap width of 1000 μm.

A comparison of samples printed with similar gap widths and wall thicknesses shows that for Powder C, the wall thickness corresponding to a target value of 400 μm is approximately 335 μm. For the target gap of 400 μm, the measured width is approximately 485 μm. These results suggest that, for small features, the chosen parameters tend to produce walls that are thinner than the target and gaps that are wider than intended. These deviations become less pronounced when printing larger walls or gaps. A likely reason for obtaining thinner walls is that the selected process parameters (core, skin and contour) may not be adequate to fully melt the powders in the desired regions. A similar issue could be contributing to the irregularities seen in the thin gaps. Additionally, features printed with Powder B (Figure 15c,d,j,k) appear smoother compared to those printed with Powder C (Figure 15e,f,l,m). This could be due to the adherence of larger unmelted or partially melted powder particles in samples produced using Powder C, which has a PSD of 45–105 μm. In contrast, Powder B has a broader PSD of 15–105 μm, containing a greater portion of finer particles, which may result in better melting behavior and smoother surfaces.

To successfully print thin walls with precise dimensions, further optimization of the process parameters is necessary. The current parameter set appears to be suitable for printing gaps ranging from 450 to 1000 μm with Powder C and from 700 to 1000 μm with Powder B. To achieve higher print precision, particularly for fine features, additional adjustments to the skin and contour parameters will be pursued in the future.

## 4. Conclusions

The results of this study show that offsize and wide powders featuring a higher proportion of larger particles (100 to 160 μm) exhibit potential for use in the LPBF process, achieving a hardness and density similar to those of standard PSD (15–53 μm) under optimized processing conditions. Regardless of the powder PSD, the study demonstrated the viability of achieving nearly fully dense structures in the as-built condition, with most remaining pores sealed through HIP post-processing. Additionally, an average hardness ranging from 38 to 40.6 HRC was achieved.

Among the studied properties, only surface roughness exhibited statistically significant sensitivity to PSD after optimization. This is reasonable, given that in AM processes, roughness results from incomplete melting of particles on part surfaces, with larger particles in the offset powder bed leading to increased roughness. Despite this, the optimization process yielded roughness values below 16 μm for all powders.

The investigation of various aspects of the formed microstructure revealed that the printed samples with offsize powders (15–106 µm and 45–106 µm) exhibit a microstructure very similar to that of LPBF-made samples using the standard PSD (15–53 µm). Microstructure analysis using SEM, EDS, and XRD showed that HIP post-processing facilitated the formation of discontinuous GB-α, Al-rich α laths, and V-rich β precipitates equally across all three PSDs. Additionally, the reconstruction method used for EBSD analysis revealed that neither HIP post-processing nor variations in powder PSDs significantly affected the grain size in the LPBF-made samples.

The analysis of the dimensional accuracy of samples with specific hole diameters, gap widths, and wall thicknesses showed that features with target hole diameters ranging from 1000 to 8000 µm were successfully printed with an error margin of approximately 10% for both Powders B and C. Thin walls were generally measured to be thinner than intended, with only samples produced using Powder C—at target wall thicknesses of 800 to 1000 µm—falling within the 10% error range. For gap features, samples with target widths of 400 to 1000 µm (Powder B) and 700 to 1000 µm (Powder C) also met the acceptable deviation criteria. These findings indicate that while relatively large features can be produced with acceptable dimensional accuracy, finer features may require further optimization of process parameters for both Powders B and C.

In terms of optimization efforts, the multi-step statistical modeling approach presented in this work provided an efficient means of predictive analytics to study the impact of over 20 process parameters on three responses. This was achieved with only 208 data points (parts) for Powders A and B each, and 194 data points for Powder C. It is worth noting that the study replicated each experiment twice and included several center points in each design. Therefore, it is possible to achieve comparable results with half the current data points, although the robustness of the models might suffer. Studying three responses simultaneously imposed some strain on the approach, necessitating a lower-resolution FFD model (resolution V would have been preferred). Therefore, the approach is anticipated to perform even more effectively for a single response.

Additionally, the exploration of secondary print parameters within the contour–skin–core printing strategy, as well as the inclusion of other factors of this kind like support features and stripe/chess overlaps revealed the importance of some of these factors. The study also highlighted the need for mechanistic or numerical models explaining and leveraging the effect of these secondary parameters. As previously pointed out, a key hurdle in implementing DoE for multivariate modeling, particularly when focusing on empirical data alongside algorithmic optimizations, lies in comprehending the underlying physical explanations for specific parameters of significance. Similar to machine learning, some aspects of DoE can be considered black-box, especially in intricate higher-dimensional models featuring numerous significant interactions, highlighting the need for physical modeling alongside experimental efforts. The effect of additional parameters such as inert gas flow, upskin and downskin properties, shrinkage scales, beam shape, and beam offset can be investigated in future experiments to improve surface roughness and dimensional accuracy.

Future work will provide a comprehensive analysis of the optimized parts’ tensile and fatigue behavior, accompanied by fractography, microstructural, and compositional analyses under four conditions: as-built, stress relieved, annealed and HIP.

## Figures and Tables

**Table 1:** PSD and sphericity of the Ti-6Al-4V powders used in the study.

Powder Batch	D10 ± Std. Dev. [µm]	D50 ± Std. Dev. [µm]	D90 ± Std. Dev. [µm]	Sphericity ± Std. Dev.
A [15–53 µm]	19.53 ± 0.14	35.52 ± 0.18	51.29 ± 0.08	0.95 ± 0.00
B [15–106 µm]	25.51 ± 0.06	47.58 ± 0.42	87.21 ± 0.50	0.96 ± 0.00
C [45–106 µm]	49.0 ± 0.16	65.97 ± 0.78	92.65 ± 1.29	0.95 ± 0.00

**Table 2:** The chemical composition of the Grade 23 Ti-6Al-4V alloy utilized in this study aligns with the chemical requirements outlined in ASTM F3001.

Standard/Powder Batch	Elements [wt.%]								
	Aluminum (Al)	Vanadium (V)	Iron (Fe)	Carbon (C)	Oxygen (O)	Nitrogen (N)	Hydrogen (H)	Yttrium (Y)	Titanium (Ti)
ASTM F3001	5.5–6.5	3.5–4.5	0–0.25	0–0.08	0–0.13	0–0.05	0–0.012		Balance
A [15–53 µm]	6.41	4.01	0.19	0.01	0.09	0.02	0.001	<0.001	Balance
B [15–106 µm]	6.43	3.87	0.18	0.01	0.12	0.01	0.002	<0.001	Balance
C [45–106 µm]	6.39	3.98	0.17	0.02	0.07	0.01	0.004	<0.001	Balance

**Table 3:** Optimization steps for each powder and their corresponding label.

Step	Design Label		
	Powder A	Powder B	Powder C
(1) Plackett–Burman (P-B)	A1	B1	C1
(2) Fractional Factorial Design (FFD)	A2	B2	C2
(3) Optimization (DFM) and Validation	A3	B3	C3

**Table 4:** The most significant process parameters (lowest *p*-values) for each powder–response combination identified at the P-B analysis step. For the complete list of significant parameters, refer to the Supplementary Information.

		Density			Hardness			Roughness		
Category	Parameter	A1	B1	C1	A1	B1	C1	A1	B1	C1
Pre-Exposure	Contour Speed [mm/s]									
	Contour Power [W]									
Post-Exposure	Contour Speed [mm/s]									
	Contour Power [W]									
Skin Exposure	Energy Density [J/mm^2^]				x					x
	Power [W]				x	x				x
	Hatch Distance [mm]									
	Stripe Width [mm]									
	Stripe Overlap [mm]									
	Skin Thickness X/Y [mm]							x	x	
	Skin Thickness Z [mm]				x	x		x		
Core Exposure	Energy Density [J/mm^2^]	x	x							
	Power [W]	x	x	x	x	x	x			
	Hatch Distance [mm]								x	
	Stripe/Square Width [mm]							x	x	
	Stripe/Square Overlap [mm]								x	
Support Structure	Support-Area Spacing [mm]									
	Support Height [mm]		x					x		
Other	Layer Thickness [mm]			x			x	x	x	x
	Angle [deg]			N/A			N/A			N/A
	Print Location [mm]	x		N/A			N/A			N/A
	Geometry	x	x		x					
	Hatching Method									

**Table 5:** The optimized values of significant process parameters, along with the optimum response values and 95% prediction intervals for density, hardness, and roughness, determined using the DFM for Powders A, B, and C. Parameters marked with (*) are identified as significant based on the ANOVA for designs A2, B2, and C2. Parameters marked with (†) are not inherently significant but are retained in the regression model to comply with the hierarchy rule. No value has been provided for parameters deemed not significant. For geometry, ‘Cy’ indicates cylinder and ‘C’ indicates cuboid. For hatching method, ‘S’ denotes stripe and ‘C’ denotes chess.

Property		Density [%]			Hardness [HRC]			Roughness [µm]		
	Parameter	A	B	C	A	B	C	A	B	C
Powder										
Pre-Contour Speed [mm/s]										
Pre-Contour Power [W]				60 †			60 †			60 *
Post-Contour Speed [mm/s]										
Post-Contour Power [W]		160 †	0 *	10 †	160 †	0 †	10 *	160 †	160 *	10 *
Skin-Energy Density [J/mm^2^]		1.5 *	1.5 *	1.5 †	1.5 *	1.5 *	1.5 *	1.5 *	4 *	4 *
Skin-Power [W]				150 *			150 *			150 *
Skin-Hatch Distance [mm]		0.08 *			0.12 *			0.12 *		
Skin-Stripe Width [mm]										
Skin-Stripe Overlap [mm]		0.01 †	0.15 *			0.01 *		0.15 *	0.01 †	0.01 †
Skin-Thickness XY [mm]		1 *	0.2 *		1 *	1 *		1 *		
Skin-Thickness Z [mm]		0.2 †	0.2 †		0.2 *	1 †	0.2 †	0.2 *	1 †	0.2 †
Core-Energy Density [J/mm^2^]		1.5 *	4 *	4 *	1.5 *	4 *	4 *	1.5 *	4 *	4 †
Core-Power [W]		150 *	350 *	350 *	150 *	350 *	350 *	350 †	150 *	350 †
Core-Hatch Distance [mm]			0.1 *	0.1 *		0.1 †	0.08 †		0.08 †	0.08 †
Core-Stripe/Square Width [mm]		2 †	2 *		5 †	5 †		2 †		
Core-Stripe/Square Overlap [mm]			0.15 †			0.01 †				
Support Area Spacing [mm]				0.8 †						0.6 †
Support Height [mm]		8 *	0 *	8 †	8 †	0 †	8 †	0 *		2 †
Layer Thickness [mm]		0.02 *	0.02 *	0.02 *	0.02 *	0.02 *	0.02 *	0.06 †	0.02 †	0.02 *
Angle [deg]										
Print Location [mm]		A †	B *		A †	A *		B *	A *	
Geometry		Cy †	C *		C *	C *				
Hatching Method		S *	C †	C *	S †	C †	S *	S *	S †	C *
Optimum Response Value		100	99.99	99.36	40.99	41	40.98	9.5	11.72	15.67
Prediction Intervals		−0.61	−0.69	−1.46	±2.63	±2.63	±2.3	±2.34	±2.52	±2.27

**Table 6:** Summary of test observation averages and model prediction accuracies. The values in this table are rounded to one decimal point for improved readability.

	Density [%]			Hardness [HRC]			Roughness [µm]		
	A	B	C	A	B	C	A	B	C
Optimum Response Value	100	100	99.4	41	41	41	9.5	11.7	15.7
Average of Test Observations	99.5	98.7	99	37.9	40.6	40	12.4	14	19.4
RMSE	0.5	1.3	0.4	3.09	0.5	1	1.7	2.4	3.8
Prediction Accuracy	99.5%	98.7%	99.6%	91.9%	98.9%	97.6%	76.6%	83.5%	81.2%

**Table 7:** The optimized values of significant process parameters for density after limiting the core power to 200–250 W for Powders A, B, and C. Parameters marked with (*) are identified as significant based on the ANOVA of designs A2, B2, and C2. Parameters marked with (†) are not inherently significant but are retained in the regression model to comply with the hierarchy rule. No value has been provided for parameters deemed not significant. For geometry, ‘Cy’ indicates cylinder and ‘C’ indicates cuboid. For hatching method, ‘S’ denotes stripe and ‘C’ denotes chess.

Property		Density [%]		
	Powder	A	B	C
Parameter				
Pre-Contour Speed [mm/s]				
Pre-Contour Power [W]				30 †
Post-Contour Speed [mm/s]				
Post-Contour Power [W]		80 †	80 *	80 †
Skin-Energy Density [J/mm^2^]		2.75 *	2.75 *	2.75 †
Skin-Power [W]				250 *
Skin-Hatch Distance [mm]		0.1 *		
Skin-Stripe Width [mm]				
Skin-Stripe Overlap [mm]		0.08 †	0.08 *	
Skin-Thickness XY [mm]		0.6 *	0.6 *	
Skin-Thickness Z [mm]		0.6 †	0.6 †	
Core-Energy Density [J/mm^2^]		2.75 *	2.75 *	2.75 *
Core-Power [W]		250 *	250 *	250 *
Core-Hatch Distance [mm]			0.09 *	0.09 *
Core-Stripe/Square Width [mm]		3.5 †	3.5 *	
Core-Stripe/Square Overlap [mm]			0.08 †	
Support Area Spacing [mm]				0.6 †
Support Height [mm]		4 *	4 *	4 †
Layer Thickness [mm]		0.04 *	0.04 *	0.04 *
Angle [deg]				
Print Location [mm]		B †	B *	
Geometry		Cy †	Cy *	
Hatching Method		S *	S †	S *

## Data Availability

The original contributions presented in this study are included in the article/Supplementary Material. Further inquiries can be directed to the corresponding authors.

## Supplementary Materials

The following supporting information can be downloaded at https://www.mdpi.com/article/10.3390/ma19143049/s1.

## Abbreviations

The following abbreviations are used in this manuscript:

AM	Additive manufacturing
LPBF	Laser powder bed fusion
PSD	Particle size distribution
MMPDS	Metallic materials properties development and standardization
FD	Factorial designs
DoE	Design of experiment
FFD	Fractional factorial design
P-B	Plackett–Burman
DFM	Desirability function method
SEM	Scanning electron microscopy
EDS	Energy-dispersive spectroscopy
EBSD	Electron backscatter diffraction
XRD	X-ray diffraction
EDM	Electrical discharge machining
ANOVA	Analysis of variance
HRC	Hardness Rockwell C
VH	Vickers hardness
RMSE	Root mean square error
CCD	Central composite design
HCP	Hexagonal close-packed
BCC	Body-centered cubic
GB	Grain boundary
HIP	Hot isostatic pressing
CAD	Computer-aided design

## References

[1] Bromberger J., Ilg J., Miranda A.M. (2022). The Mainstreaming of Additive Manufacturing.

[2] Spierings A.B., Levy G. (2009). Comparison of density of stainless steel 316L parts produced with selective laser melting using different powder grades. International Solid Freeform Fabrication Symposium 2009.

[3] Vock S., Klöden B., Kirchner A., Weißgärber T., Kieback B. (2019). Powders for powder bed fusion: A review. Prog. Addit. Manuf..

[4] Liu B., Wildman R., Tuck C., Ashcroft I., Hague R. (2011). Investigation the effect of particle size distribution on processing parameters optimisation in selective laser melting process. International Solid Freeform Fabrication Symposium 2011.

[5] Yuasa K., Tagami M., Yonehara M., Ikeshoji T.T., Takeshita K., Aoki H., Kyogoku H. (2021). Influences of powder characteristics and recoating conditions on surface morphology of powder bed in metal additive manufacturing. Int. J. Adv. Manuf. Technol..

[6] Clayton J., Millington-Smith D., Armstrong B. (2015). The application of powder rheology in additive manufacturing. JOM.

[7] Baesso I., Karl D., Spitzer A., Gurlo A., Günster J., Zocca A. (2021). Characterization of powder flow behavior for additive manufacturing. Addit. Manuf..

[8] Stegman B.T., Lopez J., Jarosinski W., Wang H., Zhang X. (2023). The influence of powder particle size distributions on mechanical properties of alloy 718 by laser powder bed fusion. Metals.

[9] Strondl A., Lyckfeldt O., Brodin H., Ackelid U. (2015). Characterization and Control of Powder Properties for Additive Manufacturing. JOM.

[10] Habibnejad-Korayem M., Lalh M., Schunk C., Zou Y. (2023). Offsize particle size utilization for laser powder bed fusion processing of plasma atomized Ti-6Al-4V powders: Impacts on part properties and powder safety. J. Manuf. Process..

[11] Kim M.S., Kim O., Song Y., Ko C., Kim J., Habibnejad-Korayem M., Kim J.H. (2024). Cost-Effective Laser Powder Bed Fusion of Ti-6Al-4V Grade 5: The Effect of Expanding Powder Size Distribution on Mechanical Performance. Materials.

[12] Haferkamp L. (2022). Effect of the Particle Size Distribution and Morphology on Powder Processability in Laser Powder Bed Fusion. Doctoral Dissertation.

[13] Spierings A.B., Herres N., Levy G. (2011). Influence of the particle size distribution on surface quality and mechanical properties in AM steel parts. Rapid Prototyp. J..

[14] Simchi A. (2004). The role of particle size on the laser sintering of iron powder. Metall. Mater. Trans. B Process Metall. Mater. Process. Sci..

[15] Abd-Elghany K., Bourell D.L. (2012). Property evaluation of 304L stainless steel fabricated by selective laser melting. Rapid Prototyp. J..

[16] Ziri S., Hor A., Mabru C. (2022). Combined effect of powder properties and process parameters on the density of 316L stainless steel obtained by laser powder bed fusion. Int. J. Adv. Manuf. Technol..

[17] Soliman H.A., Yakout M., Elbestawi M. (2022). Processes, Laser powder bed fusion of titanium aluminides using sequential thermal scanning strategy. J. Manuf. Process..

[18] Rauniyar S., Shrestha S., Chou K. An Investigation Into Multi-Track Deposition in Laser Powder-Bed Fusion: Transient Regions Analysis and Scan Length Effects. Proceedings of the ASME 2022 17th International Manufacturing Science and Engineering Conference.

[19] Babu J.J., Mehrpouya M., Pijper T.C., Willemsen G., Vaneker T. (2022). An experimental study of downfacing surfaces in selective laser melting. Adv. Eng. Mater..

[20] Meylan B., Calderon I., Wasmer K. (2022). Optimization of process parameters for the laser polishing of hardened tool steel. Materials.

[21] Ladani L., Razmi J., Sadeghilaridjani M. (2022). Fabrication of Cu-CNT composite and Cu using laser powder bed fusion additive manufacturing. Powders.

[22] Elkaseer A., Charles A., Schneider S., Scholz S.G. (2022). Part tailoring in metal-additive manufacturing: A step towards functionally graded customized stainless-steel components using laser powder bed fusion. Appl. Sci..

[23] Vele F., Ackermann M., Bittner V., Šafka J. (2021). Influence of selective laser melting technology process parameters on porosity and hardness of aisi h13 tool steel: Statistical approach. Materials.

[24] Yakout M., Elbestawi M.A., Veldhuis S.C. (2020). A study of the relationship between thermal expansion and residual stresses in selective laser melting of Ti-6Al-4V. J. Manuf. Process..

[25] Galetto M., Genta G., Maculotti G., Verna E. (2020). Defect Probability Estimation for Hardness-Optimised Parts by Selective Laser Melting. Int. J. Precis. Eng. Manuf..

[26] Dzukey G.A., Yang K., Wang Q., Zhuang B., Hou W. (2020). Porosity, Hardness, Friction and Wear Performance Analysis of H13 SLM-Formed Samples. J. Mater. Eng. Perform..

[27] Bosio F., Aversa A., Lorusso M., Marola S., Gianoglio D., Battezzati L., Lombardi M. (2019). A time-saving and cost-effective method to process alloys by Laser Powder Bed Fusion. Mater. Des..

[28] Aboutaleb A.M., Mahtabi M.J., Tschopp M.A., Bian L. (2019). Multi-objective accelerated process optimization of mechanical properties in laser-based additive manufacturing: Case study on Selective Laser Melting (SLM) Ti-6Al-4V. J. Manuf. Process..

[29] Sing S.L., Wiria F.E., Yeong W.Y. (2018). Selective laser melting of lattice structures: A statistical approach to manufacturability and mechanical behavior. Robot. Comput.-Integr. Manuf..

[30] Li Z., Kucukkoc I., Zhang D.Z., Liu F. (2018). Optimising the process parameters of selective laser melting for the fabrication of Ti6Al4V alloy. Rapid Prototyp. J..

[31] Pupo Y., Monroy K.P., Ciurana J. (2015). Influence of process parameters on surface quality of CoCrMo produced by selective laser melting. Int. J. Adv. Manuf. Technol..

[32] Pyka G., Kerckhofs G., Papantoniou I., Speirs M., Schrooten J., Wevers M. (2013). Surface roughness and morphology customization of additive manufactured open porous Ti6Al4V structures. Materials.

[33] Monroy K., Delgado J., Ciurana J. (2013). Study of the pore formation on CoCrMo alloys by selective laser melting manufacturing process. Procedia Eng..

[34] Lu C., Shi J. (2022). Relative density and surface roughness prediction for Inconel 718 by selective laser melting: Central composite design and multi-objective optimization. Int. J. Adv. Manuf. Technol..

[35] Oyesola M., Mpofu K., Mathe N., Fatoba S., Hoosain S., Daniyan I. (2021). Daniyan, Optimization of selective laser melting process parameters for surface quality performance of the fabricated Ti6Al4V. Int. J. Adv. Manuf. Technol..

[36] Khademzadeh S., Gennari C., Zanovello A., Franceschi M., Campagnolo A., Brunelli K. (2022). Development of micro laser powder bed fusion for additive manufacturing of Inconel 718. Materials.

[37] De Leon Nope G.V., Perez-Andrade L.I., Corona-Castuera J., Espinosa-Arbelaez D.G., Muñoz-Saldaña J., Alvarado-Orozco J.M. (2021). Study of volumetric energy density limitations on the IN718 mesostructure and microstructure in laser powder bed fusion process. J. Manuf. Process..

[38] Zhuang J.R., Lee Y.T., Hsieh W.H., Yang A.S. (2018). Determination of melt pool dimensions using DOE-FEM and RSM with process window during SLM of Ti6Al4V powder. Opt. Laser Technol..

[39] Ozsoy A., Yasa E., Keles M., Tureyen E.B. (2021). Pulsed-mode Selective Laser Melting of 17-4 PH stainless steel: Effect of laser parameters on density and mechanical properties. J. Manuf. Process..

[40] Alfaify A.Y., Hughes J., Ridgway K. (2018). Critical evaluation of the pulsed selective laser melting process when fabricating Ti64 parts using a range of particle size distributions. Addit. Manuf..

[41] Kuo C., Su C., Chiang A. (2017). Parametric optimization of density and dimensions in three-dimensional printing of Ti-6Al-4V powders on titanium plates using selective laser melting. Int. J. Precis. Eng. Manuf..

[42] Chia H.Y., Wu J., Wang X., Yan W. (2022). Process parameter optimization of metal additive manufacturing: A review and outlook. J. Mater. Inform..

[43] Sutton A.T., Kriewall C.S., Leu M.C., Newkirk J.W. (2017). Powder characterisation techniques and effects of powder characteristics on part properties in powder-bed fusion processes. Virtual Phys. Prototyp..

[44] Fayazfar H., Salarian M., Rogalsky A., Sarker D., Russo P., Paserin V., Toyserkani E. (2018). A critical review of powder-based additive manufacturing of ferrous alloys: Process parameters, microstructure and mechanical properties. Mater. Des..

[45] Sabelkin V.P., Cobb G.R., Doane B.M., Kemnitz R.A., O’Hara R.P. (2020). Torsional behavior of additively manufactured nickel alloy 718 under monotonic loading and low cycle fatigue. Mater. Today Commun..

[46] Sabelkin V.P., Cobb G.R., Shelton T.E., Hartsfield M.N., Newell D.J., O’Hara R.P., Kemnitz R.A. (2019). Mitigation of anisotropic fatigue in nickel alloy 718 manufactured via selective laser melting. Mater. Des..

[47] Sutton B., Herderick E., Thodla R., Ahlfors M., Ramirez A. (2019). Heat Treatment of Alloy 718 Made by Additive Manufacturing for Oil and Gas Applications. JOM.

[48] Plackett R.L., Burman J.P. (1946). The design of optimum multifactorial experiments. Biometrika.

[49] Mahmoodkhani Y., Ali U., Liravi F., Esmaeilizadeh R., Marzbanrad E., Toyserkani E., Bonakdar A. (2018). Determination of the most contributing laser powder bed fusion process parameters on the surface roughness quality of Hastelloy X components. Glob. Power Propuls. Soc..

[50] Bonakdar A., Liravi F., Toyserkani E., Ali U., Chenouri S.E., Mahmoodkhani Y. (2025). Method and System for Optimzing Process Parameters in an Additive Manufacturing Process.

[51] (2022). Geometrical Product Specification (GPS)—Surface Texture: Areal—Calibration, Adjustment and Verification of Topography Measuring Instruments.

[52] (2021). Geometrical Product Specifications (GPS)—Surface Texture: Areal—Part 2: Terms, Definitions and Surface Texture Parameters.

[53] Niessen F., Nyyssönen T., Gazder A.A., Hielscher R. (2022). Parent grain reconstruction from partially or fully transformed microstructures in MTEX. J. Appl. Crystallogr..

[54] Schneider C.A., Rasband W.S., Eliceiri K.W. (2012). NIH Image to ImageJ: 25 years of image analysis. Nat. Methods.

[55] Montgomery D.C., Borror C.M., Stanley J.D. (1997). Some cautions in the use of plackett-burman designs. Qual. Eng..

[56] Wilson-Heid A.E., Wang Z., McCornac B., Beese A.M. (2017). Quantitative relationship between anisotropic strain to failure and grain morphology in additively manufactured Ti-6Al-4V. Mater. Sci. Eng. A.

[57] Campanelli S.L., Contuzzi N., Ludovico A.D., Caiazzo F., Cardaropoli F., Sergi V. (2014). Manufacturing and characterization of Ti6Al4V lattice components manufactured by selective laser melting. Materials.

[58] Cepeda-Jiménez C.M., Potenza F., Magalini E., Luchin V., Molinari A., Pérez-Prado M.T. (2020). Effect of energy density on the microstructure and texture evolution of Ti-6Al-4V manufactured by laser powder bed fusion. Mater. Charact..

[59] E140 Standard Hardness Conversion Tables for Metals Relationship Among Brinell Hardness, Vickers Hardness, Rockwell Hardness, Superficial Hardness, Knoop Hardness, Scleroscope Hardness, and Leeb Hardness. https://www.astm.org/e0140-12br19e01.html.

[60] Blau P.J., Erdman D.L., Ohriner E., Jolly B.C. (2011). High-temperature galling characteristics of Ti-6AL-4V with and without surface treatments. Tribol. Trans..

[61] Hickey C.F. (1961). Tensile Strength-Hardness Correlation for Titanium Alloys.

[62] Leyens C., Peters M. (2003). Titanium and Titanium Alloys: Fundamentals and Applications.

[63] Xie Z., Dai Y., Ou X., Ni S., Song M. (2020). Effects of selective laser melting build orientations on the microstructure and tensile performance of Ti–6Al–4V alloy. Mater. Sci. Eng. A.

[64] Villa M., Brooks J.W., Turner R.P., Wang H., Boitout F., Ward R.M. (2019). Microstructural modeling of the α+ β phase in Ti-6Al-4V: A diffusion-based approach. Metall. Mater. Trans. B.

[65] Zhang M., Ng C.H., Dehghan-Manshadi A., Hall C., Bermingham M.J., Dargusch M.S. (2023). Towards isotropic behaviour in Ti–6Al–4V fabricated with laser powder bed fusion and super transus hot isostatic pressing. Mater. Sci. Eng. A.

[66] Khorasani A.M., Gibson I., Ghasemi A., Ghaderi A. (2020). Modelling of laser powder bed fusion process and analysing the effective parameters on surface characteristics of Ti-6Al-4V. Int. J. Mech. Sci..

[67] Pathania A., Subramaniyan A.K., Nagesha B.K. (2022). Influence of post-heat treatments on microstructural and mechanical properties of LPBF-processed Ti6Al4V alloy. Prog. Addit. Manuf..

[68] Rani S.U., Kesavan D., Kamaraj M. (2023). Evaluation of influence of microstructural features of LPBF Ti-6Al-4 V on mechanical properties for an optimal strength and ductility. J. Alloys Compd..

[69] Hasanabadi M., Shahabad S.I., Keshavarzkermani A., Eybel R., Gerlich A., Toyserkani E. (2024). A numerical modelling for laser Powder-bed fusion of Ti-alloy with a hybrid heat Source: An investigation on solidification and microstructure formation. Opt. Laser Technol..

[70] Jamhari F.I., Foudzi F.M., Buhairi M.A., Sulong A.B., Radzuan N.A.M., Muhamad N., Tan K.S. (2023). Influence of heat treatment parameters on microstructure and mechanical performance of titanium alloy in LPBF: A brief review. J. Mater. Res. Technol..

[71] Hasanabadi M., Keshavarzkermani A., Azizi N., Asgari H., Gerlich A., Toyserkani E. (2024). Systematic investigation into laser powder bed fusion of Ti-5553 through single-track and multi-layer studies for tailored manufacturing solutions. J. Mater. Res. Technol..

[72] Cardon A., Mareau C., Ayed Y., Van Der Veen S., Giraud E., Dal Santo P. (2021). Heat treatment simulation of Ti-6Al-4V parts produced by selective laser melting. Addit. Manuf..

[73] Liu J., Zhang K., Liu J., Wang H., Yang Y., Yan L., Huang A. (2023). Investigation of fatigue behavior of laser powder bed fusion Ti-6Al-4V: Roles of heat treatment and microstructure. Int. J. Fatigue.

[74] Hasanabadi M., Keshavarzkermai A., Asgari H., Azizi N., Gerlich A., Toyserkani E. (2023). In-situ microstructure control by laser post-exposure treatment during laser powder-bed fusion. Addit. Manuf. Lett..

[75] Hasanabadi M., Asgari H., Azizi N., Aghajani H., Minasyan T., Toyserkani E. (2025). Towards sustainable additive manufacturing: Enhanced productivity via numerical-experimental melt pool engineering in laser powder bed fusion of Ti-alloy. J. Mater. Res. Technol..

