# Supplementary material for: Laser Powder Bed Fusion Processing of Ti-6Al-4V Powders with Offsize and Wide Particle Size Distributions—Process Optimization
Source: Materials (Basel). 2026 Jul 15;19(14):3049. doi: 10.3390/ma19143049 (PMC13413037; doi:10.3390/ma19143049)
Supplement: Supplementary file 1 [file materials-19-03049-s001.zip › materials-4398833-supplementary.pdf]

Supplementary Information

1 Design A1

In designs A1 and B1, 23 process parameters were investigated, comprising 21 continuous and 2 categorical variables. Consequently, 24 runs were conducted to evaluate the effects of the main parameters. To enhance the robustness and validity of the designs and reduce experimental error, two replicates of the designs and 8 center point runs were included, increasing the total number of runs for each design to 56. Table S1 provides details on the process parameters studied, along with their respective lower and upper limits.

Table S1. Categories, types, and levels of process parameters for Designs A1 and B1.

Category	No.	Code	Parameter	Type	Low (-1)	Mid (0)	High (+1)
**Pre-exposure**	1	A	Contour Speed [mm/s]	Continuous	700	1100	1500
	2	B	Contour Power [W]	Continuous	0	30	60
**Post-exposure**	3	C	Contour Speed [mm/s]	Continuous	700	1100	1500
	4	D	Contour Power [W]	Continuous	0	80	160
**Skin-exposure**	5	E	Energy Density [J/mm^2]	Continuous	1.5	2.75	4
	6	F	Power [W]	Continuous	150	250	350
	7	G	Hatch Distance [mm]	Continuous	0.08	0.1	0.12
	8	H	Stripe Width [mm]	Continuous	2	3.5	5
	9	J	Stripe Overlap [mm]	Continuous	0.01	0.08	0.15
	10	K	Skin Thickness X/Y [mm]	Continuous	0.2	0.6	1
	11	L	Skin Thickness Z [mm]	Continuous	0.2	0.6	1
**Core-exposure**	12	M	Energy Density [J/mm^2]	Continuous	1.5	2.75	4
	13	N	Power [W]	Continuous	150	250	350
	14	O	Hatch Distance [mm]	Continuous	0.08	0.09	0.1
	15	P	Stripe/Square Width [mm]	Continuous	2	3.5	5
	16	Q	Stripe/Square Overlap [mm]	Continuous	0.01	0.08	0.15
**Support Structure**	17	R	Support Area Spacing [mm]	Continuous	0.6	0.7	0.8
	18	S	Support Height [mm]	Continuous	0	4	8
**Other**	19	T	Layer Thickness [mm]	Continuous	0.02	0.04	0.06
	20	U	Angle [deg]	Continuous	0	45	90
	21	V	Print Location [mm]	Continuous	30	60	90
	22	W	Geometry	Categorical	Cube	N/A	Cylinder
	23	X	Hatching Method	Categorical	Stripe	N/A	Chess

Table S2 displays the coded structure of the P-B design used in A1 and B1.

Table S2. P-B Designs A1 and B1, including only the first replicate for brevity. The second replicate and runs at center points are excluded. The second replicate shares the exact alias as the first one. For center points, all continuous parameters are fixed at 0, and the categorical ones alternate between + and -. For the parameter W (Geometry), 'Cy' denotes Cylinder, and 'C' denotes Cube. For the parameter X (Hatching Method), 'S' denotes Stripe and 'C' denotes Chess.

Part Code	A	B	C	D	E	F	G	H	J	K	L	M	N	O	P	Q	R	S	T	U	V	W	X
TA1-01 / TB1-01	+	-	-	-	-	+	-	+	-	-	+	+	+	-	+	+	-	+	-	+	+	Cy	C
TA1-02 / TB1-02	+	+	-	-	-	-	+	-	+	-	-	+	+	-	-	+	+	-	+	-	+	Cy	C

TA1-03 / TB1-03	+	+	+	-	-	-	-	+	-	+	-	-	+	+	-	-	+	+	-	+	-	Cy	C
TA1-04 / TB1-04	+	+	+	+	-	-	-	-	+	-	+	-	+	+	+	-	-	+	+	-	+	C	C
TA1-05 / TB1-05	+	+	+	+	+	-	-	-	-	+	-	+	+	-	+	+	-	-	+	+	-	Cy	S
TA1-06 / TB1-06	-	+	+	+	+	+	-	-	-	-	+	-	+	-	-	+	+	-	-	+	+	C	C
TA1-07 / TB1-07	+	-	+	+	+	+	+	-	-	-	-	+	+	+	-	-	+	+	-	-	+	Cy	S
TA1-08 / TB1-08	-	+	-	+	+	+	+	+	-	-	-	-	+	-	+	-	-	+	+	-	-	Cy	C
TA1-09 / TB1-09	+	-	+	-	+	+	+	+	+	-	-	-	+	+	-	+	-	-	+	+	-	C	C
TA1-10 / TB1-10	+	+	-	+	-	+	+	+	+	+	-	-	+	-	+	-	+	-	-	+	+	C	S
TA1-11 / TB1-11	-	+	+	-	+	-	+	+	+	+	+	-	+	-	-	+	-	+	-	-	+	Cy	S
TA1-12 / TB1-12	-	-	+	+	-	+	-	+	+	+	+	+	+	-	-	-	+	-	+	-	-	Cy	C
TA1-13 / TB1-13	+	-	-	+	+	-	+	-	+	+	+	+	+	-	-	-	-	+	-	+	-	C	C
TA1-14 / TB1-14	+	+	-	-	+	+	-	+	-	+	+	+	+	+	-	-	-	-	+	-	+	C	S
TA1-15 / TB1-15	-	+	+	-	-	+	+	-	+	-	+	+	+	+	+	-	-	-	-	+	-	Cy	S
TA1-16 / TB1-16	-	-	+	+	-	-	+	+	-	+	-	+	+	+	+	+	-	-	-	-	+	C	C
TA1-17 / TB1-17	+	-	-	+	+	-	-	+	+	-	+	-	+	+	+	+	+	-	-	-	-	Cy	S
TA1-18 / TB1-18	-	+	-	-	+	+	-	-	+	+	-	+	+	+	+	+	+	+	-	-	-	C	C
TA1-19 / TB1-19	+	-	+	-	-	+	+	-	-	+	+	-	+	-	+	+	+	+	+	-	-	C	S
TA1-20 / TB1-20	-	+	-	+	-	-	+	+	-	-	+	+	+	+	-	+	+	+	+	+	-	C	S
TA1-21 / TB1-21	-	-	+	-	+	-	-	+	+	-	-	+	+	-	+	-	+	+	+	+	+	C	S
TA1-22 / TB1-22	-	-	-	+	-	+	-	-	+	+	-	-	+	+	-	+	-	+	+	+	+	Cy	S
TA1-23 / TB1-23	-	-	-	-	+	-	+	-	-	+	+	-	+	+	+	-	+	-	+	+	+	Cy	C
TA1-24 / TB1-24	-	-	-	-	-	-	-	-	-	-	-	-	+	-	-	-	-	-	-	-	-	C	S

The measured response values, i.e., density, hardness, and roughness, is shown in Figure S1. As observed in these figures, the specimens that were replicated under the same processing parameters exhibit similar response values in most cases, affirming the repeatability of the printing process and the reliability of the data for variance analysis. This repeatability is notably more pronounced in density and hardness data and less so in roughness data. Hardness responses typically exhibit the largest standard error values.

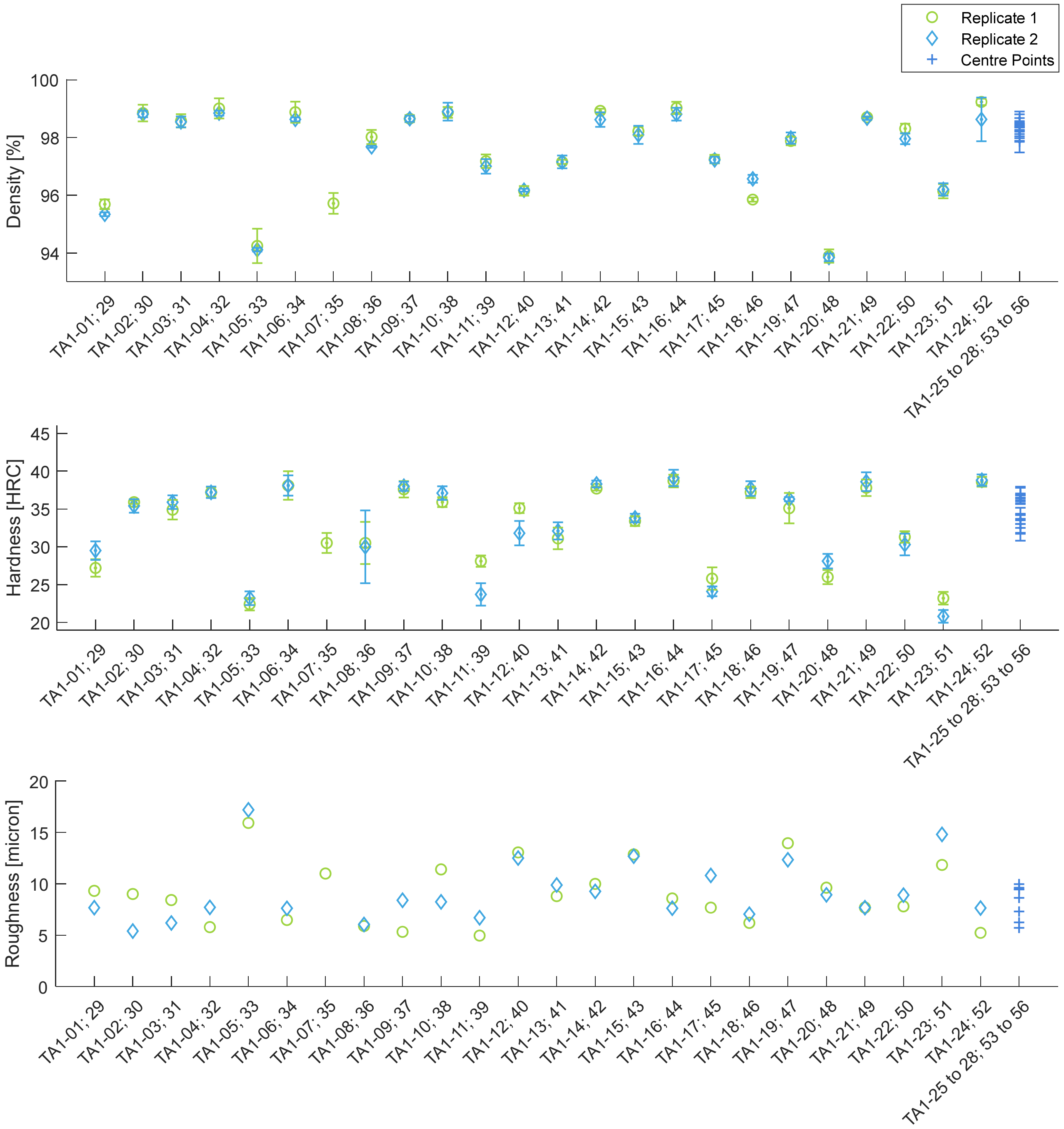

Figure S1. The measured response values for density, hardness, and roughness in Design A1. To facilitate comparison, the first and second replicates of runs sharing the same process parameters are co-located on the y-axis. The Y-axis labels, such as TA1-01; 29, indicate that the measured values correspond to parts #01 and #29 in Design A1, both printed with an identical set of process parameters.

Table S3. ANOVA results for the reduced model in Design A1, with density as the response, at a significance level of 95%. Significant parameters are denoted by asterisks, with the number of asterisks reflecting the statistical significance level—more asterisks indicate higher statistical significance (lower P-value).

**ANOVA: A1 - Density**	**Adj MS**	**F-Value**	**P-Value**

Model	0.011872	65.49	0.000
Linear	0.012063	66.54	0.000
Post-Contour Speed**	0.002848	15.71	0.000
Post-Contour Power**	0.009274	51.16	0.000
Skin-Energy Density**	0.006238	34.41	0.000
Skin-Power*	0.000803	4.43	0.042
Skin-Stripe Overlap**	0.012543	69.19	0.000
Skin-Thickness XY*	0.001448	7.99	0.008
Skin-Thickness Z**	0.009832	54.24	0.000
Core-Energy Density***	0.035049	193.35	0.000
Core-Power****	0.054817	302.39	0.000
Core-Stripe/Square Overlap**	0.012058	66.52	0.000
Support Area Spacing**	0.004274	23.58	0.000
Support Height**	0.009976	55.03	0.000
Layer Thickness*	0.001455	8.02	0.008
Angle**	0.005573	30.74	0.000
Print Location**	0.014516	80.07	0.000
Geometry***	0.020737	114.39	0.000
Hatching Method**	0.002259	12.46	0.001
Curvature	0.009364	51.65	0.000

Table S4. ANOVA results for the reduced model in Design A1, with hardness as the response, at a significance level of 95%. Significant parameters are denoted by asterisks, with the number of asterisks reflecting the statistical significance level—more asterisks indicate higher statistical significance (lower P-value).

**ANOVA: A1 - Hardness**	**Adj MS**	**F-Value**	**P-Value**
Model	73.663	42.93	0.000
Linear	76.398	44.52	0.000
Post-Contour Speed**	57.773	33.67	0.000
Post-Contour Power**	47.004	27.39	0.000
Skin-Energy Density**	89.125	51.94	0.000
Skin-Power**	86.055	50.15	0.000
Skin-Hatch Distance**	24.532	14.30	0.001
Skin-Stripe Overlap**	22.873	13.33	0.001
Skin-Thickness XY*	12.327	7.18	0.011
Skin-Thickness Z**	113.813	66.33	0.000
Core-Power**	87.523	51.01	0.000
Core-Stripe/Square Width**	24.879	14.50	0.001
Core-Stripe/Square Overlap**	40.699	23.72	0.000
Support Height*	10.740	6.26	0.017
Layer Thickness*	10.783	6.28	0.017
Angle**	50.263	29.29	0.000
Print Location*	11.349	6.61	0.014
Geometry****	537.780	313.42	0.000
Hatching Method**	34.199	19.93	0.000
Curvature	27.916	16.27	0.000

Table S5. ANOVA results for the reduced model in Design A1, with roughness as the response, at a significance level of 95%. Significant parameters are denoted by asterisks, with the number of asterisks reflecting the statistical significance level—more asterisks indicate higher statistical significance (lower P-value).

ANOVA: A1 - Roughness	Adj MS	F-Value	P-Value
Model	25.343	7.59	0.000
Linear	27.428	8.21	0.000
Skin-Stripe Width*	17.266	5.17	0.028
Skin-Thickness XY****	50.830	15.22	0.000
Skin-Thickness Z**	28.319	8.48	0.006
Core-Energy Density*	21.839	6.54	0.014
Core-Stripe/Square Width***	33.739	10.10	0.003
Support Height***	42.837	12.82	0.001
Layer Thickness*	27.723	8.30	0.006
Angle*	23.748	7.11	0.011
Print Location*	20.901	6.26	0.016
Hatching Method*	15.511	4.64	0.037
Curvature	3.602	1.08	0.305

Table S6. Significant parameters at a 95% confidence level for density, hardness, and roughness, based on the ANOVA of Design A1. Parameters marked with (*) have been selected for further study under Design A2 and shown in the left column.

No.	Density	Hardness	Roughness
1	Core-Power*	Geometry*	Skin-Thickness XY*
2	Core-Energy Density*	Skin-Thickness Z*	Support Height*
3	Geometry*	Skin-Energy Density*	Core-Stripe Width*
4	Print Location*	Core-Power*	Skin-Thickness Z*
5	Skin-Stripe Overlap*	Skin-Power	Layer Thickness*
6	Core-Stripe Overlap	Post-Contour Speed	Angle*
7	Support Height*	Angle	Core-Energy Density*
8	Skin-Thickness Z*	Post-Contour Power*	Print Location*
9	Post-Contour Power*	Core-Stripe Overlap	Skin-Stripe Width
10	Skin-Energy Density*	Hatching Method*	Hatching Method*
11	Angle	Core-Stripe Width*	
12	Support Area Spacing	Skin-Hatch Distance*	
13	Post-Contour Speed	Skin-Stripe Overlap	
14	Hatching Method*	Skin-Thickness XY*	
15	Layer Thickness*	Print Location*	
16	Skin-Thickness XY*	Layer Thickness*	
17	Skin-Power	Support Height*	

→

No.	Selected for A2
1	Core-Power
2	Core-Energy Density
3	Geometry
4	Print Location
5	Skin-Stripe Overlap
6	Post-Contour Power
7	Core-Stripe Width
8	Support Height
9	Skin-Energy Density
10	Skin-Thickness Z
11	Skin-Thickness XY
12	Skin-Hatch Distance
13	Layer Thickness
14	Hatching Method

2 Design B1

The parameter levels and alias structure for design B1 are identical to those of design A1. For more information, refer to In designs A1 and B1, 23 process parameters were investigated, comprising 21 continuous and 2 categorical variables. Consequently, 24 runs were conducted to evaluate the effects of the main parameters. To enhance the robustness and validity of the designs

and reduce experimental error, two replicates of the designs and 8 center point runs were included, increasing the total number of runs for each design to 56. Table S1 provides details on the process parameters studied, along with their respective lower and upper limits.

Table S1 and Table S2.

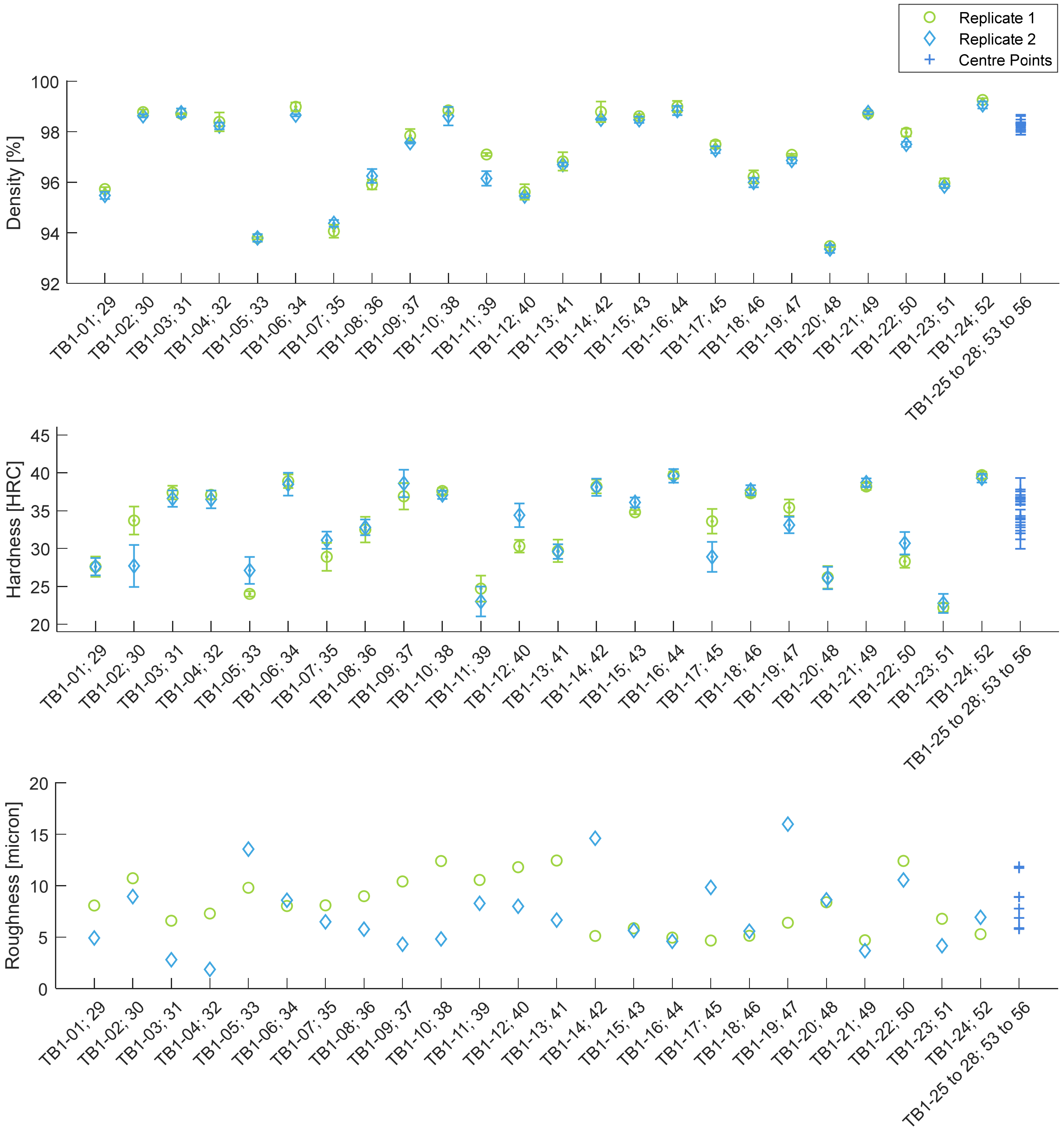

Figure S2. The measured response values for density, hardness, and roughness in Design B1. To facilitate comparison, the first and second replicates of runs sharing the same process parameters are co-located on the y-axis. The Y-axis labels, such as TB1-01; 29, indicate that the measured values correspond to parts #01 and #29 in Design B1, both printed with an identical set of process parameters.

Table S7. ANOVA results for the reduced model in Design B1, with density as the response, at a significance level of 95%. Significant parameters are denoted by asterisks, with the number of asterisks reflecting the statistical significance level—more asterisks indicate higher statistical significance (lower P-value).

**ANOVA: B1 - Density**	**Adj MS**	**F-Value**	**P-Value**

Model	0.017536	72.79	0.000
Linear	0.017598	73.05	0.000
Post-Contour Speed*	0.001173	4.87	0.033
Post-Contour Power**	0.022445	93.17	0.000
Skin-Energy Density**	0.014966	62.12	0.000
Skin-Hatch Distance**	0.005846	24.27	0.000
Skin-Stripe Overlap**	0.018410	76.42	0.000
Skin-Thickness Z**	0.006041	25.08	0.000
Core-Energy Density***	0.028436	118.04	0.000
Core-Power****	0.071550	297.01	0.000
Core-Stripe/Square Overlap**	0.009341	38.77	0.000
Support Area Spacing**	0.003461	14.37	0.001
Support Height***	0.025717	106.75	0.000
Layer Thickness**	0.010642	44.17	0.000
Print Location**	0.018896	78.44	0.000
Geometry***	0.025098	104.18	0.000
Hatching Method*	0.001952	8.10	0.007
Curvature	0.016600	68.91	0.000

Table S8. ANOVA results for the reduced model in Design B1, with hardness as the response, at a significance level of 95%. Significant parameters are denoted by asterisks, with the number of asterisks reflecting the statistical significance level—more asterisks indicate higher statistical significance (lower P-value).

**ANOVA: B1 - Hardness**	**Adj MS**	**F-Value**	**P-Value**
Model	76.914	31.35	0.000
Linear	80.252	32.72	0.000
Post-Contour Speed**	62.221	25.36	0.000
Post-Contour Power*	14.246	5.81	0.021
Skin-Energy Density**	34.425	14.03	0.001
Skin-Power**	76.129	31.03	0.000
Skin-Hatch Distance**	87.615	35.72	0.000
Skin-Stripe Width*	12.251	4.99	0.031
Skin-Thickness XY**	26.181	10.67	0.002
Skin-Thickness Z***	126.263	51.47	0.000
Core-Power***	123.681	50.42	0.000
Core-Hatch Distance*	10.221	4.17	0.048
Core-Stripe/Square Overlap**	62.449	25.46	0.000
Support Height**	54.081	22.05	0.000
Layer Thickness**	43.035	17.54	0.000
Angle**	36.838	15.02	0.000
Geometry****	498.913	203.39	0.000
Hatching Method*	15.488	6.31	0.016
Curvature	23.495	9.58	0.004

Table S9. ANOVA results for the reduced model in Design B1, with roughness as the response, at a significance level of 95%. Significant parameters are denoted by asterisks, with the number of asterisks reflecting the statistical significance level—more asterisks indicate higher statistical significance (lower P-value).

**ANOVA: B1 - Roughness**	**Adj MS**	**F-Value**	**P-Value**
Model	10.4651	1.22	0.300

Linear	10.7514	1.25	0.277
Pre-Contour Speed	11.5590	1.34	0.255
Pre-Contour Power	0.7086	0.08	0.776
Post-Contour Speed	3.8095	0.44	0.511
Post-Contour Power	15.3570	1.79	0.191
Skin-Energy Density	0.1154	0.01	0.909
Skin-Power	13.9565	1.62	0.212
Skin-Hatch Distance	2.2885	0.27	0.610
Skin-Stripe Width	5.5439	0.64	0.428
Skin-Stripe Overlap	0.1933	0.02	0.882
Skin-Thickness XY*	29.9590	3.48	0.071
Skin-Thickness Z	4.7437	0.55	0.463
Core-Energy Density	0.1467	0.02	0.897
Core-Power	1.0144	0.12	0.734
Core-Hatch Distance*	34.2976	3.99	0.055
Core-Stripe/Square Width*	32.0276	3.73	0.063
Core-Stripe/Square Overlap*	27.8345	3.24	0.082
Support Area Spacing	1.2300	0.14	0.708
Support Height	1.8985	0.22	0.642
Layer Thickness*	26.4850	3.08	0.089
Angle	0.0586	0.01	0.935
Print Location	1.6238	0.19	0.667
Geometry	13.5523	1.58	0.219
Hatching Method	18.8785	2.20	0.148
Curvature	3.8818	0.45	0.507

Table S10. Significant parameters at a 95% confidence level for density, hardness, and roughness, based on the ANOVA of Design B1. Parameters marked with (*) have been selected for further study under Design B2 and shown in the left column.

No.	Density	Hardness	Roughness
1	Core-Power*	Geometry*	Core-Hatch Distance*
2	Core-Energy Density*	Skin-thickness Z*	Core-Stripe Width*
3	Support Height*	Core-Power*	Skin-Thickness XY*
4	Geometry*	Skin-Hatch Distance	Core-Stripe Overlap*
5	Post-Contour Power*	Skin-Power	Layer Thickness*
6	Print Location*	Core-Stripe Overlap*	Hatching Method*
7	Skin-Stripe Overlap*	Post-Contour Speed	Post-Contour Power*
8	Skin-Energy Density*	Support Height*	Skin-Power
9	Layer Thickness*	Layer Thickness*	Geometry*
10	Core-Stripe Overlap*	Angle	Pre-Contour Speed
11	Skin-Thickness Z*	Skin-Energy Density*	
12	Skin-Hatch Distance	Skin-Thickness XY*	
13	Support Area Spacing	Post-Contour Power*	
14	Hatching Method*	Skin-Stripe Width	
15	Post-Contour Speed	Core-Hatch Distance*	

→

No.	Selected for B2
1	Core-Power
2	Core-Energy Density
3	Core-Hatch Distance
4	Skin-Energy Density
5	Skin-Stripe Overlap
6	Skin-Thickness XY
7	Core-Stripe Width
8	Skin-Thickness Z
9	Layer Thickness
10	Support Height
11	Post-Contour Power
12	Hatching Method
13	Geometry
14	Print Location
15	Core-Stripe Overlap

3 Design C1

Due to the challenges associated with processing cylindrical samples – most notably, the time-consuming roughness characterization of curved surfaces – geometry was excluded as a

parameter under study for powder C. Instead, a unified 10×10×10 mm^3 part was utilized for Designs C1 and C2. Furthermore, the angle was excluded from the study in Designs C1, A2, B2, and C2 to facilitate fitting all parts onto a single plate as parts oriented at 30° or 60° occupied more space. Additionally, it was observed that angled parts resulted in noisier data, especially concerning roughness, due to unoptimized upskin and downskin parameters. Hence, Design C1 focused on studying 21 process parameters. To maintain consistency, the number of runs in this design was kept similar to A1 and B1, totaling 56 runs, inclusive of 2 replicates and 8 center points. The process parameter ranges and design structure for C1 are displayed in Table S11 and Table S12, respectively.

Table S11. Categories, types, and levels of process parameters for Design C1.

Category	No.	Code	Parameter	Type	Low (-1)	Mid (0)	High (+1)
**Pre-exposure**	1	A	Contour Speed [mm/s]	Continuous	700	1100	1500
	2	B	Contour Power [W]	Continuous	10	35	60
**Post-exposure**	3	C	Contour Speed [mm/s]	Continuous	700	1100	1500
	4	D	Contour Power [W]	Continuous	10	85	160
**Skin-exposure**	5	E	Energy Density [J/mm^2]	Continuous	1.5	2.75	4
	6	F	Power [W]	Continuous	150	250	350
	7	G	Hatch Distance [mm]	Continuous	0.08	0.1	0.12
	8	H	Stripe Width [mm]	Continuous	2	3.5	5
	9	J	Stripe Overlap [mm]	Continuous	0.01	0.08	0.15
	10	K	Skin Thickness X/Y [mm]	Continuous	0.2	0.6	1
	11	L	Skin Thickness Z [mm]	Continuous	0.2	0.6	1
**Core-exposure**	12	M	Energy Density [J/mm^2]	Continuous	1.5	2.75	4
	13	N	Power [W]	Continuous	150	250	350
	14	O	Hatch Distance [mm]	Continuous	0.08	0.09	0.1
	15	P	Stripe/Square Width [mm]	Continuous	2	3.5	5
	16	Q	Stripe/Square Overlap [mm]	Continuous	0.01	0.08	0.15
**Support Structure**	17	R	Support Area Spacing [mm]	Continuous	0.6	0.7	0.8
	18	S	Support Height [mm]	Continuous	2	5	8
**Other**	19	T	Layer Thickness [mm]	Continuous	0.02	0.04	0.06
	20		Angle [deg]	Fixed: 90			
	21	U	Print Location [mm]	Categorical	Section A	N/A	Section B
	22		Geometry	Fixed: Cube			
	23	V	Hatching Method	Categorical	Stripe	N/A	Chess

Table S12. P-B Design C1, including only the first replicate for brevity. The second replicate and runs at center points are excluded. The second replicate shares the exact alias as the first one. For center points, all continuous parameters are fixed at 0, and the categorical ones alternate between + and -. For the parameter U (Print Location), 'A' denotes Region A, and 'B' denotes Region B. For the parameter V (Hatching Method), 'S' denotes Stripe and 'C' denotes Chess.

	**A**	**B**	**C**	**D**	**E**	**F**	**G**	**H**	**J**	**K**	**L**	**M**	**N**	**O**	**P**	**Q**	**R**	**S**	**T**	**U**	**V**
TC1-01	+	-	-	-	-	+	-	+	-	-	+	+	-	-	+	+	-	+	-	B	C
TC1-02	+	+	-	-	-	-	+	-	+	-	-	+	+	-	-	+	+	-	+	A	C
TC1-03	+	+	+	-	-	-	-	+	-	+	-	-	+	+	-	-	+	+	-	B	S
TC1-04	+	+	+	+	-	-	-	-	+	-	+	-	-	+	+	-	-	+	+	A	C

TC1-05	+	+	+	+	+	-	-	-	-	+	-	+	-	-	+	+	-	-	+	B	S
TC1-06	-	+	+	+	+	+	-	-	-	-	+	-	+	-	-	+	+	-	-	B	C
TC1-07	+	-	+	+	+	+	+	-	-	-	-	+	-	+	-	-	+	+	-	A	C
TC1-08	-	+	-	+	+	+	+	+	-	-	-	-	+	-	+	-	-	+	+	A	S
TC1-09	+	-	+	-	+	+	+	+	+	-	-	-	-	+	-	+	-	-	+	B	S
TC1-10	+	+	-	+	-	+	+	+	+	+	-	-	-	-	+	-	+	-	-	B	C
TC1-11	-	+	+	-	+	-	+	+	+	+	+	-	-	-	-	+	-	+	-	A	C
TC1-12	-	-	+	+	-	+	-	+	+	+	+	+	-	-	-	-	+	-	+	A	S
TC1-13	+	-	-	+	+	-	+	-	+	+	+	+	+	-	-	-	-	+	-	B	S
TC1-14	+	+	-	-	+	+	-	+	-	+	+	+	+	+	-	-	-	-	+	A	C
TC1-15	-	+	+	-	-	+	+	-	+	-	+	+	+	+	+	-	-	-	-	B	S
TC1-16	-	-	+	+	-	-	+	+	-	+	-	+	+	+	+	+	-	-	-	A	C
TC1-17	+	-	-	+	+	-	-	+	+	-	+	-	+	+	+	+	+	-	-	A	S
TC1-18	-	+	-	-	+	+	-	-	+	+	-	+	-	+	+	+	+	+	-	A	S
TC1-19	+	-	+	-	-	+	+	-	-	+	+	-	+	-	+	+	+	+	+	A	S
TC1-20	-	+	-	+	-	-	+	+	-	-	+	+	-	+	-	+	+	+	+	B	S
TC1-21	-	-	+	-	+	-	-	+	+	-	-	+	+	-	+	-	+	+	+	B	C
TC1-22	-	-	-	+	-	+	-	-	+	+	-	-	+	+	-	+	-	+	+	B	C
TC1-23	-	-	-	-	+	-	+	-	-	+	+	-	-	+	+	-	+	-	+	B	C
TC1-24	-	-	-	-	-	-	-	-	-	-	-	-	-	-	-	-	-	-	-	A	S

Figure S3 illustrates the response values for C1, showcasing an overall improvement in data quality following the modifications made to Design C1 based on the experience gained from A1 and B1. This improvement is evident through reduced disparities among the measured response values for replicated parts and smaller standard error values.

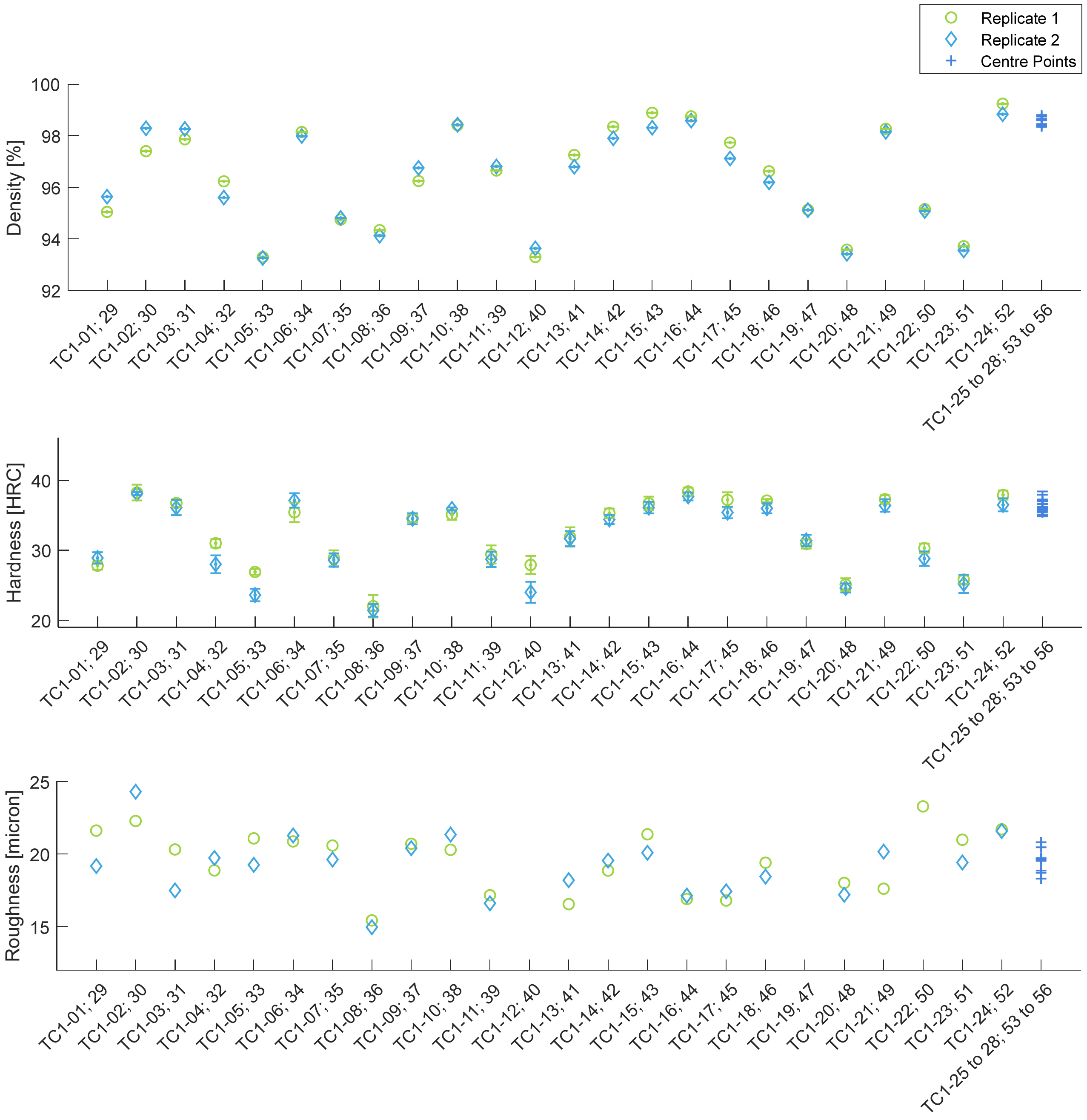

Figure S3. The measured response values for density, hardness, and roughness in Design C1. To facilitate comparison, the first and second replicates of runs sharing the same process parameters are co-located on the y-axis. The Y-axis labels, such as TC1-01; 29, indicate that the measured values correspond to parts #01 and #29 in Design C1, both printed with an identical set of process parameters.

Table S13. ANOVA results for the reduced model in Design C1, with density as the response, at a significance level of 95%. Significant parameters are denoted by asterisks, with the number of asterisks reflecting the statistical significance level—more asterisks indicate higher statistical significance (lower P-value).

ANOVA: C1 - Density	Adj MS	F-Value	P-Value
Model	0.038548	21.43	0.000

Linear	0.034939	19.42	0.000
Post-Contour Power**	0.030786	17.11	0.000
Skin-Stripe Overlap*	0.015716	8.74	0.005
Skin-Thickness Z*	0.009451	5.25	0.026
Core-Power***	0.062135	34.54	0.000
Support Height**	0.026032	14.47	0.000
Layer Thickness****	0.091194	50.69	0.000
Hatching Method*	0.009261	5.15	0.028
Curvature	0.063808	35.47	0.000

Table S14. ANOVA results for the reduced model in Design C1, with hardness as the response, at a significance level of 95%. Significant parameters are denoted by asterisks, with the number of asterisks reflecting the statistical significance level—more asterisks indicate higher statistical significance (lower P-value).

**ANOVA: C1 - Hardness**	**Adj MS**	**F-Value**	**P-Value**
Model	138.441	19.44	0.000
Linear	139.842	19.64	0.000
Post-Contour Power**	144.292	20.26	0.000
Skin-Stripe Overlap**	84.418	11.85	0.001
Skin-Thickness Z*	68.013	9.55	0.003
Core-Power***	180.432	25.34	0.000
Support Height**	130.119	18.27	0.000
Layer Thickness****	231.781	32.55	0.000
Curvature	130.031	18.26	0.000

Table S15. ANOVA results for the reduced model in Design C1, with roughness as the response, at a significance level of 95%. Significant parameters are denoted by asterisks, with the number of asterisks reflecting the statistical significance level—more asterisks indicate higher statistical significance (lower P-value).

**ANOVA: C1 - Roughness**	**Adj MS**	**F-Value**	**P-Value**
Model	41.107	27.93	0.000
Linear	43.598	29.63	0.000
Pre-Contour Power**	60.218	40.92	0.000
Post-Contour Speed**	25.718	17.48	0.000
Post-Contour Power*	6.986	4.75	0.035
Skin-Energy Density****	131.074	89.07	0.000
Skin-Power***	97.879	66.51	0.000
Skin-Hatch Distance*	9.247	6.28	0.016
Skin-Stripe Width**	54.170	36.81	0.000
Skin-Thickness XY**	30.977	21.05	0.000
Skin-Thickness Z*	8.466	5.75	0.021
Core-Hatch Distance**	39.403	26.78	0.000
Core-Stripe/Square Width*	13.133	8.92	0.005
Support Area Spacing**	48.564	33.00	0.000
Support Height*	13.936	9.47	0.004
Layer Thickness***	70.597	47.97	0.000
Curvature	6.240	4.24	0.046

Table S16. Significant parameters at a 95% confidence level for density, hardness, and roughness, based on the ANOVA of Design C1. Parameters marked with (*) have been selected for further study under Design C2 and shown in the left column.

No.	Density	Hardness	Roughness
1	Layer Thickness*	Layer Thickness*	Skin-Energy Density*
2	Core-Power*	Core-Power*	Skin-Power*
3	Post-Contour Power*	Post-Contour Power*	Layer Thickness*
4	Support Height*	Support Height*	Pre-Contour Power*
5	Skin-Stripe Overlap*	Skin-Stripe Overlap*	Skin-Stripe Width*
6	Skin-Thickness Z*	Skin-Thickness Z*	Support Area Spacing*
7	Hatching Method*		Core-Hatch Distance
8			Skin-Thickness XY
9			Post-Contour Speed
10			Support Height*
11			Core-Stripe Width
12			Skin-Hatch Distance
13			Skin-Thickness Z*
14			Post-Contour Power*

→

No.	Selected for C2
1	Layer Thickness
2	Core-Power
3	Post-Contour Power
4	Support Height
5	Skin-Stripe Overlap
6	Skin-Thickness Z
7	Skin-Energy Density
8	Skin-Power
9	Pre-Contour Power
10	Core-Energy Density^
11	Core-Hatch Distance
12	Support Area Spacing
13	Hatching Method

^ added by operator

4 Design A2

High-resolution FFDs with a manageable number of 64 runs were designed: 2_{IV}^{14-8} design for powder A, 2_{IV}^{15-9} design for powder B, and 2_{IV}^{13-7} design for powder C, where 2 is the number of levels, superscripts indicate the extent of fraction, and subscripts show the resolution of the designs. To enhance precision, the designs were replicated twice, and 12 center points were added per each replication to test for the presence of curvature, increasing the total number of runs to 152 in Designs A2 and B2. The total number of runs in Design C2 was 138, a result of having fewer categorical factors (one compared to three in A2 and B2), therefore, requiring fewer center points. Table S17, Table S22, and Table S27 provide details on the process parameters investigated, along with their respective lower and upper limits for Designs A2, B2, and C2, respectively. The coded structure for these designs can be found in Table S18, Table S23, and Table S28. Additionally, the measured density, hardness, and roughness values are shown in Figure S4, Figure S5, and Figure S6 for designs A2, B2, and C2, respectively. Similar trends in terms of repeatability and error values, as observed in P-B designs, can be seen in these figures. Overall, the quality of the data appears to have slightly improved with the changes made to the parameter selections, and there are no observable concerns based on the plotted data.

Table S17. Categories, types, and levels of process parameters for Design A2. Parameters with a fixed value are deemed statistically insignificant based on the ANOVA of Design A1 and, therefore, have been excluded from further study.

Category	No.	Code	Parameter	Type	Low (-1)	Mid (0)	High (+1)
**Pre-exposure**	-	-	Contour Speed [mm/s]	Fixed: 1500			
	-	-	Contour Power [W]	Fixed: 30			
**Post-exposure**	-	-	Contour Speed [mm/s]	Fixed: 1500			
	1	A	Contour Power [W]	Continuous	0	80	160
**Skin-exposure**	2	B	Energy Density [J/mm^2]	Continuous	1.5	2.75	4
	-	-	Power [W]	Fixed: 150			

	3	C	Hatch Distance [mm]	Continuous	0.08	0.1	0.12
	-	-	Stripe Width [mm]	Fixed: 3.5			
	4	D	Stripe Overlap [mm]	Continuous	0.01	0.08	0.15
	5	E	Skin Thickness X/Y [mm]	Continuous	0.2	0.6	1
	6	F	Skin Thickness Z [mm]	Continuous	0.2	0.6	1
**Core-exposure**	7	G	Energy Density [J/mm^2]	Continuous	1.5	2.75	4
	8	H	Power [W]	Continuous	150	250	350
	-	-	Hatch Distance [mm]	Fixed: 0.09			
	9	J	Stripe/Square Width [mm]	Continuous	2	3.5	5
	-	-	Stripe/Square Overlap [mm]	Fixed: 0.01			
**Support Structure**	-	-	Support Area Spacing [mm]	Fixed: 0.6			
	10	K	Support Height [mm]	Continuous	0	4	8
**Other**	11	L	Layer Thickness [mm]	Continuous	0.02	0.04	0.06
	-	-	Angle [deg]	Fixed: 90			
	12	M	Print Location [mm]	Categorical	Section A	N/A	Section B
	13	N	Geometry	Categorical	Cube	N/A	Cylinder
	14	O	Hatching Method	Categorical	Stripe	N/A	Chess

Table S18. FFD Design A2, including only the first replicate for brevity. The second replicate and runs at center points are excluded. The second replicate shares the exact alias as the first one. For center points, all continuous parameters are fixed at 0, and the categorical ones alternate between + and -. For the parameter N (Geometry), 'Cy' denotes Cylinder, and 'C' denotes Cube. For the parameter O (Hatching Method), 'S' denotes Stripe and 'C' denotes Chess.

	**A**	**B**	**C**	**D**	**E**	**F**	**G**	**H**	**J**	**K**	**L**	**M**	**N**	**O**
TA2-01	-	-	-	-	-	-	-	-	-	-	-	A	Cy	S
TA2-02	+	-	-	-	-	-	-	-	-	-	-	A	C	S
TA2-03	-	+	-	-	-	-	+	+	-	-	+	A	Cy	C
TA2-04	+	+	-	-	-	-	+	+	-	-	+	A	C	C
TA2-05	-	-	+	-	-	-	-	+	-	+	+	B	C	C
TA2-06	+	-	+	-	-	-	-	+	-	+	+	B	Cy	C
TA2-07	-	+	+	-	-	-	+	-	-	+	-	B	C	S
TA2-08	+	+	+	-	-	-	+	-	-	+	-	B	Cy	S
TA2-09	-	-	-	+	-	-	-	-	+	-	-	B	C	C
TA2-10	+	-	-	+	-	-	-	-	+	-	-	B	Cy	C
TA2-11	-	+	-	+	-	-	+	+	+	-	+	B	C	S
TA2-12	+	+	-	+	-	-	+	+	+	-	+	B	Cy	S
TA2-13	-	-	+	+	-	-	-	+	+	+	+	A	Cy	S
TA2-14	+	-	+	+	-	-	-	+	+	+	+	A	C	S
TA2-15	-	+	+	+	-	-	+	-	+	+	-	A	Cy	C
TA2-16	+	+	+	+	-	-	+	-	+	+	-	A	C	C
TA2-17	-	-	-	-	+	-	+	-	+	+	+	A	C	C
TA2-18	+	-	-	-	+	-	+	-	+	+	+	A	Cy	C
TA2-19	-	+	-	-	+	-	-	+	+	+	-	A	C	S
TA2-20	+	+	-	-	+	-	-	+	+	+	-	A	Cy	S
TA2-21	-	-	+	-	+	-	+	+	+	-	-	B	Cy	S

TA2-22	+	-	+	-	+	-	+	+	+	-	-	B	C	S
TA2-23	-	+	+	-	+	-	-	-	+	-	+	B	Cy	C
TA2-24	+	+	+	-	+	-	-	-	+	-	+	B	C	C
TA2-25	-	-	-	+	+	-	+	-	-	+	+	B	Cy	S
TA2-26	+	-	-	+	+	-	+	-	-	+	+	B	C	S
TA2-27	-	+	-	+	+	-	-	+	-	+	-	B	Cy	C
TA2-28	+	+	-	+	+	-	-	+	-	+	-	B	C	C
TA2-29	-	-	+	+	+	-	+	+	-	-	-	A	C	C
TA2-30	+	-	+	+	+	-	+	+	-	-	-	A	Cy	C
TA2-31	-	+	+	+	+	-	-	-	-	-	+	A	C	S
TA2-32	+	+	+	+	+	-	-	-	-	-	+	A	Cy	S
TA2-33	-	-	-	-	-	+	+	+	+	+	-	B	Cy	C
TA2-34	+	-	-	-	-	+	+	+	+	+	-	B	C	C
TA2-35	-	+	-	-	-	+	-	-	+	+	+	B	Cy	S
TA2-36	+	+	-	-	-	+	-	-	+	+	+	B	C	S
TA2-37	-	-	+	-	-	+	+	-	+	-	+	A	C	S
TA2-38	+	-	+	-	-	+	+	-	+	-	+	A	Cy	S
TA2-39	-	+	+	-	-	+	-	+	+	-	-	A	C	C
TA2-40	+	+	+	-	-	+	-	+	+	-	-	A	Cy	C
TA2-41	-	-	-	+	-	+	+	+	-	+	-	A	C	S
TA2-42	+	-	-	+	-	+	+	+	-	+	-	A	Cy	S
TA2-43	-	+	-	+	-	+	-	-	-	+	+	A	C	C
TA2-44	+	+	-	+	-	+	-	-	-	+	+	A	Cy	C
TA2-45	-	-	+	+	-	+	+	-	-	-	+	B	Cy	C
TA2-46	+	-	+	+	-	+	+	-	-	-	+	B	C	C
TA2-47	-	+	+	+	-	+	-	+	-	-	-	B	Cy	S
TA2-48	+	+	+	+	-	+	-	+	-	-	-	B	C	S
TA2-49	-	-	-	-	+	+	-	+	-	-	+	B	C	S
TA2-50	+	-	-	-	+	+	-	+	-	-	+	B	Cy	S
TA2-51	-	+	-	-	+	+	+	-	-	-	-	B	C	C
TA2-52	+	+	-	-	+	+	+	-	-	-	-	B	Cy	C
TA2-53	-	-	+	-	+	+	-	-	-	+	-	A	Cy	C
TA2-54	+	-	+	-	+	+	-	-	-	+	-	A	C	C
TA2-55	-	+	+	-	+	+	+	+	-	+	+	A	Cy	S
TA2-56	+	+	+	-	+	+	+	+	-	+	+	A	C	S
TA2-57	-	-	-	+	+	+	-	+	+	-	+	A	Cy	C
TA2-58	+	-	-	+	+	+	-	+	+	-	+	A	C	C
TA2-59	-	+	-	+	+	+	+	-	+	-	-	A	Cy	S
TA2-60	+	+	-	+	+	+	+	-	+	-	-	A	C	S
TA2-61	-	-	+	+	+	+	-	-	+	+	-	B	C	S
TA2-62	+	-	+	+	+	+	-	-	+	+	-	B	Cy	S

TA2-63	-	+	+	+	+	+	+	+	+	+	+	B	C	C
TA2-64	+	+	+	+	+	+	+	+	+	+	+	B	Cy	C

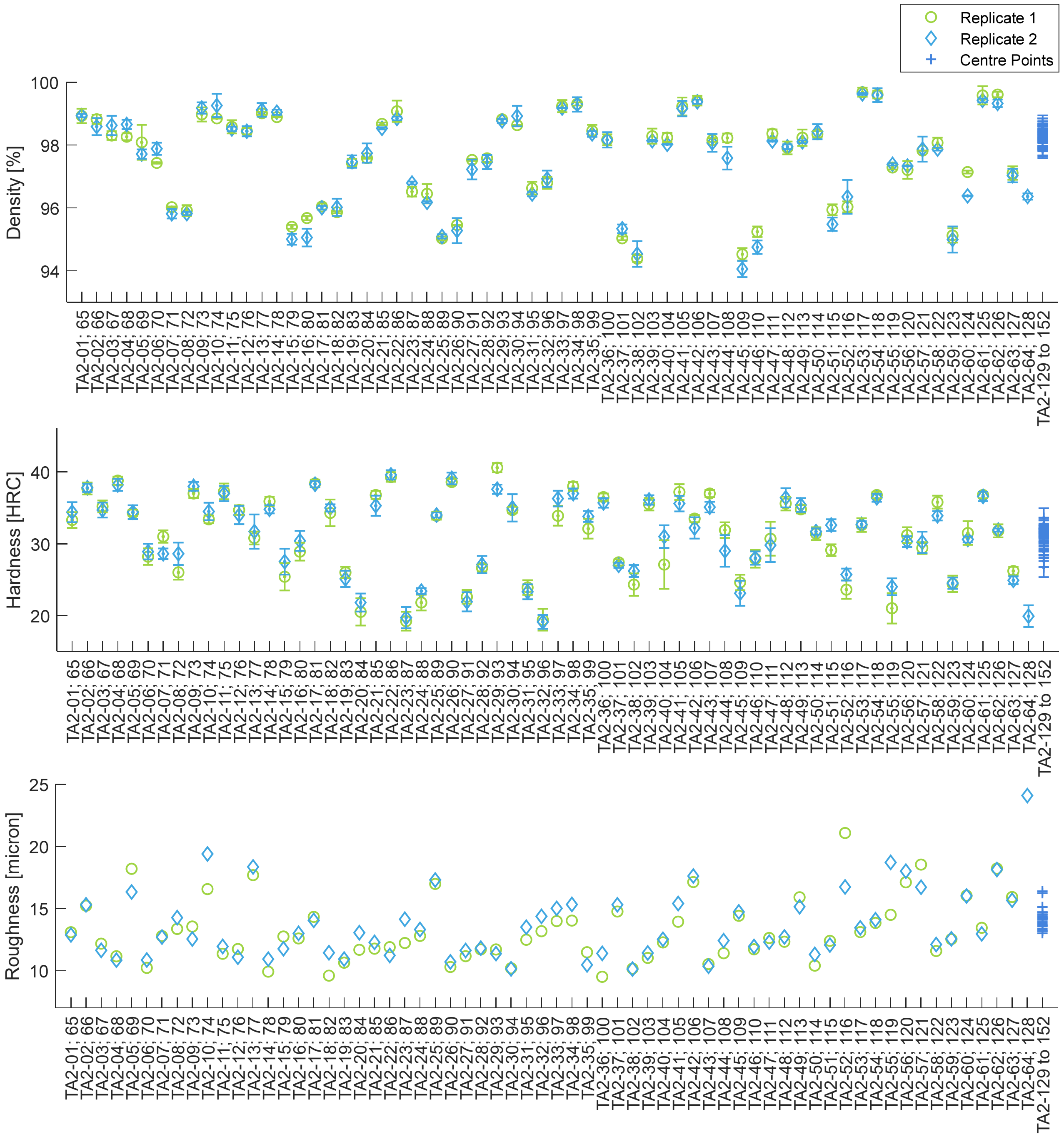

Figure S4. The measured response values for density, hardness, and roughness in Design A2. To facilitate comparison, the first and second replicates of runs sharing the same process parameters are co-located on the y-axis.

The Y-axis labels, such as TA2-01; 65, indicate that the measured values correspond to parts #01 and #65 in Design A2, both printed with an identical set of process parameters.

Table S19. ANOVA results and regression coefficient values for the reduced model in Design A2, with density as the response, at a significance level of 95%. Significant parameters are denoted by asterisks, with the number of asterisks reflecting the statistical significance level—more asterisks indicate higher statistical significance (lower P-value).

**ANOVA: A2 - Density**	**Adj MS**	**F-Value**	**P-Value**	**Reg. Coeff.**
Model	0.016895	112.89	0.000	4.4665
Linear	0.021752	145.34	0.000	
Post-Contour Power	0.000149	1.00	0.320	0.000119
Skin-Energy Density***	0.034534	230.76	0.000	- 0.03310
Skin-Hatch Distance**	0.009605	64.18	0.000	0.380
Skin-Stripe Overlap	0.000421	2.81	0.096	0.0374
Skin-Thickness XY**	0.006502	43.45	0.000	0.04413
Skin-Thickness Z	0.001026	6.85	0.010	- 0.01050
Core-Energy Density****	0.095836	640.37	0.000	- 0.03974
Core-Power****	0.109637	732.59	0.000	0.000344
Core-Stripe/Square Width	0.000244	1.63	0.204	0.00653
Support Height**	0.001980	13.23	0.000	0.005741
Layer Thickness***	0.042657	285.03	0.000	- 4.710
Print Location	0.000328	2.19	0.141	0.00556
Geometry	0.000305	2.04	0.156	0.00929
Hatching Method*	0.000640	4.27	0.041	- 0.00207
2-Way Interactions	0.012624	84.35	0.000	
Post-Contour Power × Skin-Hatch Distance*	0.000798	5.33	0.023	- 0.001569
Post-Contour Power × Skin-Thickness XY*	0.000911	6.09	0.015	0.000084
Post-Contour Power × Core-Energy Density*	0.000641	4.29	0.041	0.000023
Post-Contour Power × Layer Thickness*	0.000757	5.06	0.026	- 0.001527
Skin-Energy Density × Skin-Hatch Distance**	0.004979	33.27	0.000	- 0.2508
Skin-Energy Density × Skin-Thickness XY**	0.015218	101.69	0.000	- 0.02192
Skin-Energy Density × Skin-Thickness Z*	0.001300	8.68	0.004	0.00641
Skin-Energy Density × Core-Energy Density**	0.010393	69.44	0.000	0.005797
Skin-Energy Density × Core-Power*	0.000658	4.40	0.038	- 0.000018
Skin-Energy Density × Core-Stripe/Square Width**	0.001847	12.34	0.001	- 0.002036
Skin-Energy Density × Support Height**	0.009458	63.20	0.000	- 0.001728
Skin-Energy Density × Layer Thickness****	0.160426	1071.95	0.000	1.4234
Skin-Energy Density × Print Location*	0.000781	5.22	0.024	0.001986
Skin-Hatch Distance × Print Location*	0.000792	5.29	0.023	- 0.1250
Skin-Stripe Overlap × Skin-Thickness XY*	0.001111	7.43	0.007	- 0.1058
Skin-Stripe Overlap × Geometry*	0.000685	4.57	0.035	- 0.0332
Core-Energy Density × Geometry**	0.001699	11.35	0.001	- 0.002930
Curvature	0.020939	139.91	0.000	0.03281

Table S20. ANOVA results and regression coefficient values for the reduced model in Design A2, with hardness as the response, at a significance level of 95%. Significant parameters are denoted by asterisks, with the number of

asterisks reflecting the statistical significance level—more asterisks indicate higher statistical significance (lower P-value).

**ANOVA: A2 - Hardness**	**Adj MS**	**F-Value**	**P-Value**	**Reg. Coeff.**
Model	142.631	97.22	0.000	41.99
Linear	163.615	111.52	0.000	
Post-Contour Power	0.033	0.02	0.880	0.00020
Skin-Energy Density****	866.830	590.83	0.000	- 0.505
Skin-Hatch Distance***	309.385	210.88	0.000	- 47.2
Skin-Thickness XY***	241.078	164.32	0.000	11.992
Skin-Thickness Z*	6.674	4.55	0.035	- 8.352
Core-Energy Density*	13.153	8.96	0.003	- 0.326
Core-Power**	69.945	47.67	0.000	0.00743
Core-Stripe/Square Width	1.036	0.71	0.402	0.375
Support Height	0.057	0.04	0.844	0.4687
Layer Thickness**	70.962	48.37	0.000	- 165.8
Print Location	0.089	0.06	0.806	1.326
Geometry***	589.190	401.59	0.000	- 0.995
Hatching Method	4.281	2.92	0.090	0.193
2-Way Interactions	134.441	91.63	0.000	
Post-Contour Power × Geometry*	12.987	8.85	0.004	- 0.0040
Post-Contour Power × Hatching Method**	16.600	11.31	0.001	- 0.0045
Skin-Energy Density × Skin-Hatch Distance*	10.010	6.82	0.010	- 11.24
Skin-Energy Density × Skin-Thickness XY****	998.927	680.87	0.000	- 5.614
Skin-Energy Density × Skin-Thickness Z***	253.557	172.82	0.000	2.829
Skin-Energy Density × Core-Energy Density*	13.929	9.49	0.003	0.2122
Skin-Energy Density × Core-Stripe/Square Width*	11.154	7.60	0.007	- 0.1582
Skin-Energy Density × Support Height**	94.162	64.18	0.000	- 0.1724
Skin-Energy Density × Layer Thickness***	172.763	117.75	0.000	46.70
Skin-Hatch Distance × Print Location*	9.246	6.30	0.013	- 13.50
Skin-Thickness XY × Geometry*	5.881	4.01	0.047	- 0.538
Skin-Thickness Z × Geometry*	6.742	4.60	0.034	- 0.577
Curvature	1.414	0.96	0.328	- 0.270

Table S21. ANOVA results and regression coefficient values for the reduced model in Design A2, with roughness as the response, at a significance level of 95%. Significant parameters are denoted by asterisks, with the number of asterisks reflecting the statistical significance level—more asterisks indicate higher statistical significance (lower P-value).

**ANOVA: A2 - Roughness**	**Adj MS**	**F-Value**	**P-Value**	**Reg. Coeff.**
Model	26.418	23.06	0.000	26.61
Linear	11.434	9.98	0.000	
Post-Contour Power	1.154	1.01	0.317	0.00144
Skin-Energy Density**	11.636	10.16	0.002	- 6.924
Skin-Hatch Distance*	4.895	4.27	0.041	- 70.1
Skin-Stripe Overlap*	10.542	9.20	0.003	10.57
Skin-Thickness XY**	19.222	16.78	0.000	- 4.437
Skin-Thickness Z**	53.060	46.32	0.000	- 0.539

Core-Energy Density*	10.146	8.86	0.004	- 1.348
Core-Power	0.083	0.07	0.789	0.00381
Core-Stripe/Square Width	0.194	0.17	0.681	0.0261
Support Height**	16.261	14.20	0.000	0.0896
Layer Thickness	0.001	0.00	0.976	20.9
Print Location*	4.916	4.29	0.040	0.1812
Geometry**	21.691	18.94	0.000	- 1.240
2-Way Interactions	41.444	36.18	0.000	
Post-Contour Power × Skin-Energy Density***	81.929	71.52	0.000	0.00804
Post-Contour Power × Skin-Thickness XY*	4.679	4.08	0.046	0.00600
Post-Contour Power × Skin-Thickness Z**	21.345	18.63	0.000	0.01283
Post-Contour Power × Core-Power**	15.975	13.95	0.000	- 0.000044
Post-Contour Power × Layer Thickness****	126.208	110.18	0.000	- 0.6237
Skin-Energy Density × Skin-Hatch Distance**	66.854	58.36	0.000	29.05
Skin-Energy Density × Skin-Stripe Overlap*	5.339	4.66	0.033	- 2.35
Skin-Energy Density × Skin-Thickness XY***	101.815	88.88	0.000	1.793
Skin-Energy Density × Skin-Thickness Z*	5.354	4.67	0.033	0.411
Skin-Energy Density × Core-Energy Density***	101.406	88.53	0.000	0.5725
Skin-Energy Density × Layer Thickness*	8.912	7.78	0.006	10.61
Skin-Stripe Overlap × Geometry**	75.523	65.93	0.000	11.03
Core-Stripe/Square Width × Geometry*	6.610	5.77	0.018	0.1523
Support Height × Geometry*	5.329	4.65	0.033	0.0513
Print Location × Geometry*	8.487	7.41	0.007	0.2381
Curvature	11.348	9.91	0.002	0.764

5 Design B2

Table S22. Categories, types, and levels of process parameters for Design B2. Parameters with a fixed value are deemed statistically insignificant based on the ANOVA of Design B1 and, therefore, have been excluded from further study.

Category	No.	Code	Parameter	Type	Low (-1)	Mid (0)	High (+1)
**Pre-exposure**	-	-	Contour Speed [mm/s]	Fixed 1500			
	-	-	Contour Power [W]	Fixed: 30			
**Post-exposure**	-	-	Contour Speed [mm/s]	Fixed: 1500			
	1	A	Contour Power [W]	Continuous	0	80	160
**Skin-exposure**	2	B	Energy Density [J/mm^2]	Continuous	1.5	2.75	4
	-	-	Power [W]	Fixed: 150			
	-	-	Hatch Distance [mm]	Fixed: 0.08			
	-	-	Stripe Width [mm]	Fixed: 3.5			
	3	C	Stripe Overlap [mm]	Continuous	0.01	0.08	0.15
	4	D	Skin Thickness X/Y [mm]	Continuous	0.2	0.6	1
	5	E	Skin Thickness Z [mm]	Continuous	0.2	0.6	1
**Core-exposure**	6	F	Energy Density [J/mm^2]	Continuous	1.5	2.75	4
	7	G	Power [W]	Continuous	150	250	350
	8	H	Hatch Distance [mm]	Continuous	0.08	0.09	0.1
	9	J	Stripe/Square Width [mm]	Continuous	2	3.5	5
	10	K	Stripe/Square Overlap [mm]	Continuous	0.01	0.08	0.15
	-	-	Support Area Spacing [mm]	Fixed: 0.6			

**Support Structure**	11	L	Support Height [mm]	Continuous	0	4	8
**Other**	12	M	Layer Thickness [mm]	Continuous	0.02	0.04	0.06
	-	-	Angle [deg]	Fixed: 90			
	13	N	Print Location [mm]	Categorical	Section A	N/A	Section B
	14	O	Geometry	Categorical	Cube	N/A	Cylinder
	15	P	Hatching Method	Categorical	Stripe	N/A	Chess

Table S23. FFD Design B2, including only the first replicate for brevity. The second replicate and runs at center points are excluded. The second replicate shares the exact alias as the first one. For center points, all continuous parameters are fixed at 0, and the categorical ones alternate between + and -. For the parameter O (Geometry), 'Cy' denotes Cylinder, and 'C' denotes Cube. For the parameter P (Hatching Method), 'S' denotes Stripe and 'C' denotes Chess.

	**A**	**B**	**C**	**D**	**E**	**F**	**G**	**H**	**J**	**K**	**L**	**M**	**N**	**O**	**P**
TB2-01	-	-	-	-	-	-	-	-	-	+	-	-	A	Cy	C
TB2-02	+	-	-	-	-	-	+	+	+	+	+	+	B	Cy	S
TB2-03	-	+	-	-	-	-	+	+	+	-	-	-	A	Cy	S
TB2-04	+	+	-	-	-	-	-	-	-	-	+	+	B	Cy	C
TB2-05	-	-	+	-	-	-	+	-	-	-	+	-	A	C	S
TB2-06	+	-	+	-	-	-	-	+	+	-	-	+	B	C	C
TB2-07	-	+	+	-	-	-	-	+	+	+	+	-	A	C	C
TB2-08	+	+	+	-	-	-	+	-	-	+	-	+	B	C	S
TB2-09	-	-	-	+	-	-	-	+	-	-	-	+	A	C	S
TB2-10	+	-	-	+	-	-	+	-	+	-	+	-	B	C	C
TB2-11	-	+	-	+	-	-	+	-	+	+	-	+	A	C	C
TB2-12	+	+	-	+	-	-	-	+	-	+	+	-	B	C	S
TB2-13	-	-	+	+	-	-	+	+	-	+	+	+	A	Cy	C
TB2-14	+	-	+	+	-	-	-	-	+	+	-	-	B	Cy	S
TB2-15	-	+	+	+	-	-	-	-	+	-	+	+	A	Cy	S
TB2-16	+	+	+	+	-	-	+	+	-	-	-	-	B	Cy	C
TB2-17	-	-	-	-	+	-	-	-	+	-	-	-	B	C	S
TB2-18	+	-	-	-	+	-	+	+	-	-	+	+	A	C	C
TB2-19	-	+	-	-	+	-	+	+	-	+	-	-	B	C	C
TB2-20	+	+	-	-	+	-	-	-	+	+	+	+	A	C	S
TB2-21	-	-	+	-	+	-	+	-	+	+	+	-	B	Cy	C
TB2-22	+	-	+	-	+	-	-	+	-	+	-	+	A	Cy	S
TB2-23	-	+	+	-	+	-	-	+	-	-	+	-	B	Cy	S
TB2-24	+	+	+	-	+	-	+	-	+	-	-	+	A	Cy	C
TB2-25	-	-	-	+	+	-	-	+	+	+	-	+	B	Cy	C
TB2-26	+	-	-	+	+	-	+	-	-	+	+	-	A	Cy	S
TB2-27	-	+	-	+	+	-	+	-	-	-	-	+	B	Cy	S
TB2-28	+	+	-	+	+	-	-	+	+	-	+	-	A	Cy	C
TB2-29	-	-	+	+	+	-	+	+	+	-	+	+	B	C	S

TB2-30	+	-	+	+	+	-	-	-	-	-	-	-	A	C	C
TB2-31	-	+	+	+	+	-	-	-	-	+	+	+	B	C	C
TB2-32	+	+	+	+	+	-	+	+	+	+	-	-	A	C	S
TB2-33	-	-	-	-	-	+	-	-	-	+	+	+	B	C	S
TB2-34	+	-	-	-	-	+	+	+	+	+	-	-	A	C	C
TB2-35	-	+	-	-	-	+	+	+	+	-	+	+	B	C	C
TB2-36	+	+	-	-	-	+	-	-	-	-	-	-	A	C	S
TB2-37	-	-	+	-	-	+	+	-	-	-	-	+	B	Cy	C
TB2-38	+	-	+	-	-	+	-	+	+	-	+	-	A	Cy	S
TB2-39	-	+	+	-	-	+	-	+	+	+	-	+	B	Cy	S
TB2-40	+	+	+	-	-	+	+	-	-	+	+	-	A	Cy	C
TB2-41	-	-	-	+	-	+	-	+	-	-	+	-	B	Cy	C
TB2-42	+	-	-	+	-	+	+	-	+	-	-	+	A	Cy	S
TB2-43	-	+	-	+	-	+	+	-	+	+	+	-	B	Cy	S
TB2-44	+	+	-	+	-	+	-	+	-	+	-	+	A	Cy	C
TB2-45	-	-	+	+	-	+	+	+	-	+	-	-	B	C	S
TB2-46	+	-	+	+	-	+	-	-	+	+	+	+	A	C	C
TB2-47	-	+	+	+	-	+	-	-	+	-	-	-	B	C	C
TB2-48	+	+	+	+	-	+	+	+	-	-	+	+	A	C	S
TB2-49	-	-	-	-	+	+	-	-	+	-	+	+	A	Cy	C
TB2-50	+	-	-	-	+	+	+	+	-	-	-	-	B	Cy	S
TB2-51	-	+	-	-	+	+	+	+	-	+	+	+	A	Cy	S
TB2-52	+	+	-	-	+	+	-	-	+	+	-	-	B	Cy	C
TB2-53	-	-	+	-	+	+	+	-	+	+	-	+	A	C	S
TB2-54	+	-	+	-	+	+	-	+	-	+	+	-	B	C	C
TB2-55	-	+	+	-	+	+	-	+	-	-	-	+	A	C	C
TB2-56	+	+	+	-	+	+	+	-	+	-	+	-	B	C	S
TB2-57	-	-	-	+	+	+	-	+	+	+	+	-	A	C	S
TB2-58	+	-	-	+	+	+	+	-	-	+	-	+	B	C	C
TB2-59	-	+	-	+	+	+	+	-	-	-	+	-	A	C	C
TB2-60	+	+	-	+	+	+	-	+	+	-	-	+	B	C	S
TB2-61	-	-	+	+	+	+	+	+	+	-	-	-	A	Cy	C
TB2-62	+	-	+	+	+	+	-	-	-	-	+	+	B	Cy	S
TB2-63	-	+	+	+	+	+	-	-	-	+	-	-	A	Cy	S
TB2-64	+	+	+	+	+	+	+	+	+	+	+	+	B	Cy	C

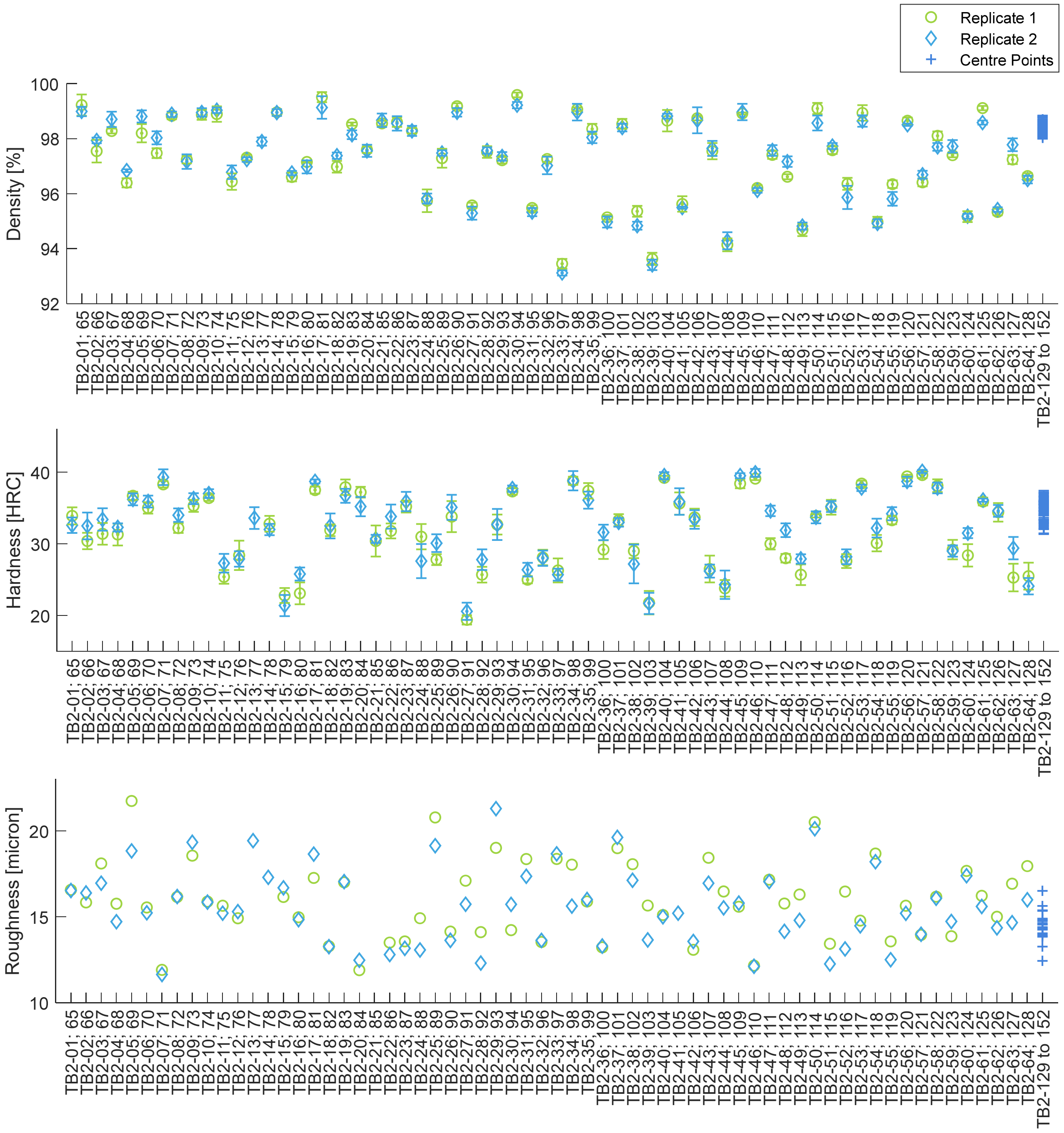

Figure S5. The measured response values for density, hardness, and roughness in Design B2. To facilitate comparison, the first and second replicates of runs sharing the same process parameters are co-located on the y-axis. The Y-axis labels, such as TB2-01; 65, indicate that the measured values correspond to parts #01 and #65 in Design B2, both printed with an identical set of process parameters.

Table S24. ANOVA results and regression coefficient values for the reduced model in Design B2, with density as the response, at a significance level of 95%. Significant parameters are denoted by asterisks, with the number of

asterisks reflecting the statistical significance level—more asterisks indicate higher statistical significance (lower P-value).

**ANOVA: B2 - Density**	**Adj MS**	**F-Value**	**P-Value**	**Reg. Coeff.**
Model	0.015459	84.04	0.000	4.3053
Linear	0.019000	103.29	0.000	
Post-Contour Power*	0.001325	7.21	0.008	0.000798
Skin-Energy Density***	0.036867	200.43	0.000	- 0.05198
Skin-Stripe Overlap*	0.000820	4.46	0.037	1.474
Skin-Thickness XY*	0.000547	2.97	0.087	0.00350
Skin-Thickness Z	0.000000	0.00	0.986	0.01957
Core-Energy Density***	0.057464	312.40	0.000	- 0.03662
Core-Power***	0.087992	478.37	0.000	0.000211
Core-Hatch Distance*	0.000741	4.03	0.047	2.117
Core-Stripe/Square Width*	0.001286	6.99	0.009	0.00787
Core-Stripe/Square Overlap	0.000024	0.13	0.717	0.160
Support Height**	0.010460	56.87	0.000	- 0.023003
Layer Thickness***	0.076832	417.70	0.000	- 0.963
Print Location**	0.006824	37.10	0.000	- 0.02902
Geometry*	0.001746	9.49	0.003	- 0.00950
Hatching Method	0.000115	0.63	0.430	0.02544
2-Way Interactions	0.011754	63.90	0.000	
Post-Contour Power × Skin-Stripe Overlap**	0.005078	27.61	0.000	- 0.001131
Post-Contour Power × Skin-Thickness XY**	0.002369	12.88	0.000	0.000135
Post-Contour Power × Core-Hatch Distance**	0.013374	72.71	0.000	- 0.01285
Post-Contour Power × Core-Stripe/Square Overlap**	0.002989	16.25	0.000	0.000868
Post-Contour Power × Support Height**	0.003902	21.21	0.000	0.000017
Post-Contour Power × Layer Thickness**	0.007166	38.96	0.000	0.004703
Post-Contour Power × Print Location**	0.004457	24.23	0.000	0.000074
Post-Contour Power × Geometry*	0.000585	3.18	0.077	0.000027
Post-Contour Power × Hatching Method**	0.001960	10.65	0.001	- 0.000049
Skin-Energy Density × Core-Energy Density**	0.015660	85.14	0.000	0.007119
Skin-Energy Density × Support Height****	0.156556	851.12	0.000	0.007034
Skin-Energy Density × Layer Thickness**	0.004356	23.68	0.000	- 0.2347
Skin-Energy Density × Print Location*	0.000841	4.57	0.035	0.002062
Skin-Stripe Overlap × Core-Hatch Distance**	0.009202	50.02	0.000	- 12.18
Skin-Stripe Overlap × Core-Stripe/Square Width**	0.007195	39.12	0.000	- 0.0718
Skin-Thickness XY × Skin-Thickness Z**	0.003428	18.64	0.000	- 0.03252
Skin-Thickness XY × Print Location*	0.000900	4.89	0.029	- 0.00667
Skin-Thickness XY × Hatching Method*	0.001497	8.14	0.005	- 0.00860
Skin-Thickness Z × Geometry*	0.000876	4.76	0.031	0.00658
Skin-Thickness Z × Hatching Method**	0.016695	90.76	0.000	- 0.02871
Core-Power × Core-Stripe/Square Overlap**	0.002709	14.73	0.000	0.000661
Core-Power × Print Location**	0.004369	23.75	0.000	0.000059
Core-Hatch Distance × Core-Stripe/Square Overlap*	0.001232	6.70	0.011	- 4.46
Curvature	0.046574	253.20	0.000	0.04805

Table S25. ANOVA results and regression coefficient values for the reduced model in Design A2, with hardness as the response, at a significance level of 95%. Significant parameters are denoted by asterisks, with the number of

asterisks reflecting the statistical significance level—more asterisks indicate higher statistical significance (lower P-value).

**ANOVA: B2 - Hardness**	**Adj MS**	**F-Value**	**P-Value**	**Reg. Coeff.**
Model	106.254	73.99	0.000	11.60
Linear	95.091	66.22	0.000	
Post-Contour Power	2.411	1.68	0.198	0.2357
Skin-Energy Density***	525.815	366.17	0.000	- 2.654
Skin-Stripe Overlap*	6.280	4.37	0.039	132.8
Skin-Thickness XY***	156.509	108.99	0.000	- 2.357
Skin-Thickness Z	0.579	0.40	0.527	1.169
Core-Energy Density**	14.370	10.01	0.002	0.2694
Core-Power**	53.144	37.01	0.000	- 0.00059
Core-Hatch Distance	1.821	1.27	0.262	355.8
Core-Stripe/Square Width	1.377	0.96	0.329	0.288
Core-Stripe/Square Overlap	0.130	0.09	0.764	- 25.97
Support Height	4.735	3.30	0.072	- 0.7219
Layer Thickness***	159.716	111.22	0.000	- 111.57
Print Location**	34.852	24.27	0.000	- 0.438
Geometry***	445.916	310.53	0.000	- 1.7201
Hatching Method	5.465	3.81	0.053	2.092
2-Way Interactions	115.795	80.64	0.000	
Post-Contour Power × Skin-Thickness XY*	6.802	4.74	0.031	0.00724
Post-Contour Power × Core-Hatch Distance****	744.887	518.73	0.000	- 3.031
Post-Contour Power × Core-Stripe/Square Overlap*	7.292	5.08	0.026	0.0428
Post-Contour Power × Support Height*	8.865	6.17	0.014	0.000827
Post-Contour Power × Layer Thickness***	155.740	108.45	0.000	0.6929
Post-Contour Power × Print Location*	8.573	5.97	0.016	0.00325
Skin-Energy Density × Support Height***	207.646	144.60	0.000	0.2560
Skin-Stripe Overlap × Core-Hatch Distance**	99.640	69.39	0.000	1267
Skin-Stripe Overlap × Core-Stripe/Square Width**	27.930	19.45	0.000	- 4.47
Skin-Stripe Overlap × Print Location*	8.911	6.21	0.014	- 3.79
Skin-Stripe Overlap × Hatching Method*	7.574	5.27	0.023	3.49
Skin-Thickness XY × Skin-Thickness Z*	9.017	6.28	0.014	- 1.667
Skin-Thickness Z × Hatching Method***	267.980	186.62	0.000	- 3.636
Core-Power × Core-Stripe/Square Overlap**	48.445	33.74	0.000	0.0883
Curvature	136.177	94.83	0.000	2.598

Table S26. ANOVA results and regression coefficient values for the reduced model in Design A2, with roughness as the response, at a significance level of 95%. Significant parameters are denoted by asterisks, with the number of asterisks reflecting the statistical significance level—more asterisks indicate higher statistical significance (lower P-value).

**ANOVA: B2 - Roughness**	**Adj MS**	**F-Value**	**P-Value**	**Reg. Coeff.**
Model	34.850	17.17	0.000	24.05
Linear	30.986	15.26	0.000	
Post-Contour Power***	67.738	33.37	0.000	- 0.1120
Skin-Energy Density***	80.314	39.56	0.000	- 1.946

Skin-Stripe Overlap	1.921	0.95	0.332	69.7
Skin-Thickness Z	1.141	0.56	0.455	- 0.239
Core-Energy Density*	6.435	3.17	0.077	- 1.526
Core-Power**	21.484	10.58	0.001	0.01114
Core-Hatch Distance	1.722	0.85	0.359	- 22.5
Layer Thickness	5.437	2.68	0.104	- 9.4
Print Location****	122.629	60.41	0.000	0.909
Hatching Method	0.509	0.25	0.617	- 0.623
2-Way Interactions	37.612	18.53	0.000	
Post-Contour Power × Core-Energy Density***	84.960	41.85	0.000	0.00826
Post-Contour Power × Core-Hatch Distance***	63.133	31.10	0.000	0.890
Skin-Energy Density × Core-Energy Density**	18.797	9.26	0.003	0.2485
Skin-Energy Density × Layer Thickness**	18.712	9.22	0.003	15.51
Skin-Stripe Overlap × Core-Hatch Distance**	34.796	17.14	0.000	- 755
Skin-Thickness Z × Hatching Method**	25.701	12.66	0.001	1.135
Core-Power × Layer Thickness**	15.202	7.49	0.007	- 0.1747
Curvature	43.324	21.34	0.000	- 1.467

6 Design C2

Table S27. Categories, types, and levels of process parameters for Design C2. Parameters with a fixed value are deemed statistically insignificant based on the ANOVA of Design C1 and, therefore, have been excluded from further study.

Category	No.	Code	Parameter	Type	Low (-1)	Mid (0)	High (+1)
**Pre-exposure**	1	-	Contour Speed [mm/s]	Fixed 1100			
	2	A	Contour Power [W]	Continuous	10	35	60
**Post-exposure**	3	-	Contour Speed [mm/s]	Fixed 1100			
	4	B	Contour Power [W]	Continuous	10	85	160
**Skin-exposure**	5	C	Specific Energy** [J/mm^2]	Continuous	1.5	2.75	4
	6	D	Power [W]	Continuous	150	250	350
	7	-	Hatch Distance [mm]	Fixed 0.1			
	8	-	Stripe Width [mm]	Fixed 3.5			
	9	E	Stripe Overlap [mm]	Continuous	0.01	0.08	0.15
	10	-	Skin Thickness X/Y [mm]	Fixed 0.6			
	11	F	Skin Thickness Z [mm]	Continuous	0.2	0.6	1
**Core-exposure**	12	G	Specific Energy** [J/mm^2]	Continuous	1.5	2.75	4
	13	H	Power [W]	Continuous	150	250	350
	14	J	Hatch Distance [mm]	Continuous	0.08	0.09	0.1
	15	-	Stripe/Square Width [mm]	Fixed 3.5			
	16	-	Stripe/Square Overlap [mm]	Fixed 0.08			
**Support Structure**	17	K	Support Area Spacing [mm]	Continuous	0.6	0.7	0.8
	18	L	Support Height [mm]	Continuous	2	5	8
**Other**	19	M	Layer Thickness [mm]	Continuous	0.02	0.04	0.06
	-	-	Angle [deg]	Fixed: 90			
	21	-	Print Location [mm]	Not significant			
	-	-	Geometry	Fixed: Cube			
	23	N	Hatching Method	Categorical	Stripe	N/A	Chess

Table S28. FFD Design C2, including only the first replicate for brevity. The second replicate and runs at center points are excluded. The second replicate shares the exact alias as the first one. For center points, all continuous parameters are fixed at 0, and the categorical ones alternate between + and -. For the parameter N (Hatching Method), 'S' denotes Stripe and 'C' denotes Chess.

	A	B	C	D	E	F	G	H	J	K	L	M	N
TC2-01	-	-	-	-	-	-	-	-	+	+	+	+	C
TC2-02	+	-	-	-	-	-	+	-	+	+	-	-	C
TC2-03	-	+	-	-	-	-	+	-	-	-	-	+	S
TC2-04	+	+	-	-	-	-	-	-	-	-	+	-	S
TC2-05	-	-	+	-	-	-	+	-	-	-	+	-	S
TC2-06	+	-	+	-	-	-	-	-	-	-	-	+	S
TC2-07	-	+	+	-	-	-	-	-	+	+	-	-	C
TC2-08	+	+	+	-	-	-	+	-	+	+	+	+	C
TC2-09	-	-	-	+	-	-	-	+	-	-	+	+	C
TC2-10	+	-	-	+	-	-	+	+	-	-	-	-	C
TC2-11	-	+	-	+	-	-	+	+	+	+	-	+	S
TC2-12	+	+	-	+	-	-	-	+	+	+	+	-	S
TC2-13	-	-	+	+	-	-	+	+	+	+	+	-	S
TC2-14	+	-	+	+	-	-	-	+	+	+	-	+	S
TC2-15	-	+	+	+	-	-	-	+	-	-	-	-	C
TC2-16	+	+	+	+	-	-	+	+	-	-	+	+	C
TC2-17	-	-	-	-	+	-	-	+	+	-	-	-	S
TC2-18	+	-	-	-	+	-	+	+	+	-	+	+	S
TC2-19	-	+	-	-	+	-	+	+	-	+	+	-	C
TC2-20	+	+	-	-	+	-	-	+	-	+	-	+	C
TC2-21	-	-	+	-	+	-	+	+	-	+	-	+	C
TC2-22	+	-	+	-	+	-	-	+	-	+	+	-	C
TC2-23	-	+	+	-	+	-	-	+	+	-	+	+	S
TC2-24	+	+	+	-	+	-	+	+	+	-	-	-	S
TC2-25	-	-	-	+	+	-	-	-	-	+	-	-	S
TC2-26	+	-	-	+	+	-	+	-	-	+	+	+	S
TC2-27	-	+	-	+	+	-	+	-	+	-	+	-	C
TC2-28	+	+	-	+	+	-	-	-	+	-	-	+	C
TC2-29	-	-	+	+	+	-	+	-	+	-	-	+	C
TC2-30	+	-	+	+	+	-	-	-	+	-	+	-	C
TC2-31	-	+	+	+	+	-	-	-	-	+	+	+	S
TC2-32	+	+	+	+	+	-	+	-	-	+	-	-	S
TC2-33	-	-	-	-	-	+	-	+	-	+	-	-	S
TC2-34	+	-	-	-	-	+	+	+	-	+	+	+	S
TC2-35	-	+	-	-	-	+	+	+	+	-	+	-	C

TC2-36	+	+	-	-	-	+	-	+	+	-	-	+	C
TC2-37	-	-	+	-	-	+	+	+	+	-	-	+	C
TC2-38	+	-	+	-	-	+	-	+	+	-	+	-	C
TC2-39	-	+	+	-	-	+	-	+	-	+	+	+	S
TC2-40	+	+	+	-	-	+	+	+	-	+	-	-	S
TC2-41	-	-	-	+	-	+	-	-	+	-	-	-	S
TC2-42	+	-	-	+	-	+	+	-	+	-	+	+	S
TC2-43	-	+	-	+	-	+	+	-	-	+	+	-	C
TC2-44	+	+	-	+	-	+	-	-	-	+	-	+	C
TC2-45	-	-	+	+	-	+	+	-	-	+	-	+	C
TC2-46	+	-	+	+	-	+	-	-	-	+	+	-	C
TC2-47	-	+	+	+	-	+	-	-	+	-	+	+	S
TC2-48	+	+	+	+	-	+	+	-	+	-	-	-	S
TC2-49	-	-	-	-	+	+	-	-	-	-	+	+	C
TC2-50	+	-	-	-	+	+	+	-	-	-	-	-	C
TC2-51	-	+	-	-	+	+	+	-	+	+	-	+	S
TC2-52	+	+	-	-	+	+	-	-	+	+	+	-	S
TC2-53	-	-	+	-	+	+	+	-	+	+	+	-	S
TC2-54	+	-	+	-	+	+	-	-	+	+	-	+	S
TC2-55	-	+	+	-	+	+	-	-	-	-	-	-	C
TC2-56	+	+	+	-	+	+	+	-	-	-	+	+	C
TC2-57	-	-	-	+	+	+	-	+	+	+	+	+	C
TC2-58	+	-	-	+	+	+	+	+	+	+	-	-	C
TC2-59	-	+	-	+	+	+	+	+	-	-	-	+	S
TC2-60	+	+	-	+	+	+	-	+	-	-	+	-	S
TC2-61	-	-	+	+	+	+	+	+	-	-	+	-	S
TC2-62	+	-	+	+	+	+	-	+	-	-	-	+	S
TC2-63	-	+	+	+	+	+	-	+	+	+	-	-	C
TC2-64	+	+	+	+	+	+	+	+	+	+	+	+	C

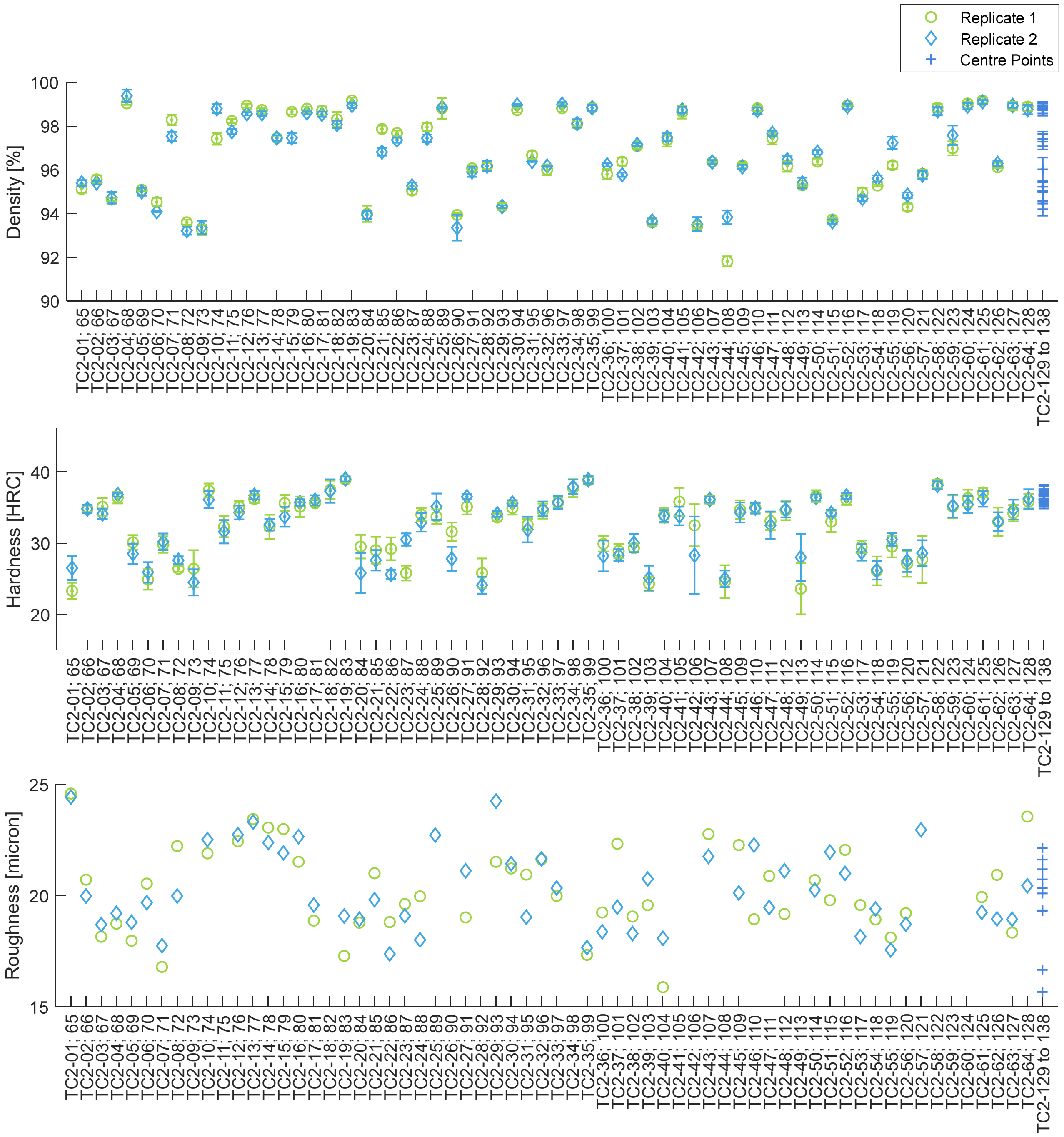

Figure S6. The measured response values for density, hardness, and roughness in Design C2. To facilitate comparison, the first and second replicates of runs sharing the same process parameters are co-located on the y-axis. The Y-axis labels, such as TC2-01; 65, indicate that the measured values correspond to parts #01 and #56 in Design C2, both printed with an identical set of process parameters.

Table S29. ANOVA results and regression coefficient values for the reduced model in Design C2, with density as the response, at a significance level of 95%. Significant parameters are denoted by asterisks, with the number of

asterisks reflecting the statistical significance level—more asterisks indicate higher statistical significance (lower P-value).

ANOVA: C2 - Density	Adj MS	F-Value	P-Value	Reg. Coeff.
Model	0.036710	40.42	0.000	4.4115
Linear	0.041659	45.87	0.000	
Pre-Contour Power	0.000085	0.09	0.760	- 0.000095
Post-Contour Power	0.000209	0.23	0.633	- 0.000215
Skin-Energy Density	0.000075	0.08	0.774	- 0.04532
Skin-Power**	0.025535	28.12	0.000	- 0.000417
Core-Energy Density*	0.004830	5.32	0.023	0.0188
Core-Power***	0.133487	146.98	0.000	- 0.000563
Core-Hatch Distance*	0.003699	4.07	0.046	1.629
Support Area Spacing	0.000000	0.00	0.982	0.2171
Support Height	0.001218	1.34	0.249	- 0.01479
Layer Thickness****	0.281544	310.00	0.000	- 5.03
Hatching Method*	0.007572	8.34	0.005	- 0.00741
2-Way Interactions	0.034936	38.47	0.000	
Pre-Contour Power × Core-Power*	0.004876	5.37	0.022	0.000002
Pre-Contour Power × Support Area Spacing*	0.006293	6.93	0.010	0.00280
Pre-Contour Power × Support Height**	0.062594	68.92	0.000	0.000295
Post-Contour Power × Skin-Power*	0.004379	4.82	0.030	0.000001
Post-Contour Power × Core-Hatch Distance*	0.004328	4.77	0.031	0.00775
Post-Contour Power × Support Area Spacing**	0.014233	15.67	0.000	- 0.001406
Post-Contour Power × Support Height**	0.027078	29.81	0.000	0.000065
Skin-Energy Density × Skin-Power**	0.063948	70.41	0.000	0.000179
Core-Energy Density × Core-Power***	0.169040	186.13	0.000	0.000291
Core-Energy Density × Core-Hatch Distance**	0.022954	25.27	0.000	- 1.071
Core-Hatch Distance × Layer Thickness*	0.004572	5.03	0.027	29.9
Curvature	0.001771	1.95	0.165	0.01382

Table S30. ANOVA results and regression coefficient values for the reduced model in Design C2, with hardness as the response, at a significance level of 95%. Significant parameters are denoted by asterisks, with the number of asterisks reflecting the statistical significance level—more asterisks indicate higher statistical significance (lower P-value).

ANOVA: C2 - Hardness	Adj MS	F-Value	P-Value	Reg. Coeff.
Model	90.571	80.35	0.000	39.12
Linear	115.275	102.26	0.000	
Pre-Contour Power	0.578	0.51	0.476	- 0.0417
Post-Contour Power*	8.715	7.73	0.006	0.00488
Skin-Energy Density**	81.920	72.67	0.000	- 3.874
Skin-Power***	137.780	122.22	0.000	- 0.02007
Skin-Thickness Z	7.125	6.32	0.013	0.590
Core-Energy Density***	308.761	273.90	0.000	2.184
Core-Power**	75.953	67.38	0.000	- 0.00865
Core-Hatch Distance	0.405	0.36	0.550	55.1
Support Height	0.070	0.06	0.803	- 0.7452

Layer Thickness****	589.961	523.35	0.000	- 132.9
Hatching Method**	56.755	50.35	0.000	- 1.524
2-Way Interactions	64.535	57.25	0.000	
Pre-Contour Power × Post-Contour Power**	59.951	53.18	0.000	- 0.000365
Pre-Contour Power × Skin-Power*	7.703	6.83	0.010	- 0.000098
Pre-Contour Power × Core-Power**	26.281	23.31	0.000	0.000181
Pre-Contour Power × Support Height**	85.805	76.12	0.000	0.01092
Post-Contour Power × Skin-Power*	10.465	9.28	0.003	- 0.000038
Post-Contour Power × Support Height***	113.251	100.47	0.000	0.004181
Skin-Energy Density × Skin-Power****	456.020	404.54	0.000	0.015100
Skin-Energy Density × Core-Power*	9.353	8.30	0.005	- 0.002162
Skin-Power × Core-Energy Density*	5.120	4.54	0.035	- 0.001600
Skin-Power × Hatching Method**	15.961	14.16	0.000	0.003531
Core-Energy Density × Core-Power**	37.195	33.00	0.000	0.004313
Core-Energy Density × Core-Hatch Distance*	6.480	5.75	0.018	- 18.00
Core-Power × Layer Thickness*	5.363	4.76	0.031	0.1023
Curvature	157.300	139.54	0.000	4.118

Table S31. ANOVA results and regression coefficient values for the reduced model in Design C2, with roughness as the response, at a significance level of 95%. Significant parameters are denoted by asterisks, with the number of asterisks reflecting the statistical significance level—more asterisks indicate higher statistical significance (lower P-value).

**ANOVA: C2 - Roughness**	**Adj MS**	**F-Value**	**P-Value**	**Reg. Coeff.**
Model	60.800	40.28	0.000	16.00
Linear	97.399	64.53	0.000	
Pre-Contour Power**	22.502	14.91	0.000	0.0120
Post-Contour Power**	30.250	20.04	0.000	0.0314
Skin-Energy Density****	469.572	311.10	0.000	1.266
Skin-Power****	489.699	324.43	0.000	0.04080
Skin-Stripe Overlap	0.400	0.26	0.608	69.0
Skin-Thickness Z	1.842	1.22	0.272	3.770
Core-Energy Density	2.405	1.59	0.210	- 6.22
Core-Power	1.466	0.97	0.327	- 0.01458
Core-Hatch Distance	0.683	0.45	0.503	- 42.7
Support Area Spacing	1.075	0.71	0.401	0.85
Support Height	0.181	0.12	0.730	0.745
Layer Thickness***	235.877	156.27	0.000	67.87
Hatching Method*	10.237	6.78	0.011	- 0.272
2-Way Interactions	34.943	23.15	0.000	
Pre-Contour Power × Skin-Energy Density**	19.083	12.64	0.001	- 0.01236
Pre-Contour Power × Skin-Power*	11.727	7.77	0.006	0.000121
Pre-Contour Power × Skin-Stripe Overlap*	7.702	5.10	0.026	0.1402
Pre-Contour Power × Core-Energy Density*	6.232	4.13	0.045	0.00706
Pre-Contour Power × Core-Power**	30.206	20.01	0.000	- 0.000194
Pre-Contour Power × Support Height**	20.105	13.32	0.000	0.00528
Post-Contour Power × Skin-Power**	47.866	31.71	0.000	0.000082
Post-Contour Power × Core-Hatch Distance*	14.791	9.80	0.002	- 0.453
Post-Contour Power × Support Height**	79.009	52.34	0.000	- 0.003492

Skin-Energy Density × Skin-Power**	105.799	70.09	0.000	- 0.007273
Skin-Energy Density × Skin-Stripe Overlap*	13.671	9.06	0.003	- 3.73
Skin-Energy Density × Skin-Thickness Z**	50.966	33.77	0.000	- 1.262
Skin-Energy Density × Core-Power*	8.264	5.48	0.021	0.002033
Skin-Power × Support Height**	70.985	47.03	0.000	- 0.002482
Skin-Stripe Overlap × Support Area Spacing**	50.614	33.53	0.000	- 89.8
Core-Energy Density × Core-Power**	57.341	37.99	0.000	0.005355
Core-Energy Density × Core-Hatch Distance**	20.706	13.72	0.000	32.18
Core-Energy Density × Support Area Spacing*	13.905	9.21	0.003	2.637
Curvature	50.446	33.42	0.000	- 2.332

7 Model diagnostics

Table S32. The statistical metrics indicating the goodness of fit for the studied designs: standard error of regression (S), R-squared, adjusted R-squared, and predicted R-squared. Note that no significant process parameter was identified for roughness in Design B1, resulting in low goodness-of-fit indicators for this design.

Design	Response	S	R-sq	Adjusted R-sq	Predicted R-sq
A1	Density	0.0134640	97.04%	95.55%	94.00%
	Hardness	1.30991	95.55%	93.32%	90.31%
	Roughness	1.82773	65.99%	57.30%	41.99%
B1	Density	0.0155210	96.76%	95.43%	93.86%
	Hardness	1.56622	93.35%	90.37%	85.13%
	Roughness	2.93203	48.52%	8.66%	0.00%
C1	Density	0.0424133	78.48%	74.82%	69.27%
	Hardness	2.66866	73.92%	70.12%	64.27%
	Roughness	1.21308	91.29%	88.02%	82.18%
A2	Density	0.0122335	96.86%	96.00%	94.81%
	Hardness	1.21126	95.36%	94.38%	93.44%
	Roughness	1.07026	84.79%	81.11%	75.27%
B2	Density	0.0135625	96.72%	95.57%	93.53%
	Hardness	1.19833	94.87%	93.59%	92.02%
	Roughness	1.42477	70.39%	66.29%	60.46%
C2	Density	0.0301363	89.08%	86.87%	85.14%
	Hardness	1.06173	94.72%	93.54%	91.91%
	Roughness	1.22858	92.47%	90.17%	87.53%

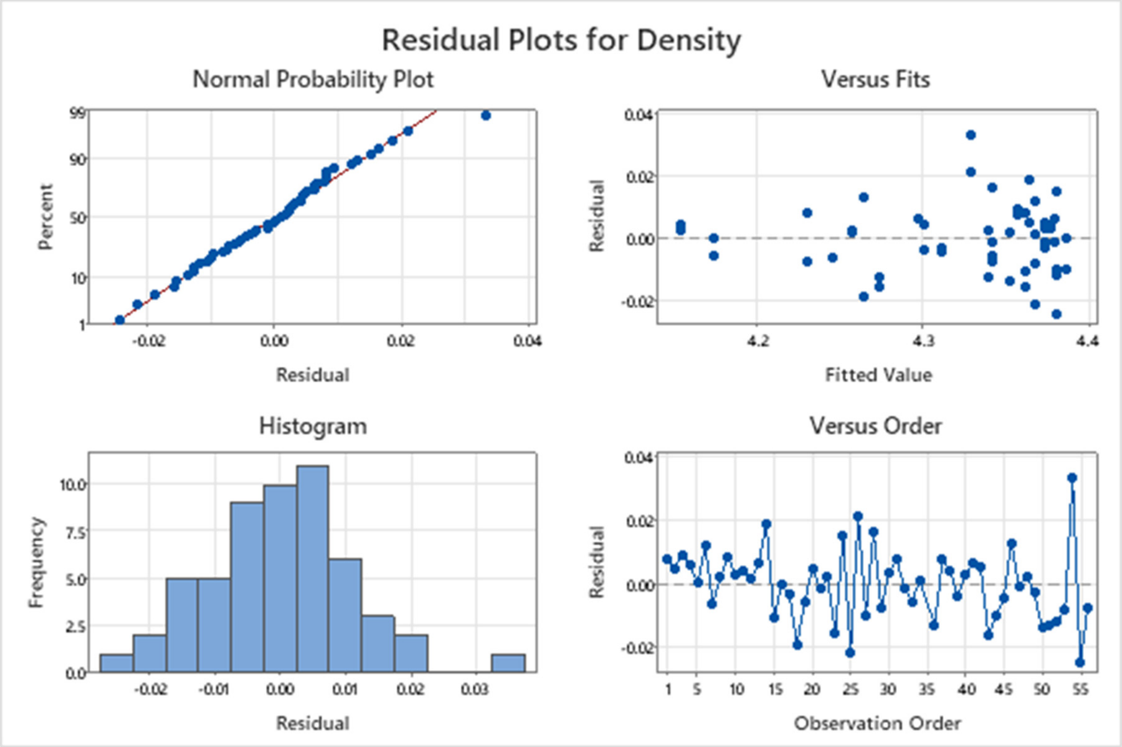

Figure S7. Residual plots for Design A1 with density as response.

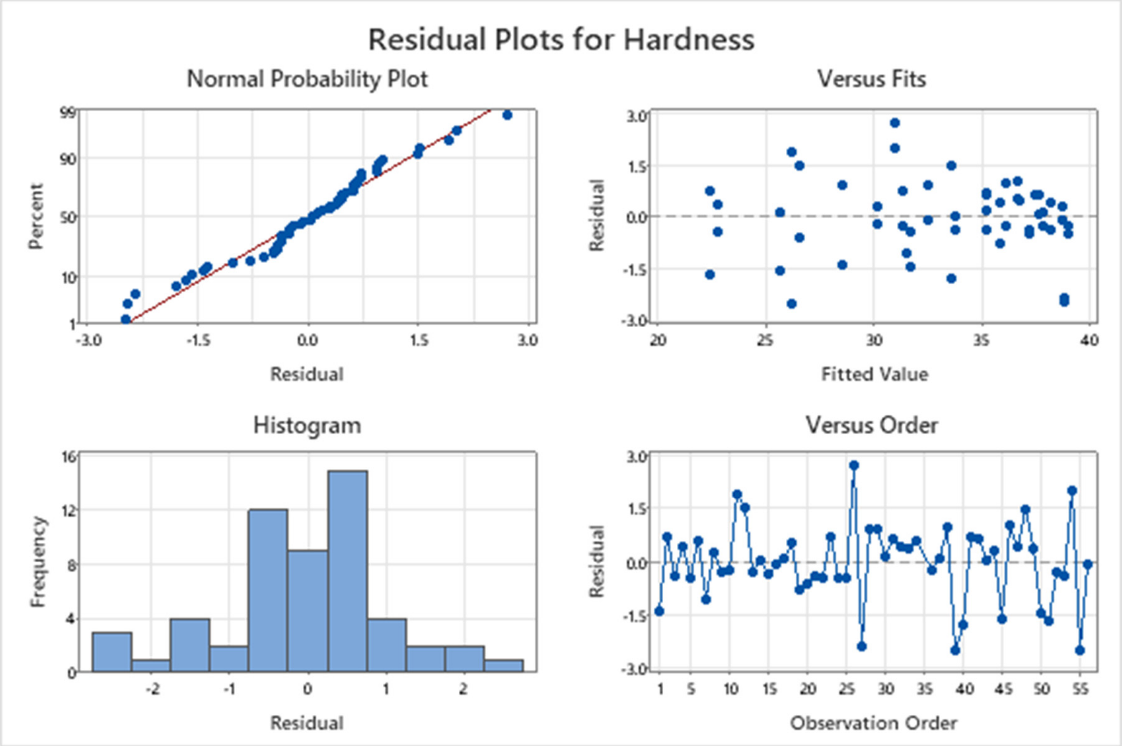

Figure S8. Residual plots for Design A1 with hardness as response.

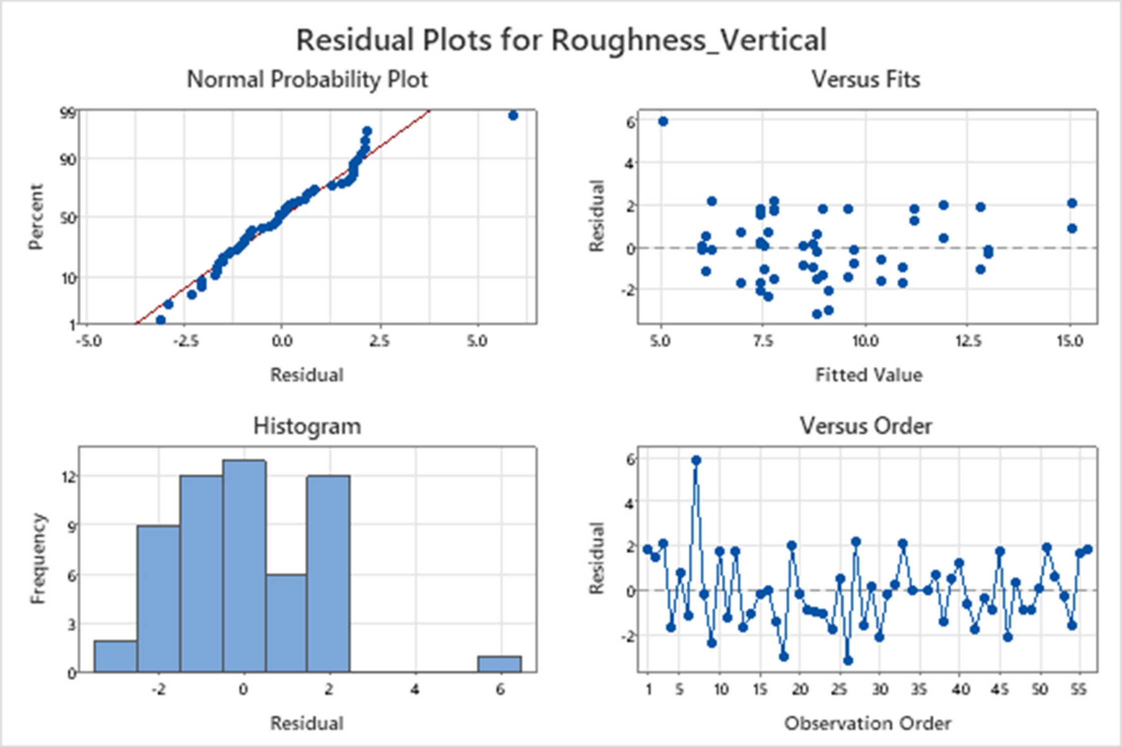

Figure S9. Residual plots for Design A1 with roughness as response.

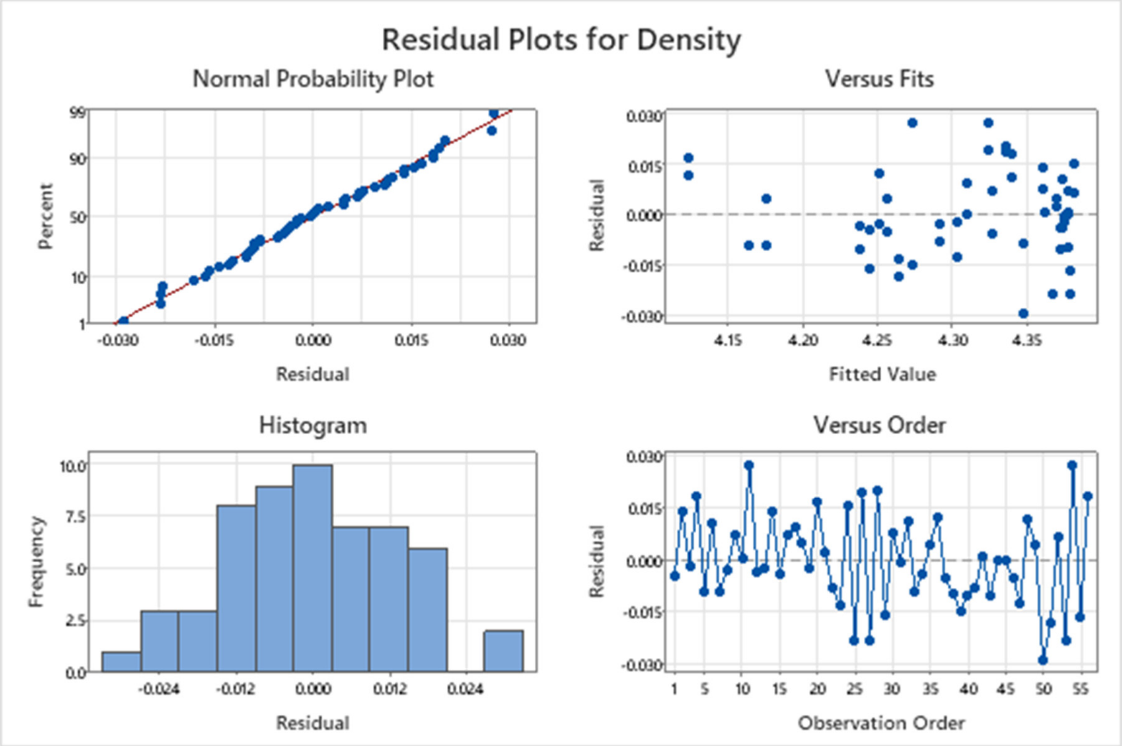

Figure S10. Residual plots for Design B1 with density as response.

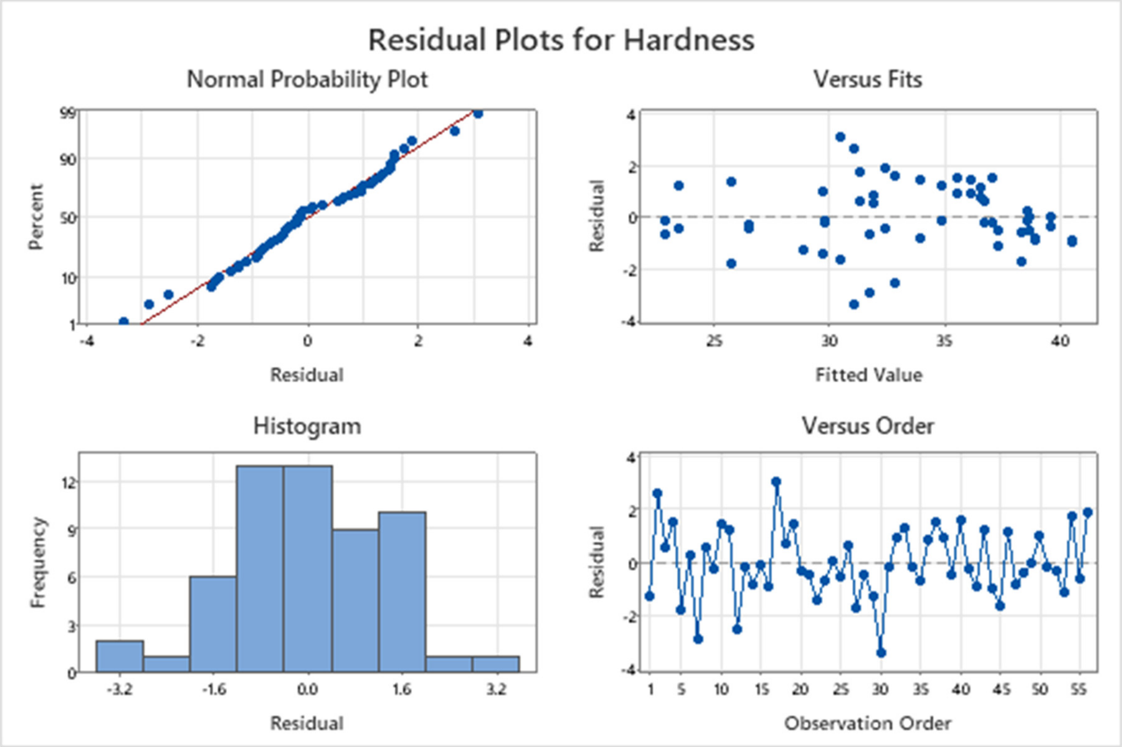

Figure S11. Residual plots for Design B1 with hardness as response.

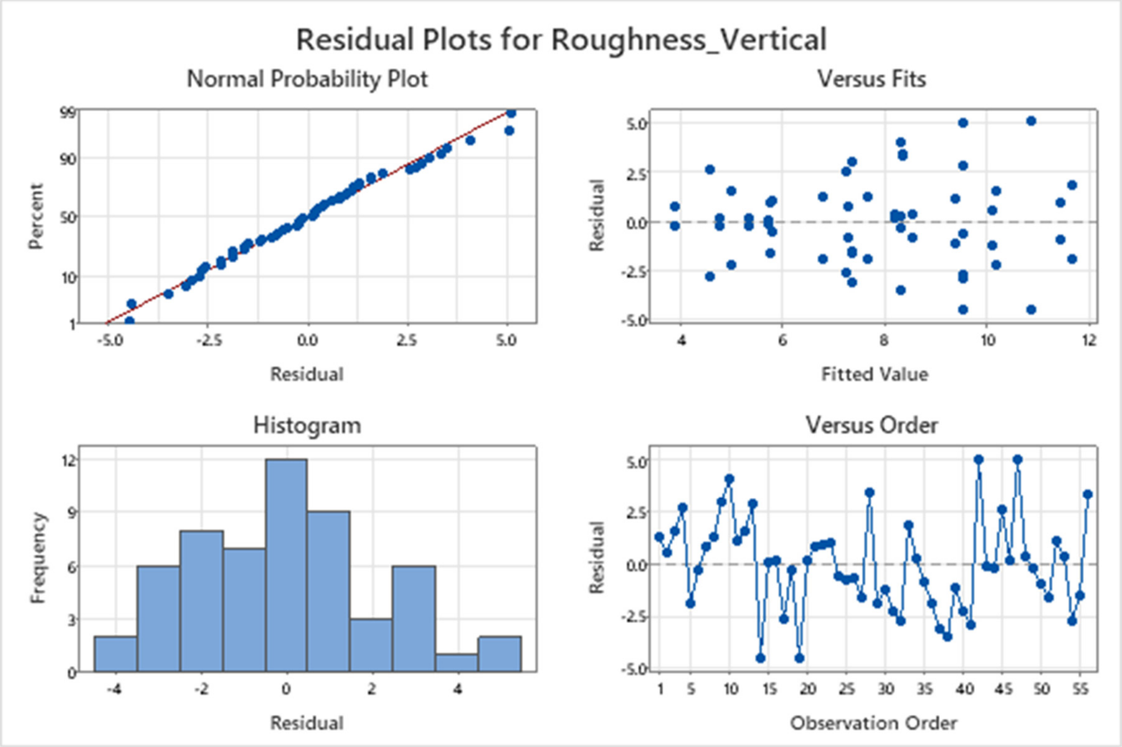

Figure S12. Residual plots for Design B1 with roughness as response.

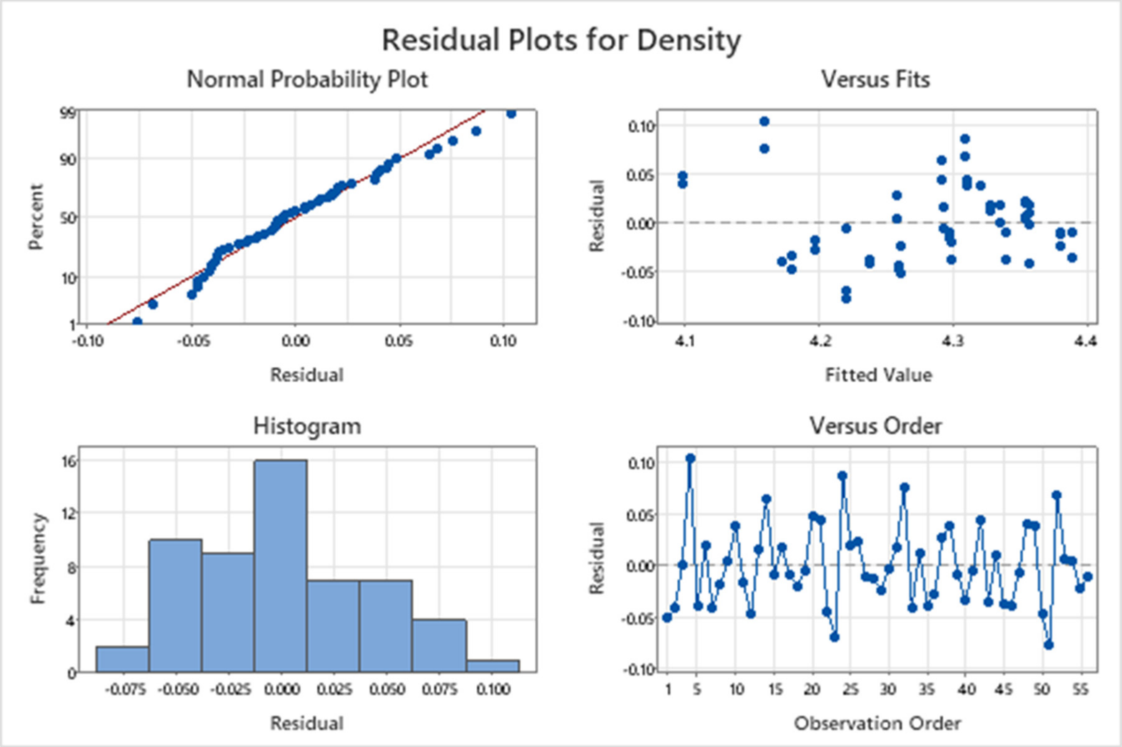

Figure S13. Residual plots for Design C1 with density as response.

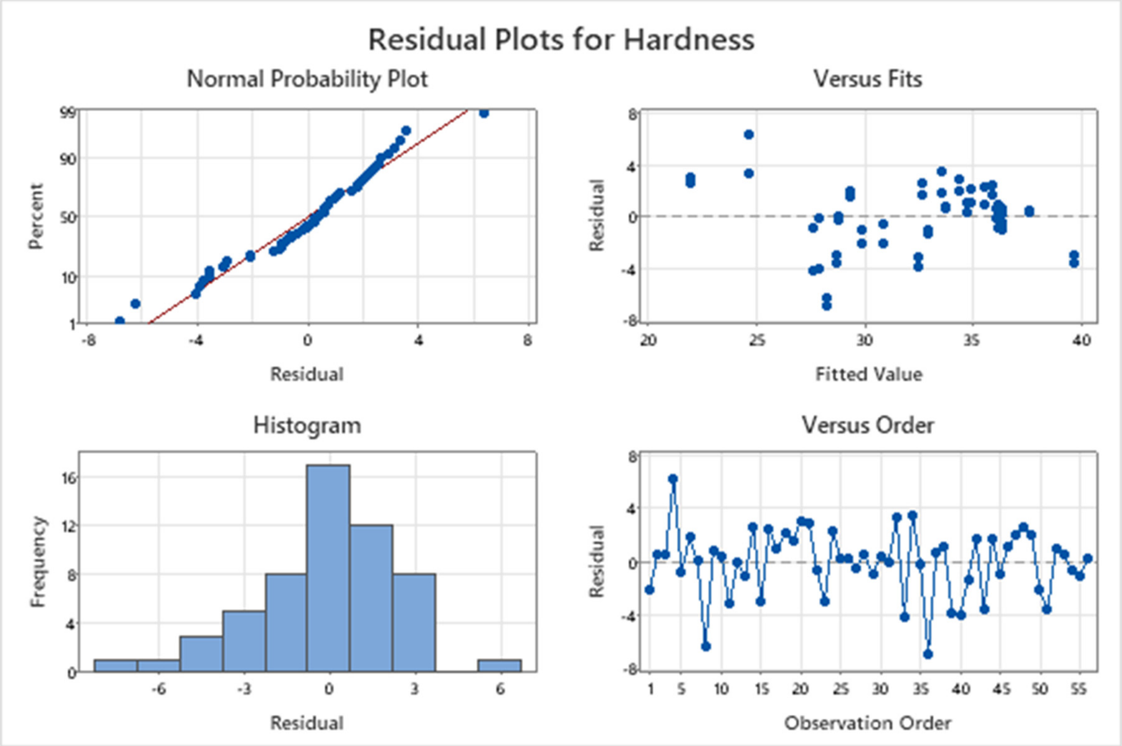

Figure S14. Residual plots for Design C1 with hardness as response.

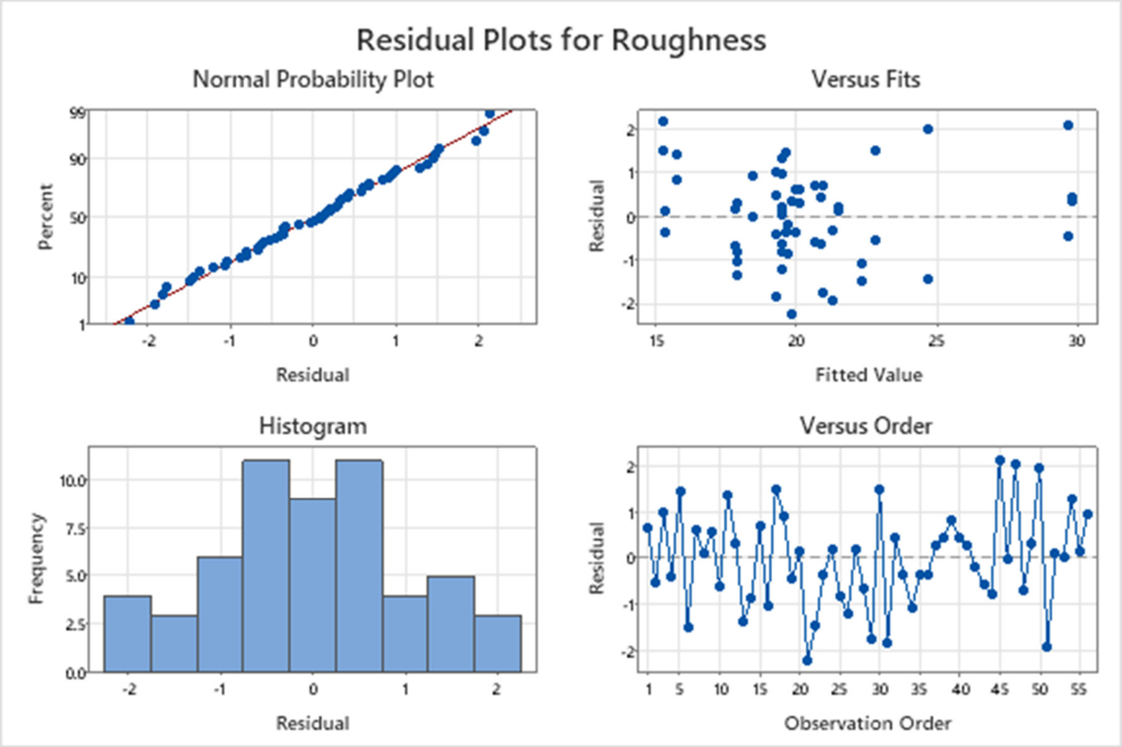

Figure S15. Residual plots for Design C1 with roughness as response.

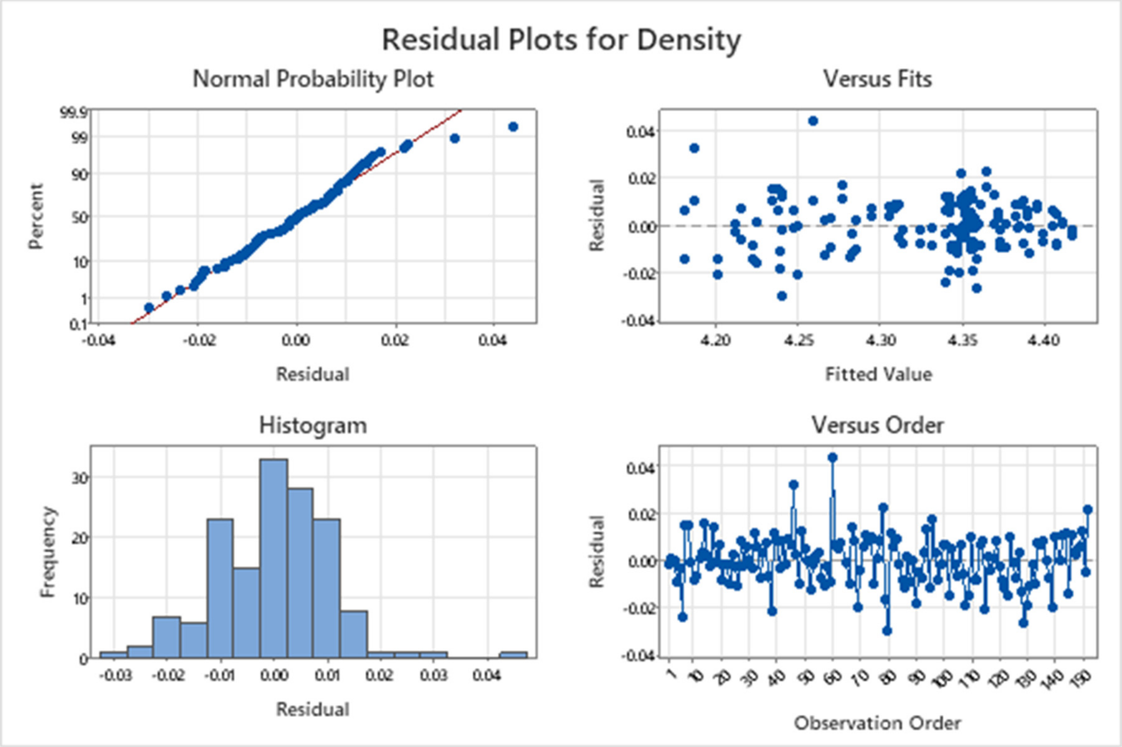

Figure S16. Residual plots for Design A2 with density as response.

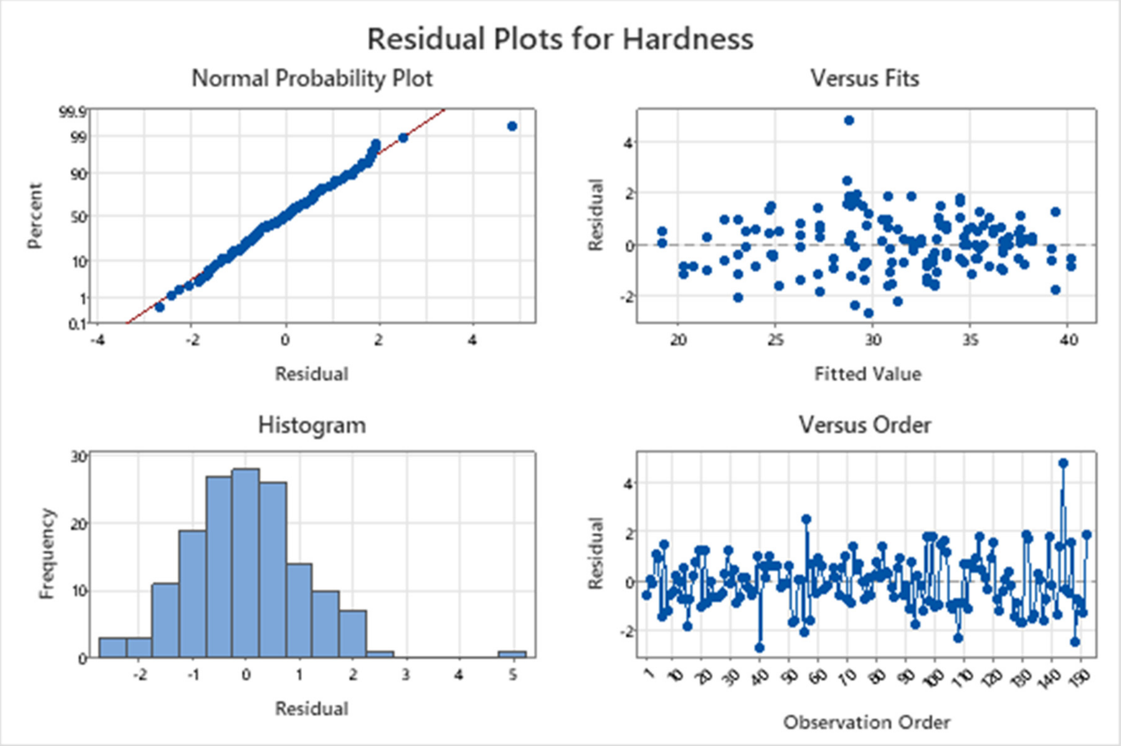

Figure S17. Residual plots for Design A2 with hardness as response.

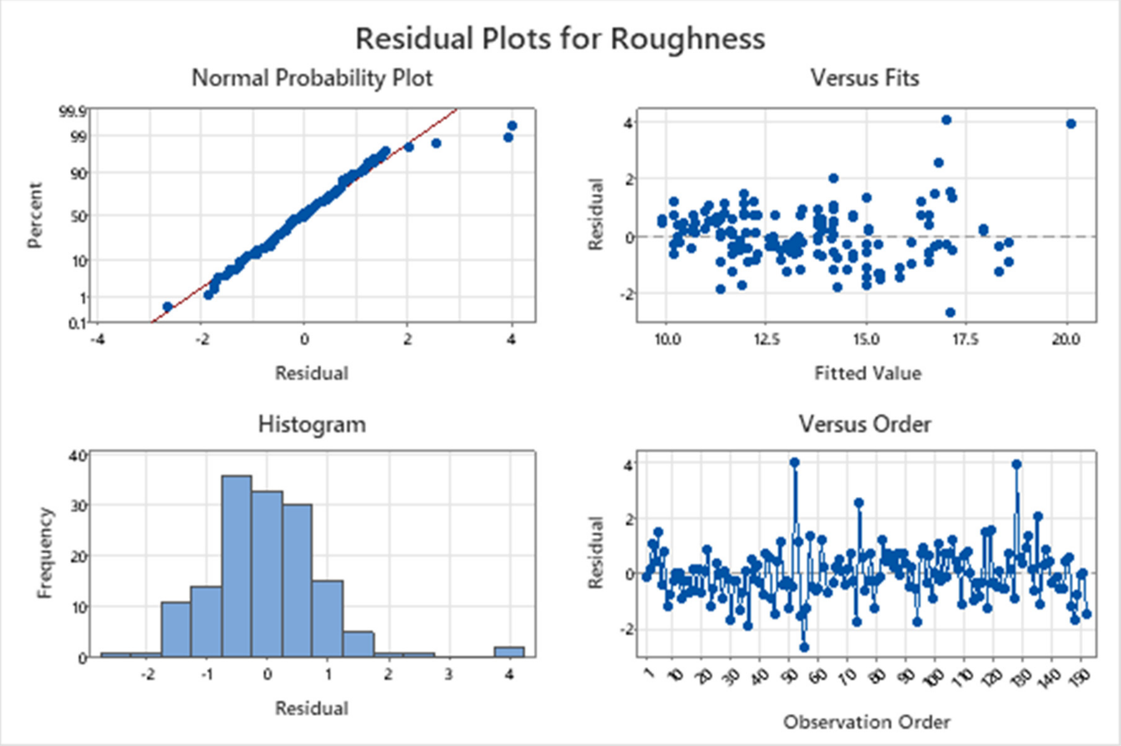

Figure S18. Residual plots for Design A2 with roughness as response.

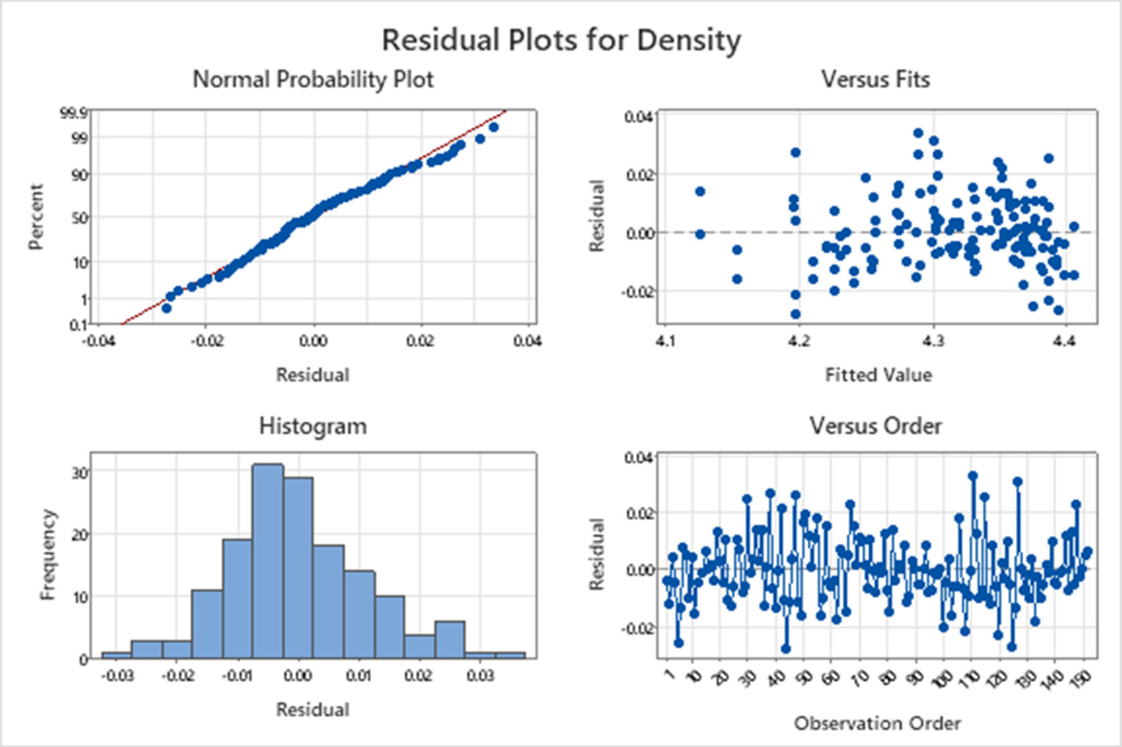

Figure S19. Residual plots for Design B2 with density as response.

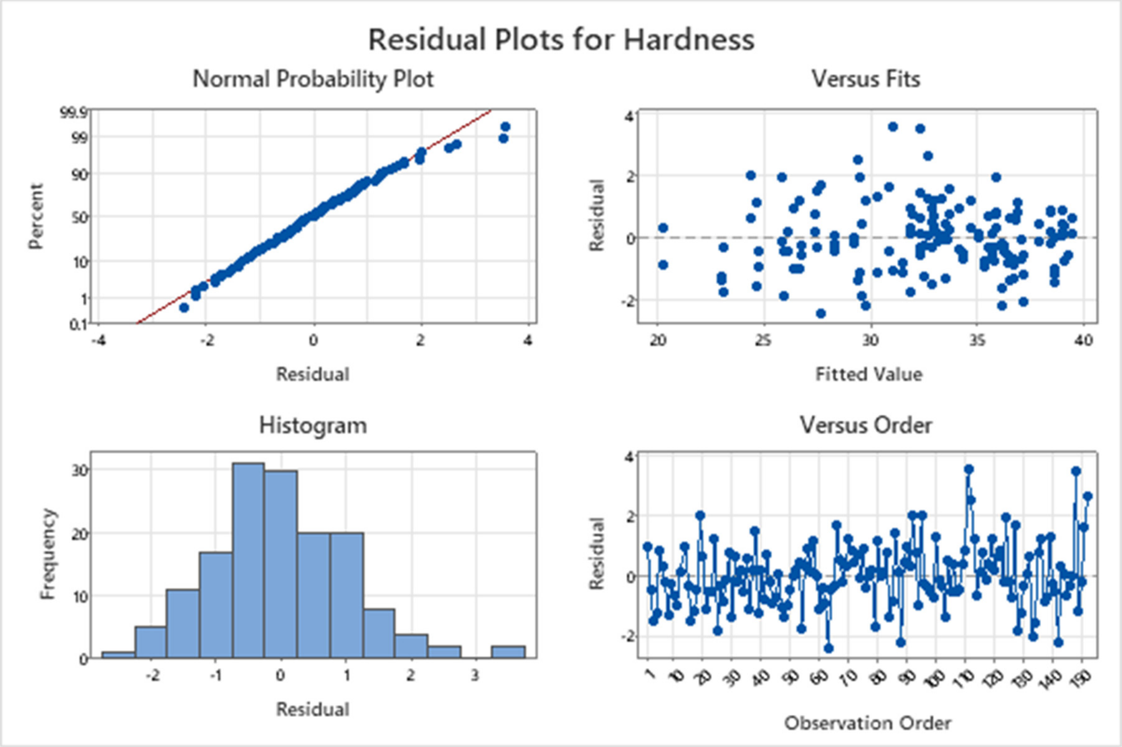

Figure S20. Residual plots for Design B2 with hardness as response.

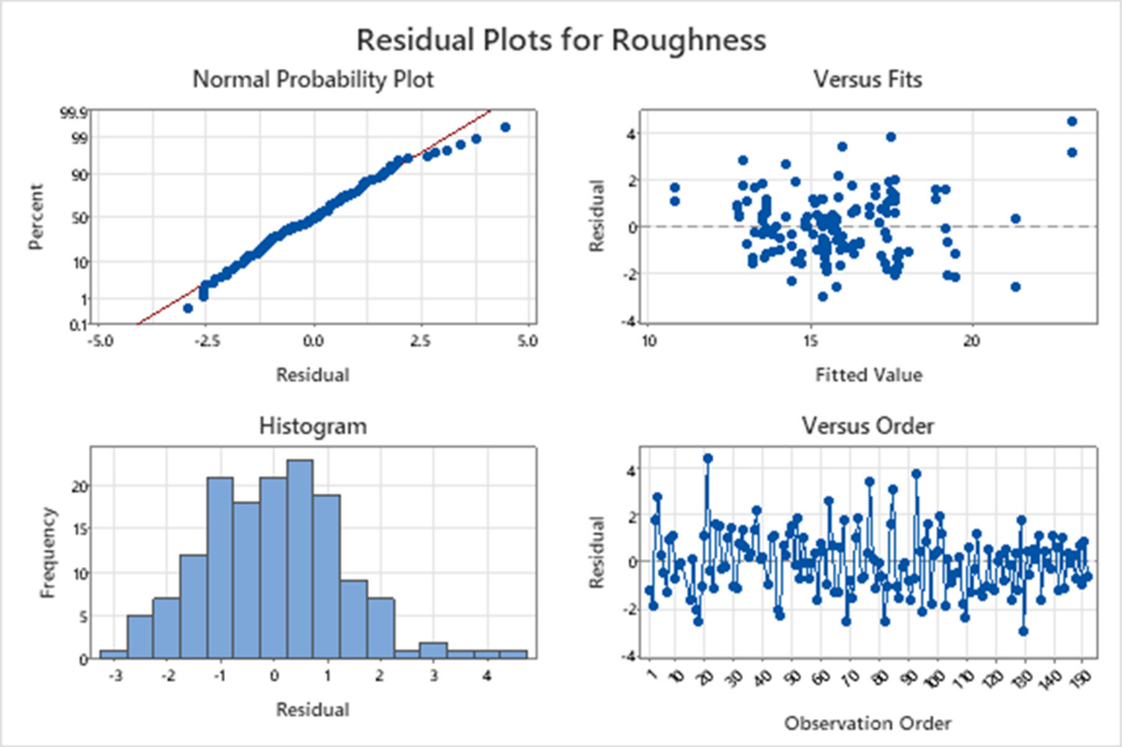

Figure S21. Residual plots for Design B2 with roughness as response.

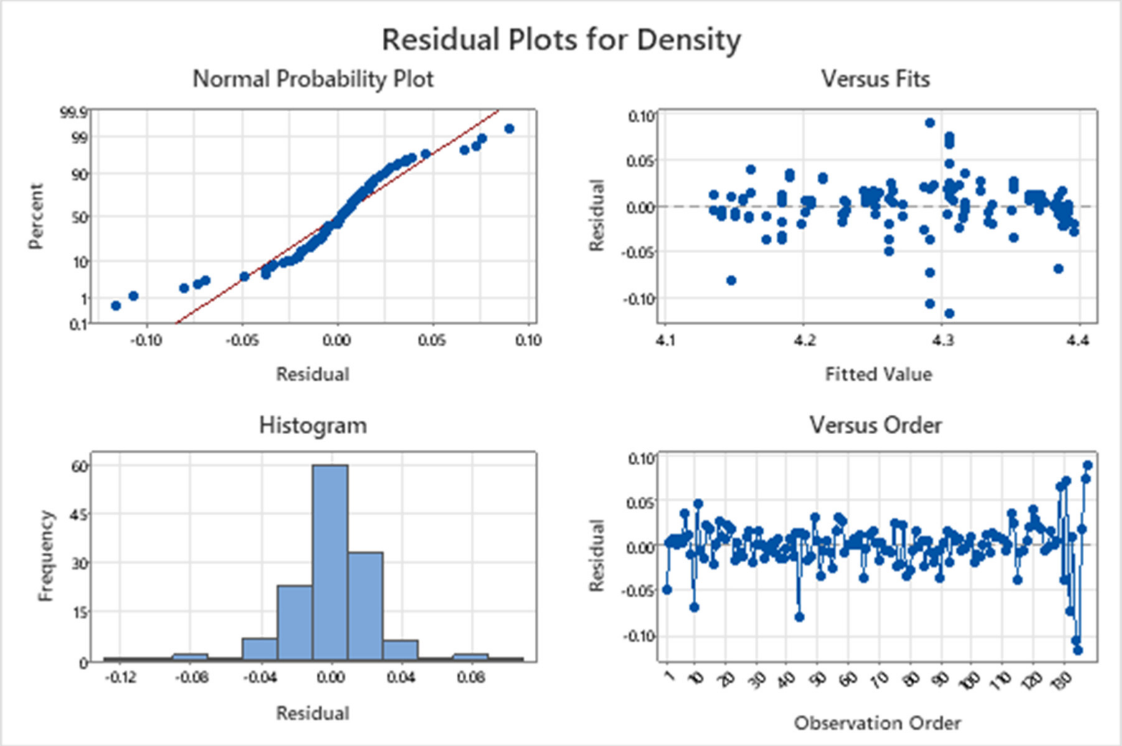

Figure S22. Residual plots for Design C2 with density as response.

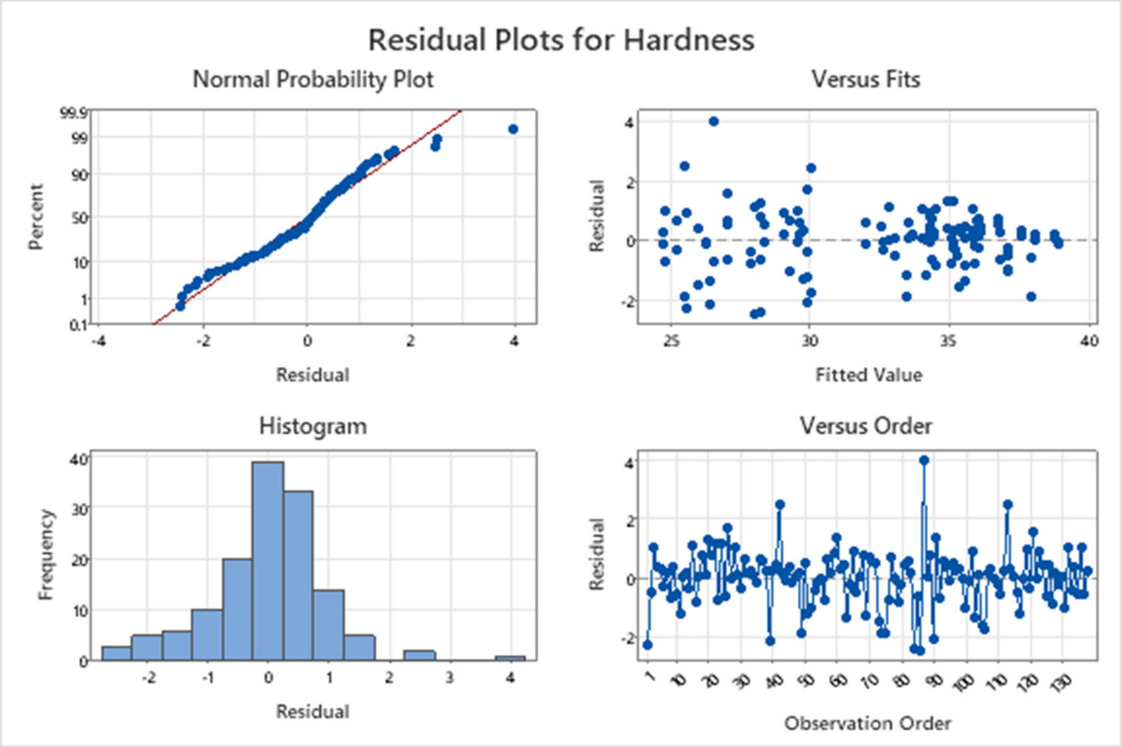

Figure S23. Residual plots for Design C2 with hardness as response.

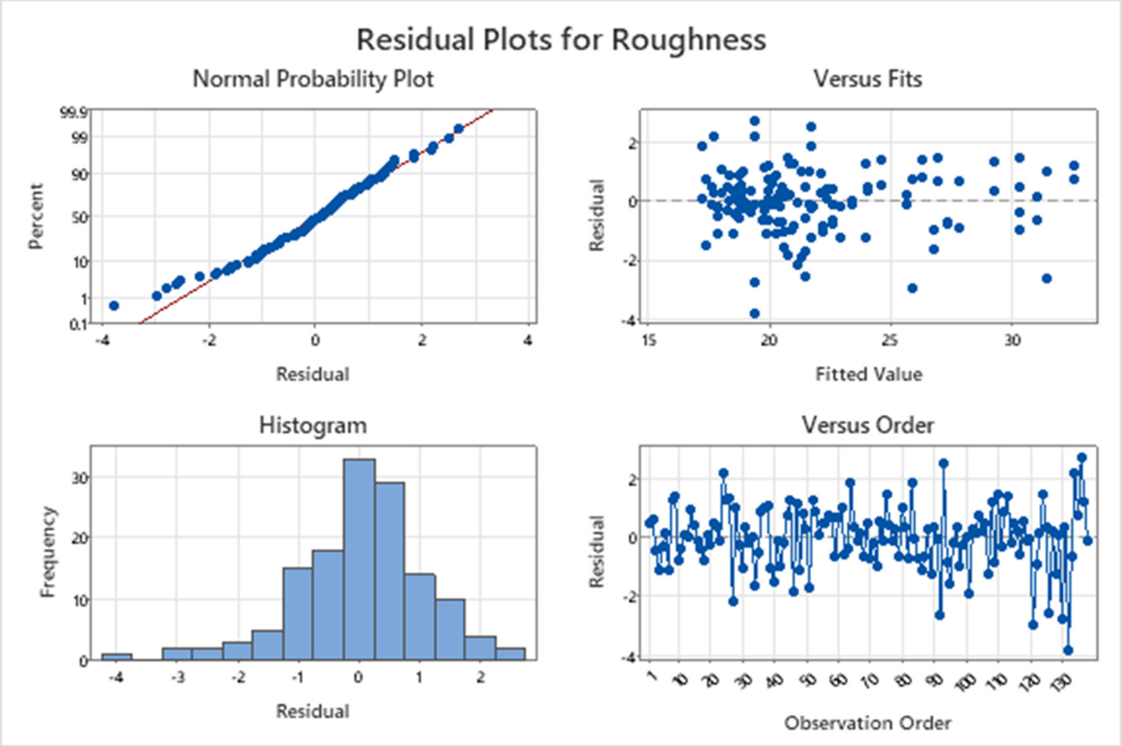

Figure S24. Residual plots for Design C2 with roughness as response.
